# Variation among 532 genomes unveils the origin and evolutionary history of a global insect herbivore

**DOI:** 10.1038/s41467-020-16178-9

**Published:** 2020-05-08

**Authors:** Minsheng You, Fushi Ke, Shijun You, Zhangyan Wu, Qingfeng Liu, Weiyi He, Simon W. Baxter, Zhiguang Yuchi, Liette Vasseur, Geoff M. Gurr, Christopher M. Ward, Hugo Cerda, Guang Yang, Lu Peng, Yuanchun Jin, Miao Xie, Lijun Cai, Carl J. Douglas, Murray B. Isman, Mark S. Goettel, Qisheng Song, Qinghai Fan, Gefu Wang-Pruski, David C. Lees, Zhen Yue, Jianlin Bai, Tiansheng Liu, Lianyun Lin, Yunkai Zheng, Zhaohua Zeng, Sheng Lin, Yue Wang, Qian Zhao, Xiaofeng Xia, Wenbin Chen, Lilin Chen, Mingmin Zou, Jinying Liao, Qiang Gao, Xiaodong Fang, Ye Yin, Huanming Yang, Jian Wang, Liwei Han, Yingjun Lin, Yanping Lu, Mousheng Zhuang

**Affiliations:** 1 State Key Laboratory of Ecological Pest Control for Fujian and Taiwan Crops, Institute of Applied Ecology, Fujian Agriculture and Forestry University, Fuzhou, 350002 China; 2 Joint International Research Laboratory of Ecological Pest Control, Ministry of Education, Fuzhou, 350002 China; 3 Department of Botany, University of British Columbia, Vancouver, BC V6T 1Z4 Canada; 4 BGI Genomics, BGI-Shenzhen, Shenzhen, 518083 China; 5 School of BioSciences, The University of Melbourne, Melbourne, VIC 3010 Australia; 6 Tianjin Key Laboratory for Modern Drug Delivery & High-Efficiency, Collaborative Innovation Center of Chemical Science and Engineering, School of Pharmaceutical Science and Technology, Tianjin University, Tianjin, 300072 China; 7 Department of Biological Sciences, Brock University, 1812 Sir Isaac Brock Way, St. Catharines, ON L2S 3A1 Canada; 8 Graham Centre, Charles Sturt University, Orange, NSW 2800 Australia; 9 School of Biological Sciences, University of Adelaide, Adelaide, Australia; 10 Instituto Superior de Formación Docente Salomé Ureña (ISFODOSU), Recinto Lus Napoleón Núñez Molina, Carretera Duarte, Km 10 1/2, Municipio de Licey Al Medio, Provincia de Santiago, República Dominicana; 11 Faculty of Land and Food Systems, University of British Columbia, Vancouver, BC V6T 1Z4 Canada; 12 Agriculture and Agri-Food Canada, Lethbridge Research Centre, Lethbridge, AB Canada; 13 Division of Plant Sciences, University of Missouri, Columbia, MO 65211 USA; 14 Plant Health & Environment Laboratory, Ministry for Primary Industries, Auckland, New Zealand; 15 Department of Plant, Food, and Environmental Sciences, Faculty of Agriculture, Dalhousie University, PO Box 550, Truro, NS B2N 5E3 Canada; 16 Natural History Museum, Cromwell Road, South Kensington, SW7 5BD London, UK; 17 Institute of Plant Protection, Fujian Academy of Agricultural Sciences, Fuzhou, 350003 China; 18 BGI-Shenzhen, Shenzhen, 518083 China; 19 James D. Watson Institute of Genome Sciences, Hangzhou, 310058 China

**Keywords:** Phylogenetics, Population genetics, Genomics, Entomology

## Abstract

The diamondback moth, *Plutella xylostella* is a cosmopolitan pest that has evolved resistance to all classes of insecticide, and costs the world economy an estimated US $4-5 billion annually. We analyse patterns of variation among 532 *P. xylostella* genomes, representing a worldwide sample of 114 populations. We find evidence that suggests South America is the geographical area of origin of this species, challenging earlier hypotheses of an Old-World origin. Our analysis indicates that *Plutella xylostella* has experienced three major expansions across the world, mainly facilitated by European colonization and global trade. We identify genomic signatures of selection in genes related to metabolic and signaling pathways that could be evidence of environmental adaptation. This evolutionary history of *P. xylostella* provides insights into transoceanic movements that have enabled it to become a worldwide pest.

## Abstract (web summary)

The diamondback moth, *Plutella xylostella* is a cosmopolitan agricultural pest. By analyzing 532 genomes from 114 populations around the world, the authors find evidence supporting a South American origin of this moth, and look for signatures of positive selection.

## Introduction

The diamondback moth, *Plutella xylostella* (L.) (Lepidoptera: Plutellidae), is the most widely distributed species among all butterflies and moths on Earth^1^. This major pest is an oligophagous herbivore of cultivated and wild cruciferous plants (Brassicaceae), including many economically important food crops such as cabbage, cauliflower, and rapeseed^1,2^. The total economic cost of its damage and management worldwide is estimated at US $4–5 billion per year^1,3^. This is the first species for which its field populations have been documented to have evolved resistance to DDT, the iconic chemical insecticide of the 1950s^4^ and to toxins of *Bacillus thuringiensis* (Bt) developed as an insecticide for control of Lepidoptera in the 1990s^5^. *P. xylostella* has now developed resistance to all major classes of insecticide and is increasingly difficult to control in the field^1^.

Although intensive research has been done on the biology, ecology and management of *P. xylostella* during recent decades^1,2,6^, our knowledge of its geographical origin and how it has become such a highly successful pest in all continents except Antarctica remains surprisingly incomplete and highly controversial^7–10^. To date, little is known about the global patterns of genomic variation in this species, which is essential for understanding the evolutionary history of *P. xylostella* together with the genetic basis of its rapid adaptation to insecticides.

In this study, we identify the origin of *P. xylostella* as South America using a global sample collection and nuclear/mitochondrial genome sequencing of all individuals, along with *COI* sequences for these and other specimens from BOLD. Further, we analyze the nuclear genomes of our specimens combined with geographical and historical information to reveal its dispersal routes and the progressive timing of global expansion. Based on the sequenced SNPs, we investigate the genomic signatures of selection to address the underlying mechanism associated with the local adaptation of this pest species.

## Results

### Global pattern of genomic variation

**Fig. 1:**
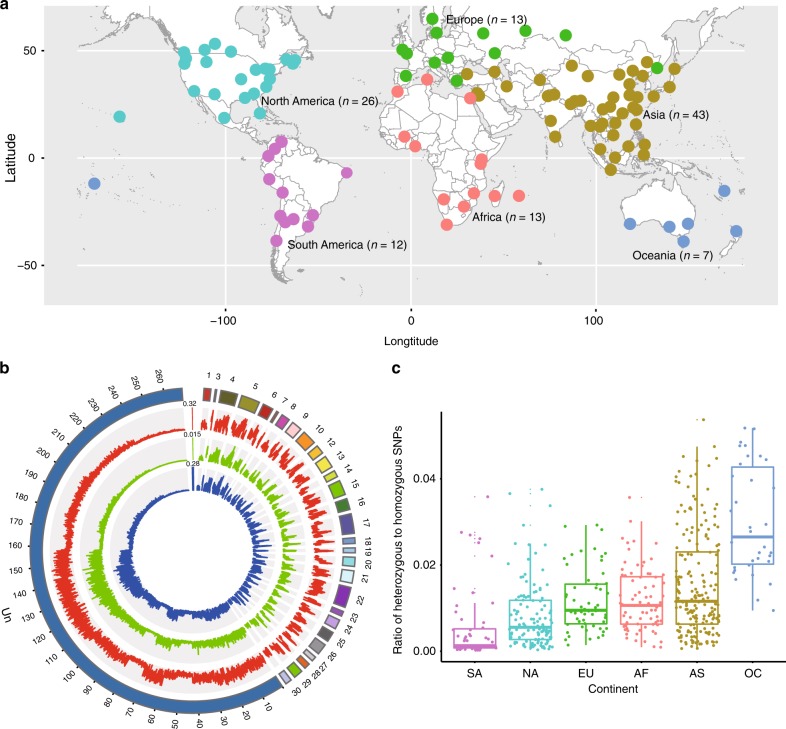
Sampling locations and genomic variation of 532 *Plutella xylostella* individuals. **a** Illustration of 114 sampling locations showing that the *P. xylostella* specimens were collected from 55 countries across six of the seven continents in the world, i.e. excluding Antarctica. **b** Global patterns of the genomic variation. Circles from the outermost to innermost represent the reference genome of *P. xylostella* (including the partial sequences of 28 chromosomes and the scaffolds that were unable to be assigned (Un)), SNPs, nucleotide diversity and indels, respectively. **c** Ratio of individual-based heterozygous to homozygous SNPs in different continents of South America (SA), North America (NA), Europe (EU), Africa (AF), Asia (AS), and Oceania (OC). Boxes show the first and third quartile range (IQR) while whiskers extend to a maximum of 1.5 * IQR. Values for each of the individuals are shown as points surrounding boxplots. Source data are provided in the Source Data file. The map was generated with the rworldmap package v1.3-6^73^.

We first characterized the global pattern of variation among 532 genomes of *P. xylostella* using a worldwide sample of specimens collected from different locations (sites) in a stratified fashion reflecting a diverse range of biogeographical regions (Fig. 1a, Supplementary Fig. 1 and Supplementary Table 1) and covering an extensive scope of the eco-climatic index (Supplementary Fig. 2). Each of the individual genomes was sequenced with the Illumina sequencing system (HiSeq 2000) to produce 90-bp paired-end raw reads (Supplementary Fig. 1). A total of 1,797 Gb quality filtered reads (Supplementary Table 2) were mapped to the *P. xylostella* reference genome^11^ using Stampy v1.0.27^12^. Individuals with low mapping rate or coverage (<60%) were excluded, and a total of 532 individuals were retained for variant discovery (Supplementary Table 2). After calibrating and filtering of the low-quality variants (Supplementary Fig. 1), we generated a genomic dataset containing 40,107,925 SNPs and 22,736,441 indels (Supplementary Tables 3 and 4), representing one variant on average in every six bp of the reference genome^11^. This is the densest variant map for any organism, including the recently released data of human^13^ and *Arabidopsis thaliana*^14^. The global pattern of genomic variation (Fig. 1b), regional diversity of individual-based SNPs (Fig. 1c), and low ratio of shared SNPs (7.20%) among different geographical populations (Supplementary Table 5) revealed a high level of polymorphism that provided the capacity for *P. xylostella* to readily expand and adapt to different habitats worldwide.

### Geographical origin

An earlier study proposed that *P. xylostella* originated from Mediterranean Europe^8^ as many Brassicaceae crops were first domesticated in the this region. Other studies predicted a South African^9^ or Chinese origin^7^ based on the regional diversity of indigenous Brassicaceae hosts or parasitoids of *P. xylostella*. An mtDNA-based analysis had supported claims of Africa as the possible area of origin of the species but used as few as 13 sampling sites worldwide without samples from South America^10^. A much larger and more representative collection of samples was required for accurate identification of the pest’s geographical origin and better understanding of the evolutionary history of *P. xylostella*.

**Fig. 2:**
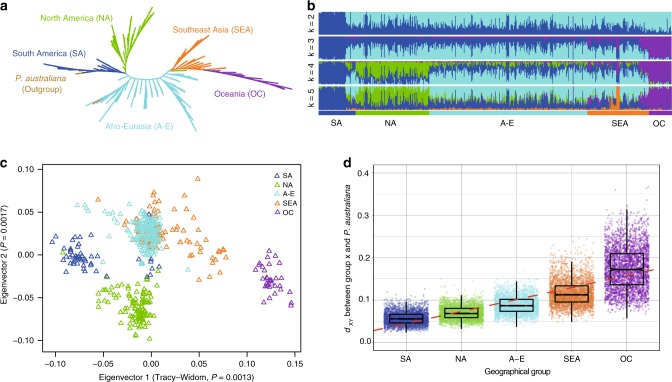
Origin and global dispersal of *P. xylostella*. **a** Neighbor-joining phylogeny based on the nuclear genomes (SNPs) of 532 *P. xylostella* individuals collected worldwide, with two congeneric *P. australiana* individuals being used as an outgroup. The branch lengths are not scaled. See also Supplementary Fig. 3. **b** sNMF-based genetic structure and individual ancestry with colors in each column representing ancestry proportion over range of population sizes (*K* = 2–5, with an optimal *K* = 5). **c** PCA plot of the first two components generated by 2,839 SNPs. **d** *d*_XY_ calculated between each *P. xylostella* group and *P. australiana* based on 3,256 non-overlapping genome-wide windows. Boxes show the first and third quartile range (IQR) while whiskers extend to a maximum of 1.5 * IQR. Values for each window are shown as individual points surrounding boxplots. A simple linear model was fitted to the data (red dashed line), the line of best fit has an estimated *R*^2^ value of 0.6149 and a slope of 0.02821. Source data are provided in the Source Data file.

We extended these efforts to conduct a genomic study with high-quality nuclear SNPs of the worldwide sample (Fig. 1a, Supplementary Fig. 1 and Supplementary Table 1). Using the nuclear SNP data, a neighbor-joining (NJ) tree was constructed for global *P. xylostella* populations with the congeneric *P. australiana* as an outgroup according to our *COI*-based phylogenetic analysis (see Methods). Results revealed that multiple individuals collected from South America (SA) formed a distinct and basal clade (Fig. 2a and Supplementary Fig. 3). This was further confirmed by mitochondrial genomic data using the same specimens (Supplementary Fig. 4) and *COI* gene data from our specimens combined with additional published data (Supplementary Fig. 5). We also generated summary trees of different groups (see population genetic structure analysis below) based on 3,256 genome-wide local trees and revealed that the most prevalent topologies across the genome support *P. xylostella* populations of South America as the basal node closest to *P. australiana* (Supplementary Table 6). These results strongly suggest that *P. xylostella* originated in South America, where other endemic *Plutella* species are known^15^. Our study convincingly repolarizes the evolutionary history of *P. xylostella* from hypotheses of Old-World origin^7–10^ to the New World.

The Brassicaceae family contains >3,700 species found on all continents but Antarctica^2,16^. In South America, with the richest cruciferous flora of the Southern Hemisphere^16,17^, *P. xylostella* would have evolved on native Brassicales. Following European colonization of South America in the late 15th to early 16th century, the introduction and widespread use of domesticated Brassicaceae crops^18–20^ would have expanded host plant resources used by *P. xylostella* on this continent. Initially, the species would have been confined to South America, isolated by oceans and limited by habitat/eco-climatic constraints until human interference^21^.

### Evolutionary and expansion history

Based on our phylogenetic analysis, in addition to the basal clade of South America (SA), we found four additional clades of North America (NA), Afro-Eurasia (A-E), South East Asia (SEA), and Oceania (OC) (Fig. 2a). These geographically clustered groups were supported by genetic structure analysis (Fig. 2b). A principal component analysis (PCA) (Fig. 2c) provided further evidence of the population structure of *P. xylostella* worldwide, with gene flow across the continent of Afro-Eurasia. The *d*_XY_-based analysis^22^ showed the lowest genetic differentiation between SA *P. xylostella* and *P. australiana*, followed by NA *P. xylostella* and *P. australiana*, A-E *P. xylostella* and *P. australiana*, SE Asian *P. xylostella* and *P. australiana*, and OC *P. xylostella* and *P. australiana* (Fig. 2d), which outlined the global colonization process of *P. xylostella* populations.

**Fig. 3:**
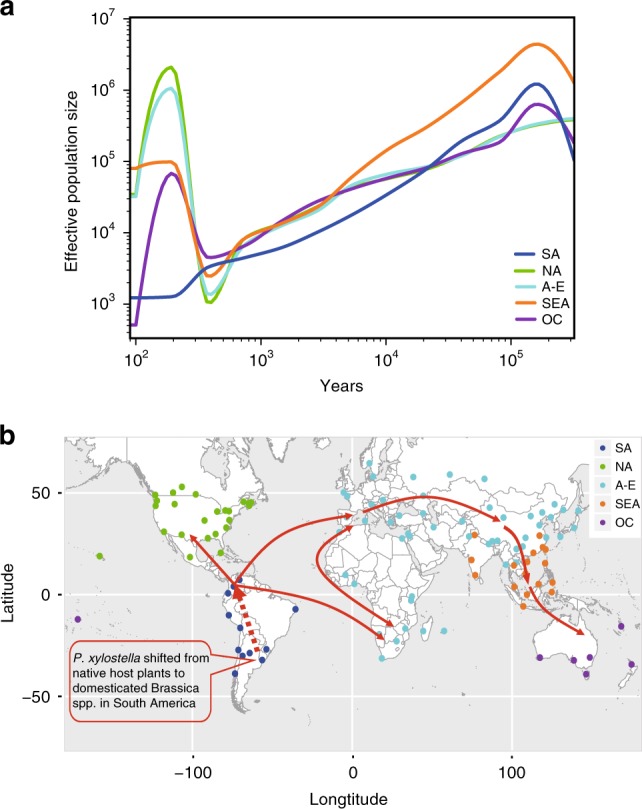
Global expansion and demographic history of the *P. xylostella* populations. **a** Demographic history of *P. xylostella* illustrating the effective population sizes and divergence times based on multiple unphased individuals from different geographical groups and predicted by SMC++^24^. Coordinates are logarithmically scaled. **b** A proposed scenario of global colonization of the *P. xylostella* populations. The red arrows denote the proposed dispersal events of *P. xylostella* from South America towards other continents based on phylogenetic result (Fig. 2a), population genetic analyses (Fig. 2b–d), and demographic history (Fig. 3a). Source data are provided in the Source Data file. The map was generated with the rworldmap package v1.3-6^73^.

To further investigate the demographic history of *P. xylostella*, we estimated the population sizes and divergence times using a pairwise sequentially Markovian coalescent (PSMC) model^23^. It revealed a strikingly concordant history among geographical groups with a sharp decline following the last glacial maximum (LGM) in the early phase of evolution and a pattern of divergence in the recent past (with low resolution) among different geographical groups (Supplementary Fig. 6). A recently published approach, SMC++ ^24^, which estimates the historical population sizes with higher resolution in the recent past compared to other methods such as PSMC^25^, was used to predict the historical population sizes and divergence times of different groups. We found that *P. xylostella* experienced three major expansions across the world, with both North American and Afro-Eurasian lineages splitting from the ancestral lineage approximately 500 years ago, followed by South East Asia and then Oceania (Fig. 3a).

*P. xylostella* has remarkable genetic plasticity^26^ and high level of genomic variation^11^ that enable it to rapidly adapt to local environments, potentially leading to the change in the levels of genetic diversity among geographical populations. After a new founder event of *P. xylostella*, the sizes of derived populations tend to rapidly grow based on the SMC++ analysis (Fig. 3a). This may have led to accumulation of mutations that were not present in the ancestral population, especially reflecting the likelihood that the species would have been subject to new and diverse selection pressures from novel plant hosts, novel agonists (e.g., pathogens and parasitoids), habitats, and climates^27,28^, as well as becoming established in extensive, heterogeneous geographical regions that limited mixing. Genetic admixture, which may generate novel genotypes^27,29^, among the *P. xylostella* populations in both the Old-World and North America was frequent according to our phylogenic reconstruction (Supplementary Fig. 3) and population structure analyses (Fig. 2b and c). Together, these effects help explain the pattern of increasing genetic diversity in the range expansion process of *P. xylostella* populations (Fig. 1c).

Our phylogenetic and genetic analyses, PCA plot of the first two components, and *d*_xy_-based analysis of differentiation (Fig. 2) supported the demographic evidence that the SA lineage was basal with the NA, A-E and SEA lineages diverging at progressively later stages and the OC lineage the most recent (Fig. 3a). These results were integrated with historical information^18–20^, allowing us to propose a scenario of dispersal events for *P. xylostella* (Fig. 3b). We found that the major expansion events of *P. xylostella* were associated with human activities of agricultural production and trade. With European colonization, particularly the domestication of cruciferous crops with reduced glucosinolates and the introduction of Brassicaceae crops by European colonizers to South America^18,30^, the original populations of *P. xylostella* in South America appear to have dispersed to and colonized various regions of the world (Fig. 3a and b). After colonizing the Mediterranean region, founder populations of *P. xylostella* likely dispersed across Europe, Western Asia, and Africa (Figs. 2 and 3)^31,32^. Like the spreading trend of *A. thaliana*, the diversity of *P. xylostella* populations in Europe and Eurasia exhibits a latitudinal pattern along the east-west axis (Fig. 3b), which has been facilitated by the rapid expansion of agriculture^14,33^. Around 200 years ago, independent dispersal events led the founder populations expanding eastwards into Asian countries first and then proceeding to Oceania (Figs. 2 and 3)^34,35^, which corresponds to the most recent major region of colonization by Europeans. Records of Brassicaceae date from the “First Fleet” arrival in Australia in 1788 that carried produce and seeds of several *Brassica* species^35^, and this was followed by introduction and widespread cultivation of other brassicas by Chinese Australians^34^. Relative to the predicted earliest time when a Plutellidae ancestor may have become a cruciferous specialist (~54–90 million years ago)^11,36^, the recent expansion events of *P. xylostella* (~200–500 years ago) further indicate that it could have survived on the indigenous Brassicaceae plants in South America for a long time, possibly in the timeframe of millions of years, after its putative divergence from an ancestor shared with its closest known relative, *P. australiana*^37^.

### Genomic signatures of local adaptation

Based on our globally sampled genomic data, we found that *P. xylostella* populations across the world exhibited a dense map of variants (Supplementary Tables 3 and 4), high level of polymorphism (Fig. 1b, c and Supplementary Fig. 7), and rapid decay of linkage disequilibrium (LD; Supplementary Fig. 8). These findings suggest a large effective population size for this species. Considering its genetic heterozygosity and rapid insecticide-resistance evolution, this species is well suited for a study of evolutionary adaptation under strong environmental selection pressure^38,39^. The intensive use of insecticides against *P. xylostella* has led to increased selection pressure for development of insecticide resistance^1,2,4,40–45^. We identified a global pattern of adaptive variation shown by the frequency distribution of three reported SNPs associated with insecticide resistance^46,47^ (Supplementary Fig. 9). Such a global genotype distribution of three insecticide-resistance-related point mutation loci revealed that selection pressure resulting from insecticide applications had strong geographical dependence.

To identify the genomic signatures of evolutionary adaptation for *P. xylostella*, we ran a genome-wide association study (GWAS) using the eigenvector1 of PCA as a “phenotype” (EigenGWAS)^48^ to isolate a group of 75 individuals from Southeast Asia and Oceania (Supplementary Fig. 10). This reflects the fact that in these tropical and subtropical regions, cruciferous crops are massively and continuously grown year-round in a variety of cropping systems from backyard gardens to large-scale farms, resulting in favorable conditions for *P. xylostella* to develop and frequently outbreak throughout the year^1,2^.

We identified 3,827 significantly differentiated SNPs (*P* ≤ 1e^−8^) (Supplementary Fig. 11a, right) with high level of genetic differentiation (*F*_ST_) from 64,960 filtered SNPs (Supplementary Fig. 11a, left), which indicated that numerical distribution the significantly differentiated SNPs was proportionally similar to that of the filtered SNPs in each of the four genomic regions (Supplementary Fig. 11a). These outliers contained 1,179 candidate genes, being the most highly represented in metabolic and molecular signaling-related pathways according to the GO and KEGG analysis (shown with top 20 GO terms and KEGG pathways; Figs. 11b and 12). Among the 1,179 candidate genes under divergent selection we found 93 that were annotated in the published *P. xylostella* genome with known functions of detoxification of plant defense compounds and insecticide resistance^11^. We then identified six genes with non-synonymous SNPs in coding regions. Three of them, including carboxypeptidase A (Px005867), P450-CYP2 (Px002515), and juvenile hormone esterase (JHE, Px003448) have reliable structural templates available in the Protein Data Bank, which allowed us to create homology models for these three enzymes using Schrödinger software^49^.

Signals of divergent selection for the three non-synonymous mutations were identified according to their global distribution of genotype frequency (Supplementary Fig. 14). Comparison of the predicted structures between wild-type (WT) and mutant (Mut) enzymes revealed the potential impacts of these three mutations on the structural changes of these enzymes (Supplementary Fig. 15), which provides a cue for further experimentally-based research to establish a functional relationship between mutations and insecticide resistance^50,51^.

## Discussion

The present study improves our understanding of the origin, evolution, and genetic bases of adaptation in *P. xylostella*, a species with worldwide importance for pest management and food safety. Using a global sample collection (532 individuals) covering all six continents where the species occurs and nuclear and mitochondrial genomes as well as *COI* sequencing of all individuals, we have identified the area of origin of *P. xylostella* as South America. The result contrasts with previous hypotheses that suggested the Mediterranean region^8^, South Africa^9,10^ or China^7^ as possible areas of origin of the species. Further, the phylogeographical profiling reveals that *P. xylostella* expansion events and timing have been facilitated by human socioeconomic activities. Genes in metabolic and molecular signaling-related pathways are putative candidates involved in evolutionary adaptation under the strong selective pressure of insecticides. Our results illustrate the utility of emerging genomic approaches to understand historical patterns of species expansion, and further address the underlying mechanisms associated with the worldwide dispersal of this notorious pest species.

## Methods

### Sample collection and DNA extraction

Based on the globally-distributed nature of *P. xylostella*, we developed a sampling plan with broad geographic scope (Fig. 1a and Supplementary Fig. 1). The global samples of *P. xylostella* were collected in 2012–2014 from 114 locations that cover broad regions throughout the world, with 13 samples from Africa including Madagascar, 43 samples from Asia, 13 samples from Europe, 26 samples from North America including Hawaii, 12 samples from South America, and 7 samples from Oceania (Fig. 1a and Supplementary Table 1). Our collection covers an extensive range of the eco-climatic index and areas that support differing numbers of annual generations, including those regions with year-round persistence of *P. xylostella* to others that are only seasonably suitable for growth and development of the species (Supplementary Fig. 2). Within each location, larvae, pupae, or adults were collected from cruciferous vegetable fields. Field-collected samples were morphologically inspected and genetically checked with *COI* sequences to confirm their identity.

The samples were preserved in 95% alcohol at −80 °C prior to DNA extraction. At least five individuals from each sampling location were used for DNA extraction. For quality control, each individual was washed twice using double-distilled water, and then dissected to remove the midgut including its microbiome and parasitoids to eliminate potential DNA contamination (Supplementary Fig. 1). To avoid unintentional biases, the individuals were each allocated a code number (Supplementary Table 2) in a double-blind fashion to obscure the origin of the insect to all handlers and analysts who identified the insect, its DNA or any associated genomic data.

DNA was extracted from each individual using DNeasy Blood and Tissue Kit (Qiagen, Hilden, Germany) following the manufacturer’s protocol. DNA was eluted from the DNeasy Mini spin column in 200 µl TE buffer. Concentration and integrity of the total DNA for each individual was measured with a Qubit Fluorometer (Invitrogen, Carlsbad, CA, USA) and agarose gel electrophoresis.

### DNA barcoding and sequencing

A *Cytochrome Oxidase I* (*COI*) mitochondrial gene fragment of up to 658 bp was amplified and sequenced using Sanger sequencing, and then queried to the BOLD system^52^ to confirm the species identity for each individual. In the 468 sequences of this dataset [10.5883/DS-PLUT1]^37,52^, 399 sequences belong to *P. xylostella* (BOLD:AAA1513 [http://www.boldsystems.org/index.php/Public_BarcodeCluster?clusteruri=BOLD:AAA1513]) and 58 to *P. australiana* (BOLD:AAC6876 [http://www.boldsystems.org/index.php/Public_BarcodeCluster?clusteruri=BOLD:AAC6876]) while the rest belongs to other potential outgroup taxa in the family Plutellidae.

Genomic sequencing was performed with Illumina HiSeq 2000 at BGI, Shenzhen, China, to produce 90 bp paired-end reads for every individual. Considering the cosmopolitan distribution of *P. xylostella*, we aimed to sequence a large number of individual genomes across various geographical locations with a 5–10× coverage for each individual, which is a strategy previously used for the 1000 human genomes project^53^ and *Apis mellifera*^54^. Two *P. australiana*^37^ individuals were also sequenced with a 30× coverage and used as an outgroup for comparative analysis of genetic differentiation with the *P. xylostella* populations and construction of the phylogenetic tree. Sequencing libraries for each of the *P. xylostella* individuals were constructed according to the manufacturer’s protocol. The quality and yield of the library were tested using the Agilent 2100 Bioanalyzer and ABI StepOnePlus Real-Time PCR System.

### Data filtering, mapping and SNP calling

Raw reads were processed to obtain clean reads using custom scripts. Poor reads with 10 ambiguous “N” bases, >40% low-quality bases, or identical sequences at the two ends were filtered out.

We artificially allocated scaffolds of the *P. xylostella* genome into 20 synthetic chromosomes, and the SNP calling, and subsequent analyses were all performed on these 20 “chromosomes”. Stampy (v1.0.27)^12^ was employed to map the clean reads onto our *P. xylostella* reference genome (v2)^11^ using default parameters. Subsequently, alignments for each of the individual samples were sorted with SortSam of Picard-tools (v-1.117, https://sourceforge.net/projects/picard/), and processed by removing duplicate reads with MarkDuplicates of Picard-tools. Reads with indels were realigned using RealignerTargetCreator and IndelRealigner in the Genome Analysis Toolkit (GATK v-3.2.2)^55^ to avoid misalignment around indels. After realignment, base-quality scores were recalibrated using BaseRecalibrator based on the reference SNP set, which was generated using UnifiedGenotyper and SAMtools^56^ from the 532 individuals. The sequencing and mapping statistics are summarized in Supplementary Table 2.

SNP calling was then performed using the GATK HaplotypeCaller with parameters --emitRefConfidence GVCF --variant_index_type LINEAR --variant_index_parameter 128,000. VariantRecalibrator was first used to create a Gaussian mixture model to examine the annotation values over a high-quality subset previously generated by UnifiedGenotyper and SAMtools and evaluate all input variants. ApplyRecalibration was used to designate the model parameters for each of the variants. Finally, VariantFiltration was used to filter the SNPs. The filtering settings were as follows: QD < 2.0 | | MQ < 40.0 | | ReadPosRankSum < −8.0 | | FS > 60.0 | | HaplotypeScore > 13.0 | | MQRankSum < −12.5.

To resolve the origin of *P. xylostella*, two congeneric *P. australiana* individuals were used as an outgroup species. The sequencing reads of *P. australiana* were aligned to the *P. xylostella* genome using Stampy (v1.0.27)^12^ with default parameters and reordered and sorted by Picard (https://broadinstitute.github.io/picard/). SOAPsnp (http://soap.genomics.org.cn/soapsnp.html) was then used to detect SNPs in each *P. australiana* individual with at least three supporting reads, and to assemble the consensus sequence for the *P. australiana* individuals based on the alignment of the sequencing reads with the *P. xylostella* genome. The genomic dataset of two *P. australiana* individuals and 532 *P. xylostella* individuals was used for phylogenetic tree construction.

To identify mitochondrial variants of *P. xylostella*, we also called SNPs using the mitochondrial genome of *P. xylostella* (GenBank KM023645 [https://www.ncbi.nlm.nih.gov/search/all/?term=KM023645]) as a reference. The same SNP calling procedure as done for nuclear SNP calling was employed, while a haplotype setting was used. The mitochondrial genome of *P. australiana* was reconstructed using MITObim^57^ with a *P. australiana COI* barcode sequence as the seed and the *P. xylostella* mitochondrial genome as the reference.

### Construction of the phylogenetic trees

Phylogenetic relationships of nuclear and mitochondrial genomes were analyzed among 532 individual samples of *P. xylostella* with two samples of *P. australiana* used as an outgroup. A phylogenetic tree based on the nuclear genomes was constructed (Fig. 2a and Supplementary Fig. 3), using the neighbor-joining (NJ) method^58^, based on a genetic distance matrix (https://github.com/BGI-shenzhen/VCF2Dis), and calculated by the software PHYLIP v3.695 (http://evolution.genetics.washington.edu/phylip.html). Mitochondrial genomes were also used for phylogenetic tree construction using the NJ method implemented in PHYLIP, and a frequency tree (Supplementary Fig. 4) was generated using the consensus module with 1000 bootstraps.

To further confirm the origin and evolutionary relationships of *P. xylostella* populations based on nuclear and mitochondrial SNPs, a *COI*-based phylogenetic tree (Supplementary Fig. 5) was constructed based on NJ method with 1000 bootstraps using MEGA5^59^. This tree included the sequences of 532 *P. xylostella* individuals collected worldwide and two *P. australiana* individuals collected in Australia, as well as individual sequences of five non-Australian *Plutella* species (with two individual sequences for each of *P. armoraciae*, *P. porrectella*, *P. geniatella*, *P. hyperboreella* and one of *P. notabilis*), *Eidophasia vanella*, *P. australiana*, and *P. xylostella* downloaded from BOLD [10.5883/DS-PLUT1]^37,52^, and two undescribed taxa (‘kaloko’ and ‘napoopoo’) from Hawaii^60,61^ downloaded from GenBank, with accession codes AF019041 [https://www.ncbi.nlm.nih.gov/nuccore/AF019041] and AF019042 [https://www.ncbi.nlm.nih.gov/nuccore/AF019042].

### Population genetic pattern analysis

Bi-allelic SNPs presenting >95% individuals with a minor allele frequency of over 0.2 in the dataset were kept using vcftools^62^ and used for population genetic structure analysis. We sampled one SNP from a 25 bp DNA window to generate loci independent of linkage disequilibrium. A total of 2,839 SNPs was retained for further analysis. The population genetic structure was analyzed using sNMF^63^ with the pre-defined genetic clusters increased from *K* = 2 to *K* = 8 and illustrated with POPHELPER^64^. Principal component analysis (PCA) was also conducted using PLINK^65^ with the same dataset, and a Tracy-Widom test was used to determine the significant level of the eigenvectors. The results (Fig. 2b) further confirmed and supported the five geographically clustered groups of *P. xylostella* populations worldwide based on previous nuclear phylogenetic analysis.

We generated genome-wide summary trees of different groups based on local trees. Variants with a maximum missing rate of 70% were filtered, and then converted to Genomic Data Structure (GDS) using SeqArray^66^. Local genetic distance matrix was calculated using R scripts (https://github.com/CMWbio) with a bin of 5,000 SNPs in a maximum interval of 100 kb. A total of 3,256 local trees were generated across the genome. TWISST^67^ was then used to calculate topology weighting for each local tree with 1,000 iterations (Supplementary Table 6). We also calculated *d*_XY_ values^22^ in these 3,256 windows between five identified groups and the outgroup population to investigate genetic differentiation pattern during the global colonization of *P. xylostella* (Fig. 2d).

We presented the global genotype distribution of three previously reported SNPs associated with insecticide resistance^46,47^ to show the geographical dependence of these point mutation loci (G4946E, L1014F and T929I; Supplementary Fig. 9). G4946E in ryanodine receptor was involved in resistance to diamide^46^, and L1014F and T929I in sodium channel were associated with resistance to pyrethroid^47^.

### Demographic history

We selected one individual with high sequencing depth from each group to estimate the demographic history of *P. xylostella* using Pairwise Sequentially Markovian Coalescence (PSMC)^23^, with a generation time of 0.1 years and a mutation rate^68^ of 8.4×10^−9^ (Supplementary Fig. 6). A recently published approach with higher resolution in the recent past compared to PSMC accuracy, SMC++^24,25^, was used to predict the demographic history (or population sizes and divergence times) of *P. xylostella* based on multiple unphased individuals (Fig. 3a). Five previously defined groups (or clades) were used for the analysis. We used a mutation rate of 8.4 × 10^−9^ from *Drosophila*^68^, and 10 generations of *P. xylostella* per year estimated with global observations and records^1–3^. The short generation time of *P. xylostella* makes possible the reliable and precise estimation of effective population sizes in the recent past using the method of SMC++^25^.

### Identification of the loci under selection

An approach of genome-wide association study with the first eigenvector from the PCA as a “phenotype” (EigenGWAS)^48^ was recently developed to identify single SNPs that contribute to the genetic differentiation (eigenvector) of two populations based on regression analysis. By using individual-level eigenvectors as phenotypes (Y in regression analysis) and single SNPs (X in regression analysis) in a linear regression, the resulting regression coefficients are equivalent to singular value decomposition (SVD) SNP effects and used to identify loci under selection along gradients of ancestry^48^. EigenGWAS also used a correction parameter to filter out signals of population stratification (i.e. caused by geography/drift), which allows the loci under selection to be identified. This approach has been successfully used to identify the loci under divergent selection between the UK and Dutch populations of great tit (*Parus major*) for better understanding of how genetic signatures of selection translate into variation in fitness and phenotypes^38^.

To identify the genomic signatures of selection for *P. xylostella*, 64,960 SNPs (Supplementary Fig. 11a) were obtained after filtering with a missing rate ≤5% and a minor allele frequency ≥0.05 by vcftools^62^ from the genomic dataset of our global samples. Based on the filtered SNPs, we ran the approach of EigenGWAS using a stringent level of genome-wide significance threshold (*P* ≤ 1e^−8^)^69^. A total of 3,827 loci (or outlier) under selection (Supplementary Fig. 11a) were identified for further functional annotation.

Based on the genomic database of *P. xylostella* (http://iae.fafu.edu.cn/DBM/ index.php), we searched for candidate genes with the outliers. GO annotation and classification of the candidate genes were conducted using Blast2GO (version 2.5.0)^70^ and WEGO^71^. Pathways of the candidate genes were identified (Supplementary Fig. 11b) using the KEGG database (http://www.genome.jp/kegg/pathway.html). The genes enriched in the first 20 GO terms and KEGG pathways, and the value of fixation index (*F*_ST_) was calculated for each of the identified loci using vcftools^58^ (Supplementary Figs. 11b; 12 and 13).

### Homology modeling

The structural models for the wild-type carboxypeptidase A, P450-CYP2 and juvenile hormone esterase were created by Prime module of Schrödinger software using human carboxypeptidase structure (PDB ID: 1PCA), fish cytochrome P450 structure (PDB ID: 4R1Z) and human acetylcholinesterase structure (PDB ID: 4BDT) as templates, respectively (Supplementary Fig. 15). The resistant-mutant models were developed by introducing mutations to the wild-type structural models, followed by further energy minimization using Chimera^72^. All of the structural figures were also generated by Chimera^69^.

### Reporting summary

Further information on research design is available in the Nature Research Reporting Summary linked to this article.

## Supplementary information

Supplementary Information

Peer Review

Reporting Summary

## Data Availability

Raw reads of all 532 sequenced individuals have been deposited in the CNSA (https://db.cngb.org/cnsa/) of CNGBdb with the accession code CNP0000018, and been synchronously deposited in the EMBL Nucleotide Sequence Database (ENA) (https://www.ebi.ac.uk/ena) with the accession code PRJEB24034. Sequences of five non-Australian *Plutella* species (with two individual sequences for each of *P. armoraciae*, *P. porrectella*, *P. geniatella*, *P. hyperboreella* and one of *P. notabilis*), *Eidophasia vanella*, *P. australiana*, and *P. xylostella* were downloaded from BOLD [10.5883/DS-PLUT1]^37,52^. Sequences of two undescribed taxa (‘kaloko’ and ‘napoopoo’) from Hawaii^60,61^ were downloaded from the GenBank, with accession codes AF019041[https://www.ncbi.nlm.nih.gov/nuccore/AF019041] and AF019042 [https://www.ncbi.nlm.nih.gov/nuccore/AF019042]. The source data underlying Figs. 1b, c, 2b–d, 3a as well as Supplementary Figs. 2, 6, 7, 8, 9, 10, 11, 12, 13, 14 and15 are provided as a Source Data file.

## Source data

Source Data
