# Supplementary material for: Variation among 532 genomes unveils the origin and evolutionary history of a global insect herbivore
Source: Nat Commun. 2020 May 8;11:2321. doi: 10.1038/s41467-020-16178-9 (PMC7211002; doi:10.1038/s41467-020-16178-9)
Supplement: Supplementary file 1 — Supplementary Information [file 41467_2020_16178_MOESM1_ESM.pdf]

Supplementary information for

Variation among 532 genomes unveils the origin and evolutionary history of a global herbivore

You et al.

Supplementary Figures and Tables

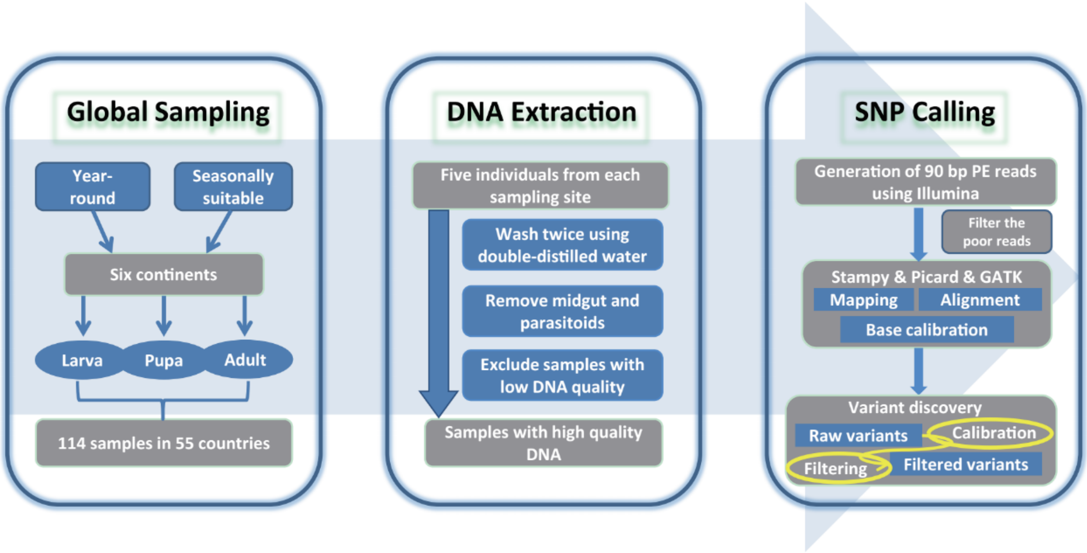

Supplementary Figure 1 Methodological framework for the quality control of global sample collection, DNA extraction, and SNP calling.

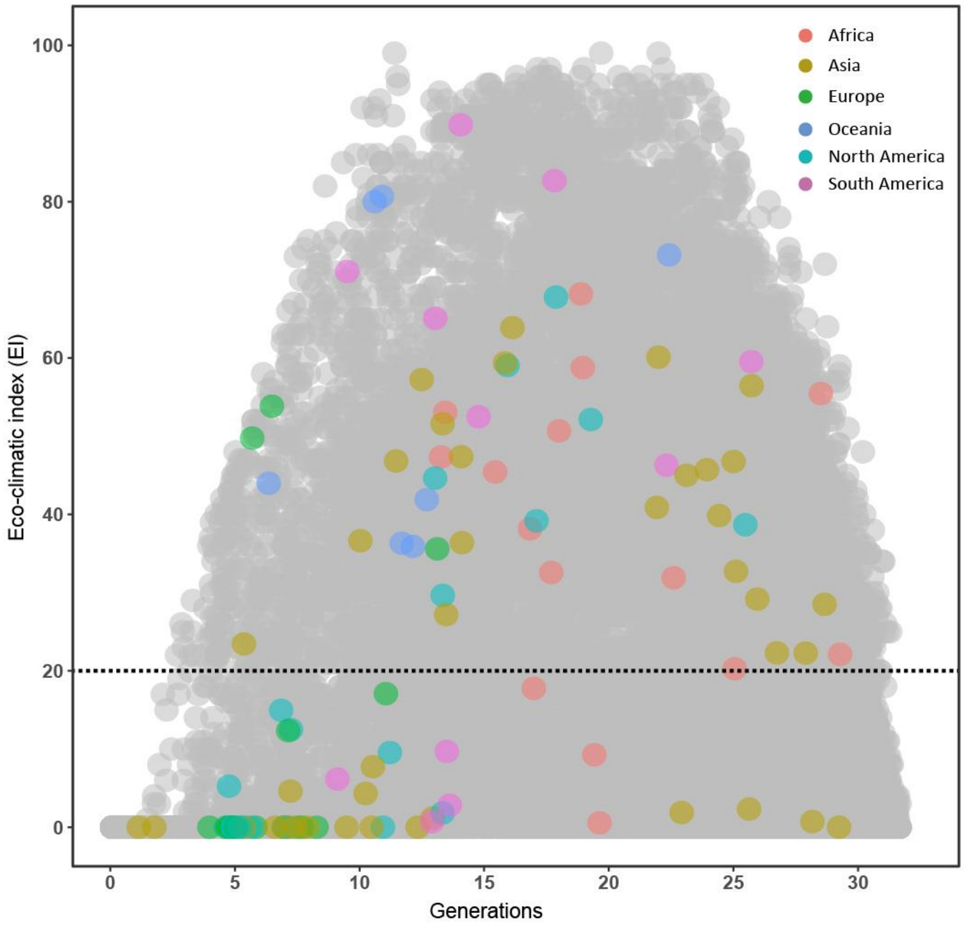

Supplementary Figure 2 Coverage of the sampling locations in a wide scope of the previously recorded distribution sites of *P. xylostella*. The sampling locations range from the regions with year-round persistence (EI ≥ 20) to others that are seasonably suitable for growth (EI < 20) of *P. xylostella*. Grey circles represent previously recorded distribution sites and other coloured circles represent sampling locations in six different continents (corresponding to Fig. 1a); and data on the recorded distribution sites, eco-climatic index and the number of annual generations were obtained courtesy of Dr Zalucki[1]. Source data are provided in file SourceDataSupplementary of Source Data.

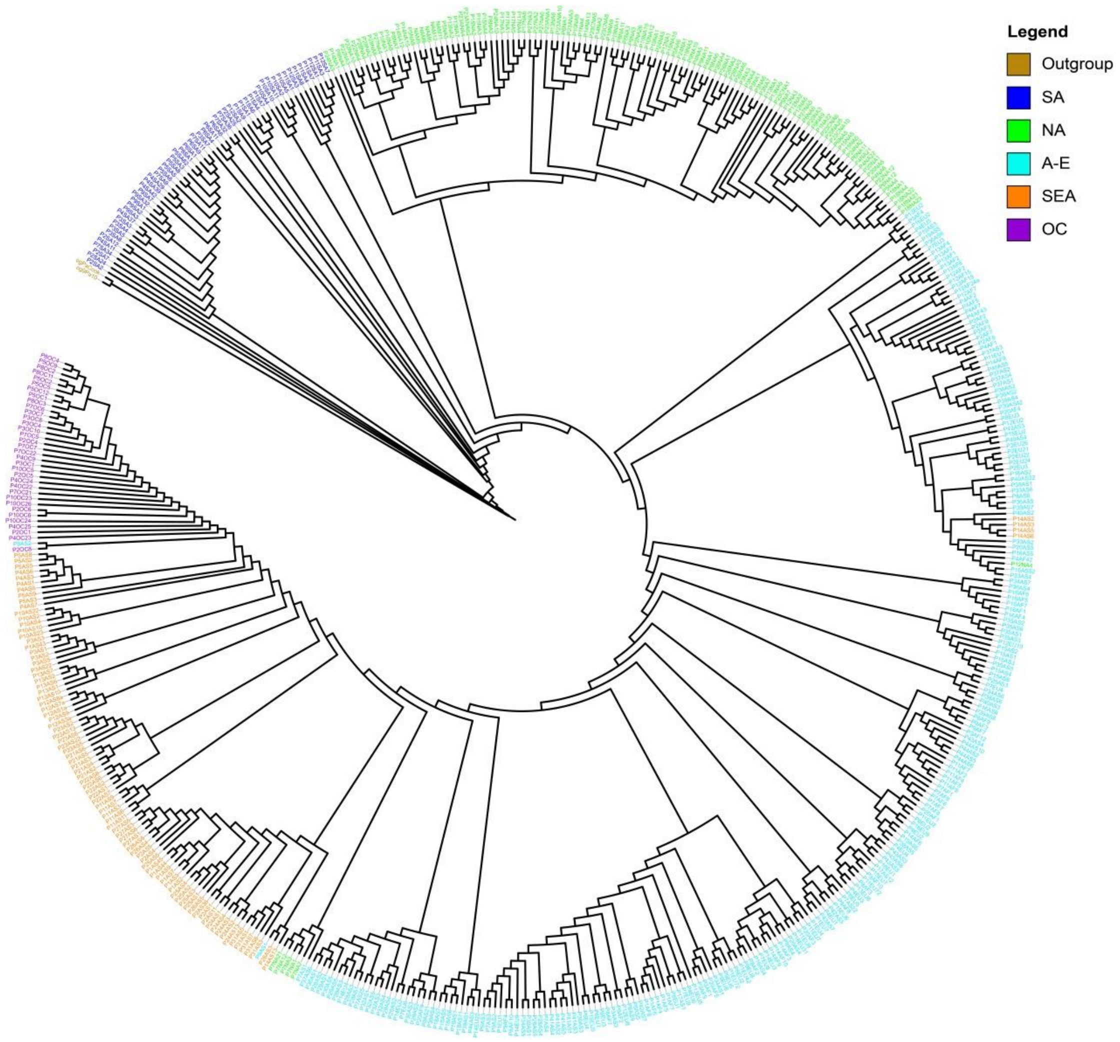

Supplementary Figure 3 Neighbour-joining phylogeny of *P. xylostella* based on the nuclear genomes of globally collected specimens, with two congeneric *P. australiana* individuals used as an outgroup. Individual sample codes (or IDs) are shown in Supplementary Table 2 and coloured to represent different geographical groups of *P. xylostella* (SA: South America; NA: North America; A-E: Afro-Eurasia; SEA: South East Asia; OC: Oceania).

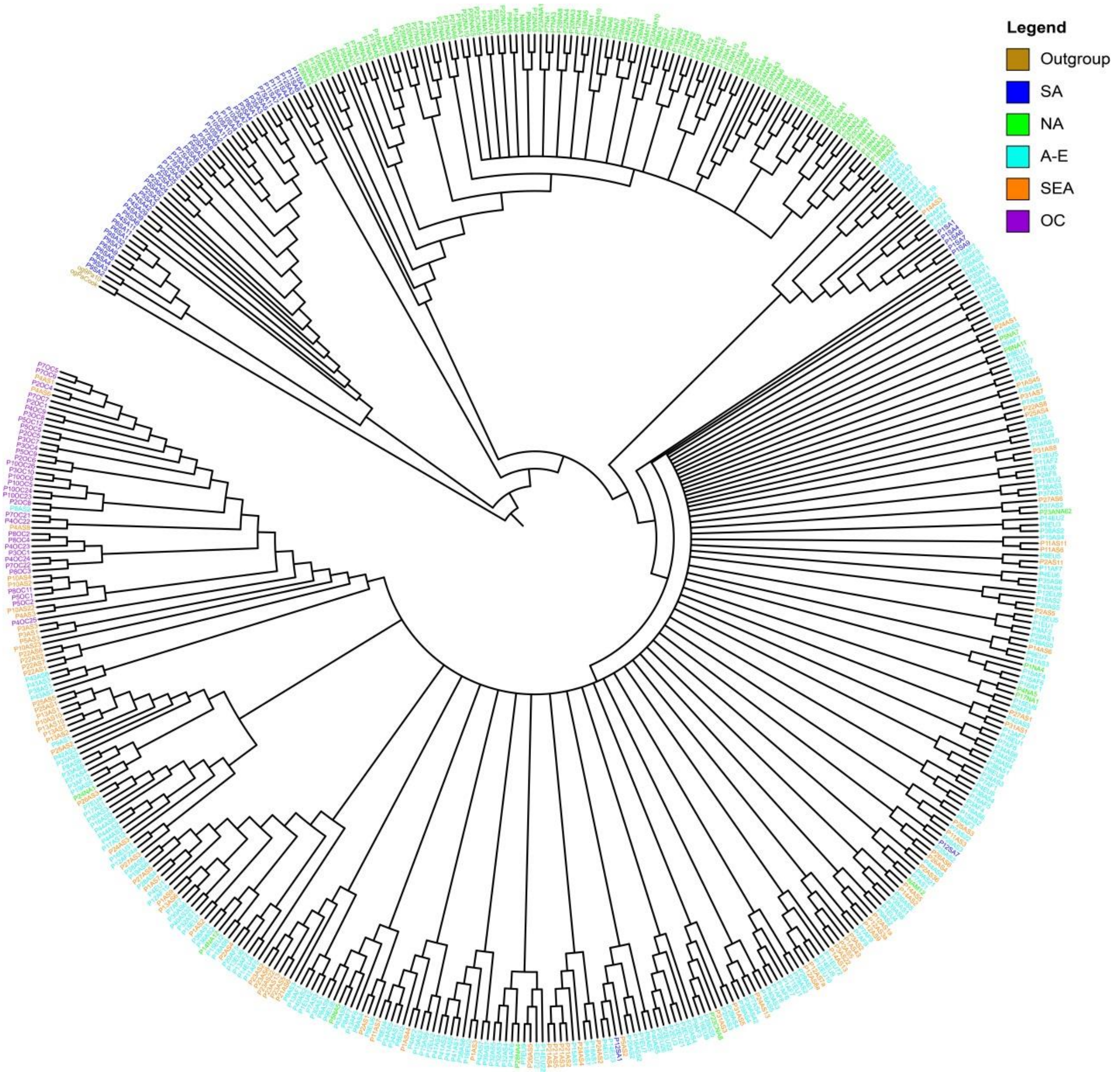

Supplementary Figure 4 Neighbour-joining phylogeny of *P. xylostella* based on the mitochondrial genomes of globally collected specimens, with two congeneric *P. australiana* individuals used as an outgroup. Individual sample codes and colours correspond to Supplementary Figure 3.

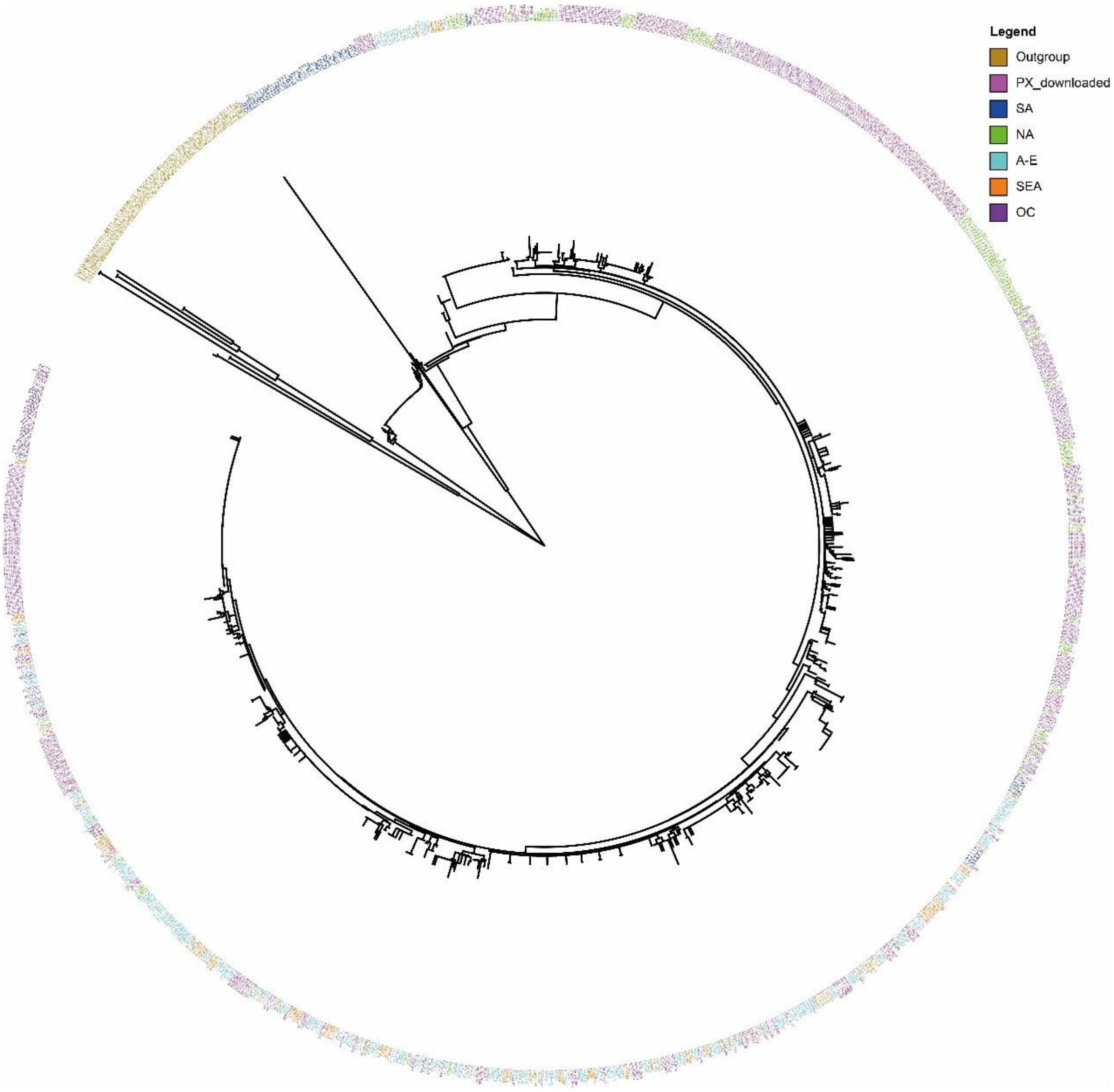

Supplementary Figure 5 Neighbour-joining phylogeny of *P. xylostella* based on the *COI* sequences of globally collected specimens. This tree includes the sequences of the 532 *P. xylostella* individuals collected worldwide and two *P. australiana* individuals collected in Australia, as well as individual sequences of five non-Australian *Plutella* species (with two individual sequences for each of *P. armoraciae*, *P. porrectella*, *P. geniatella*, *P. hyperboreella* and one of *P. notabilis*), *Eidophasia vanella*, *P. australiana* and *P. xylostella*[2] downloaded from BOLD (dx.doi.org/10.5883/DS-PLUT1), and two undescribed taxa ('kaloko' and 'napoopoo') in Hawaii[3,4] downloaded from GenBank (AF019041-AF019042). Individual sample codes and colours correspond to

Supplementary Figure 3. Px_downloaded represents the *COI* sequences of *P. xylostella* downloaded from BOLD and GenBank.

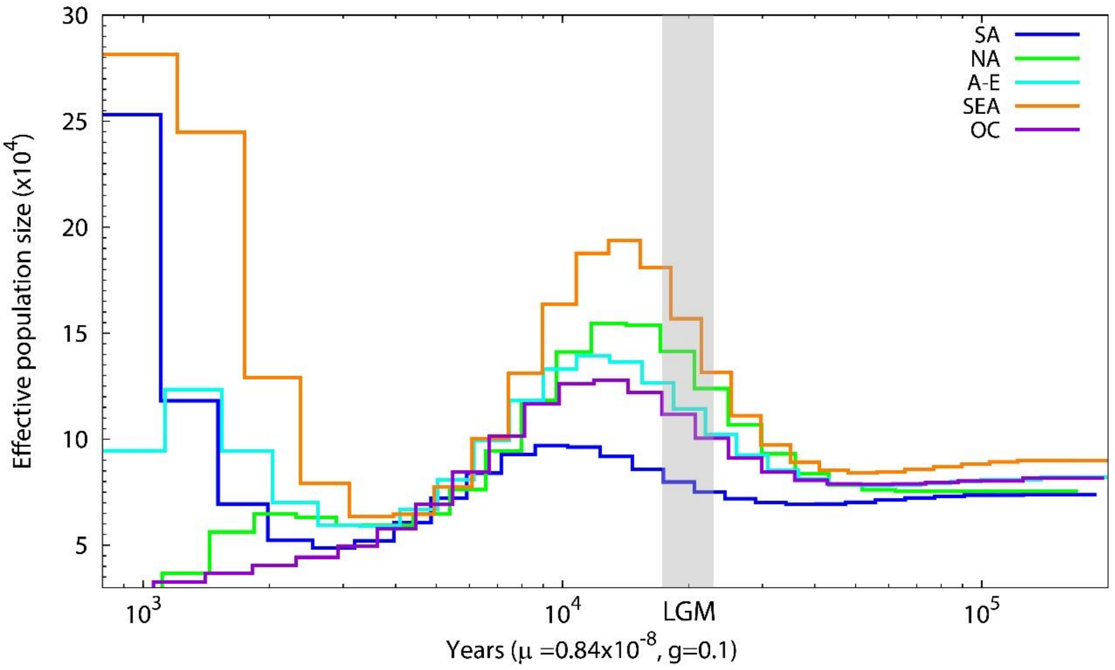

Supplementary Figure 6 Demographic history of *P. xylostella* illustrating the effective population sizes and divergence times of different geographical groups estimated using a pairwise sequentially Markovian coalescent (PSMC) model[5]. We selected one individual with high sequencing depth from each group to estimate the demographic history of *P. xylostella*. The grey shade denotes the period of the last glacial maximum (LGM, ~ 20,000 years ago). Coloured curves represent demography histories of *P. xylostella* from different geographical groups. To facilitate calculation, both horizontal and vertical coordinates are logarithmically scaled. Source data are provided in the Source Data file.

a

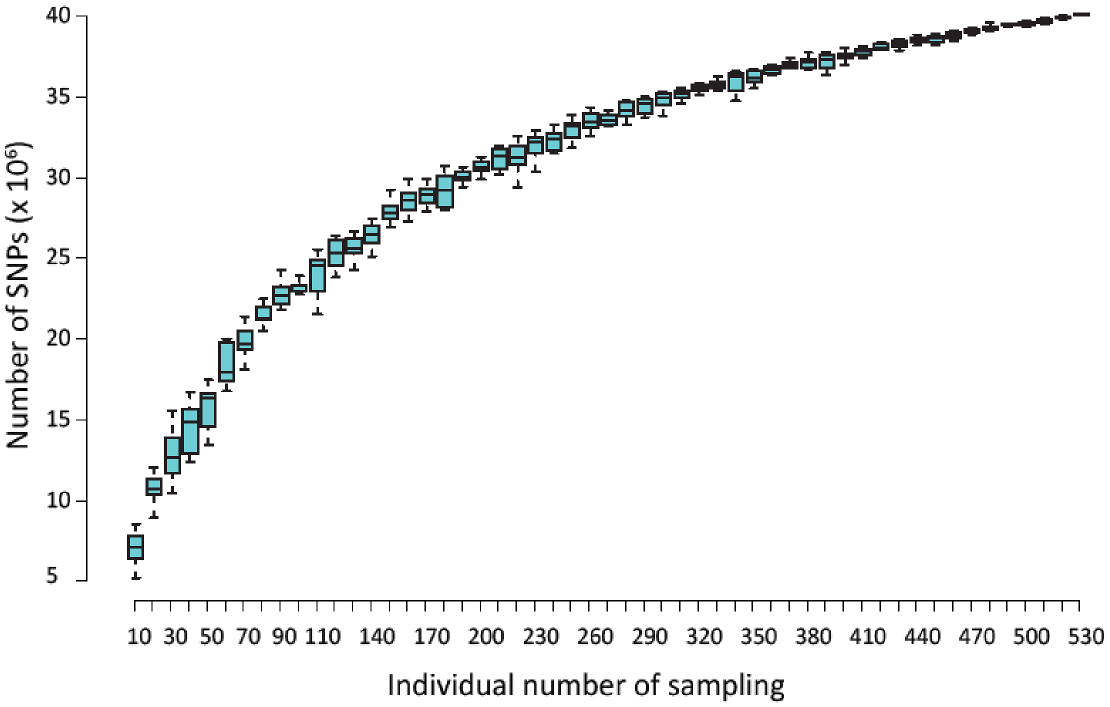

b

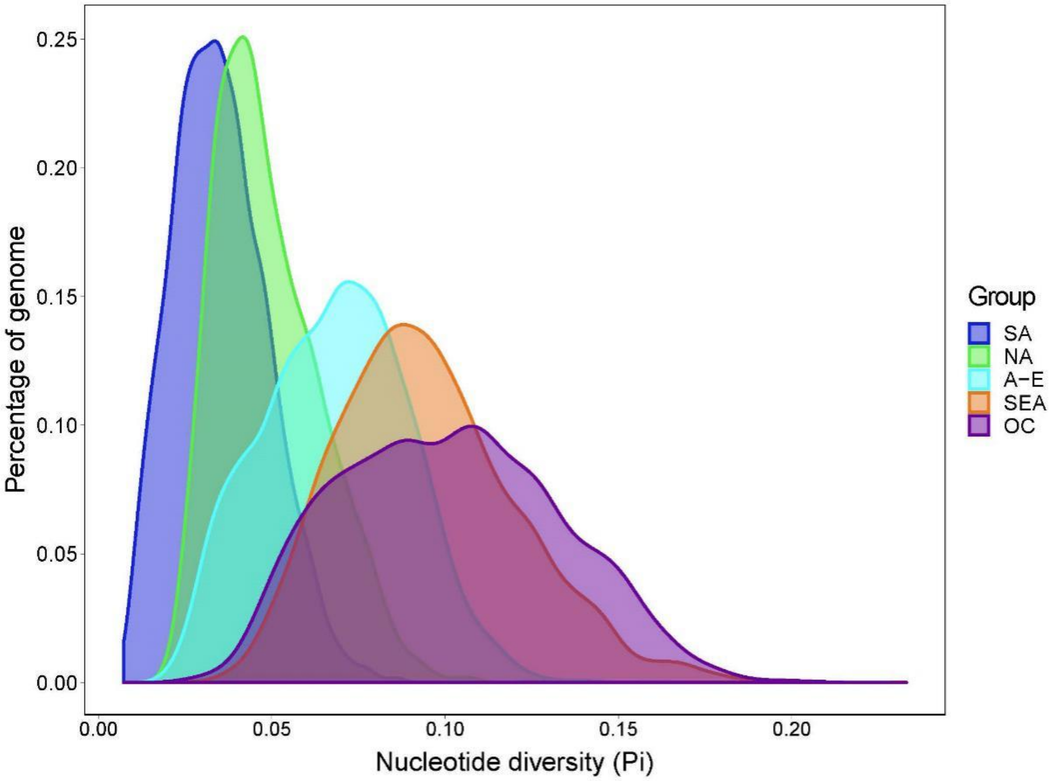

Supplementary Figure 7 Statistics of genomic polymorphism. a, SNP saturation curve based on independent samplings from the *P. xylostella* individuals collected

worldwide. Each sampling was performed with five replicates and the relevant numbers of SNP computed, scaling up by an increment of 10 individuals. The boxplot shows the SNP variation and deviation of five replicates for each sampling. Boxes show the first and third quartile range (IQR) while whiskers extend to a maximum of 1.5 * IQR. **b,** Nucleotide diversity (Pi) in the five geographically clustered groups. Source data are provided in the Source Data file.

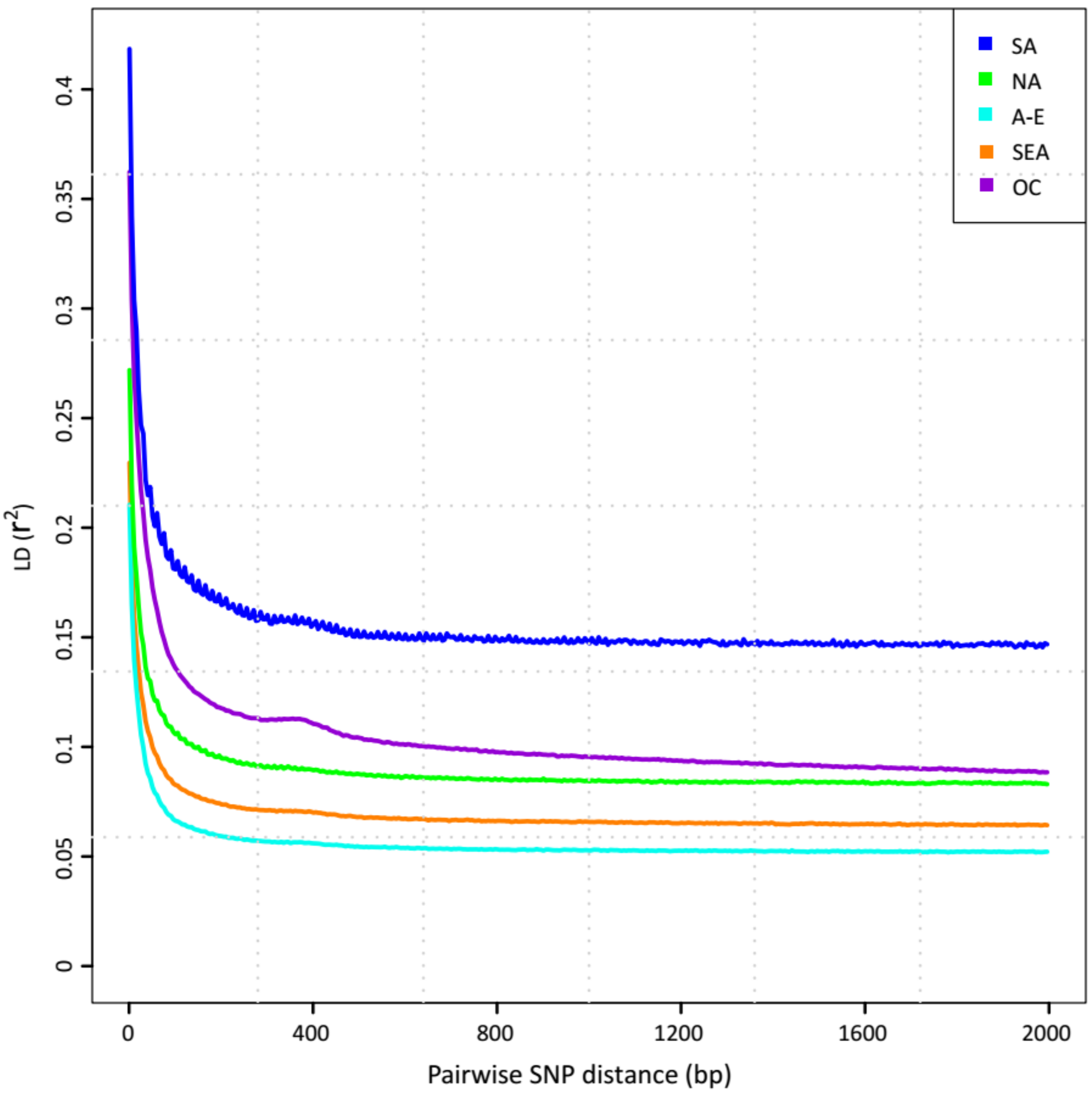

Supplementary Figure 8 Linkage-disequilibrium (LD) patterns measured with the squared coefficient of correlation (r^2) of alleles at any two loci using PopLDdecay[6] based on the *P. xylostella* genome-wide SNPs from different clusters. LD values decay rapidly with r^2 decreasing to half of its maximum value at distances of 16-35 base pairs for the five geographically clustered groups (SA: 35bp, r^2=0.2468; NA: 24bp, r^2=0.1667; A-E: 16bp, r^2=0.1322; SEA: 21bp, r^2=0.1428; OC: 32bp, r^2=0.2121). Source data are provided in the Source Data file.

A

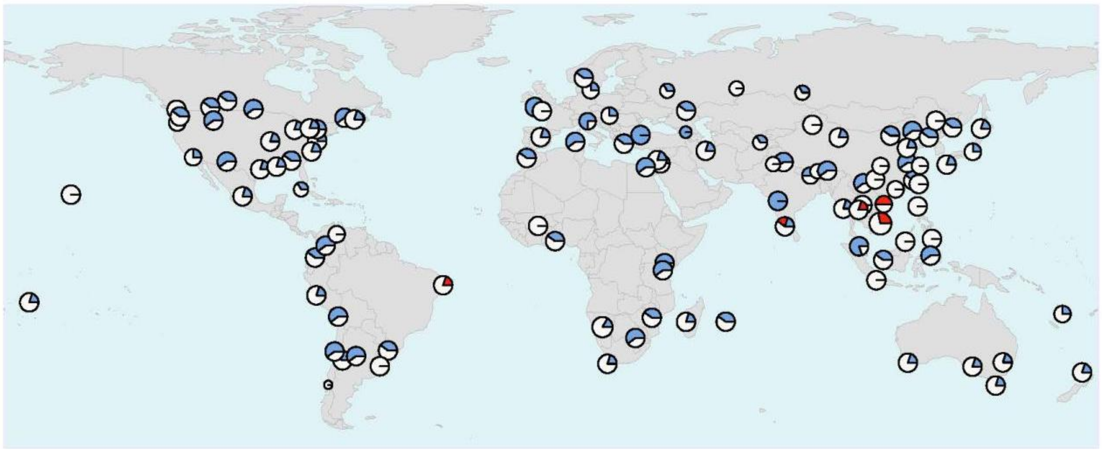

B

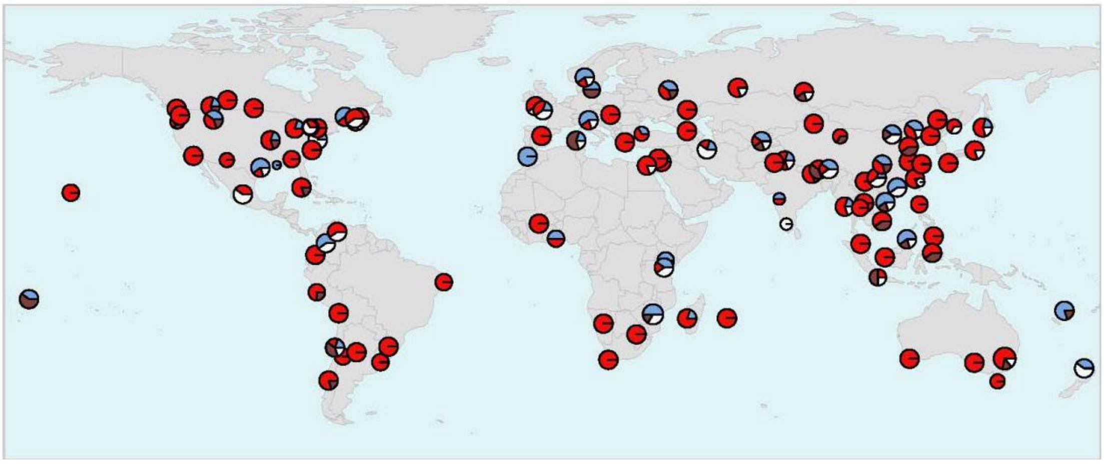

C

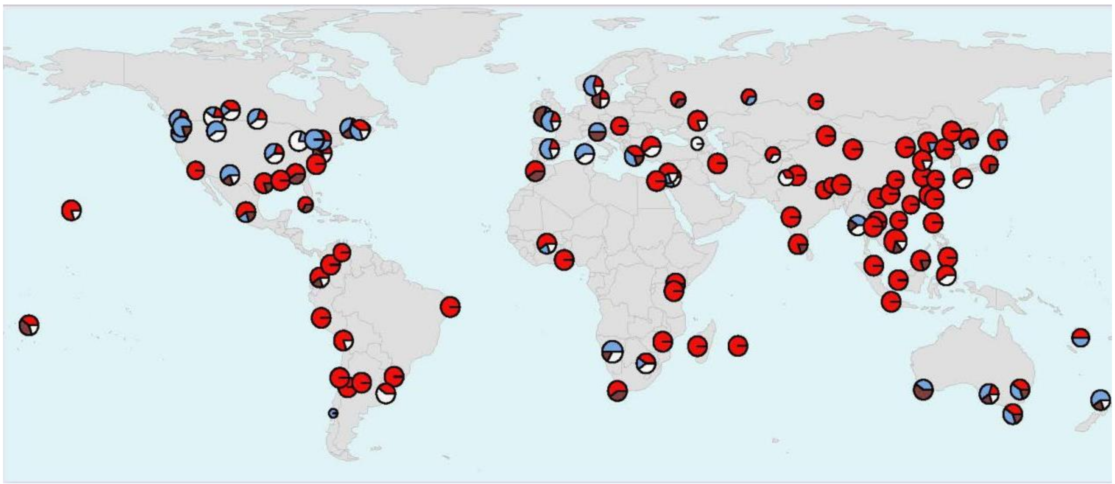

Supplementary Figure 9 Global pattern of adaptive variation shown by the frequency distribution of three reported SNPs associated with insecticide

resistance[7,8]. Blue, red, grey and white colours represent the original genotype, homozygous mutant, heterozygous mutant, and missing site respectively. **a**, ryanodine receptor (G4946E), **b**, and **c**, sodium channel (L1014F and T929I). Source data are provided in the Source Data file. The maps were generated with the rworldmap package v1.3-6[9].

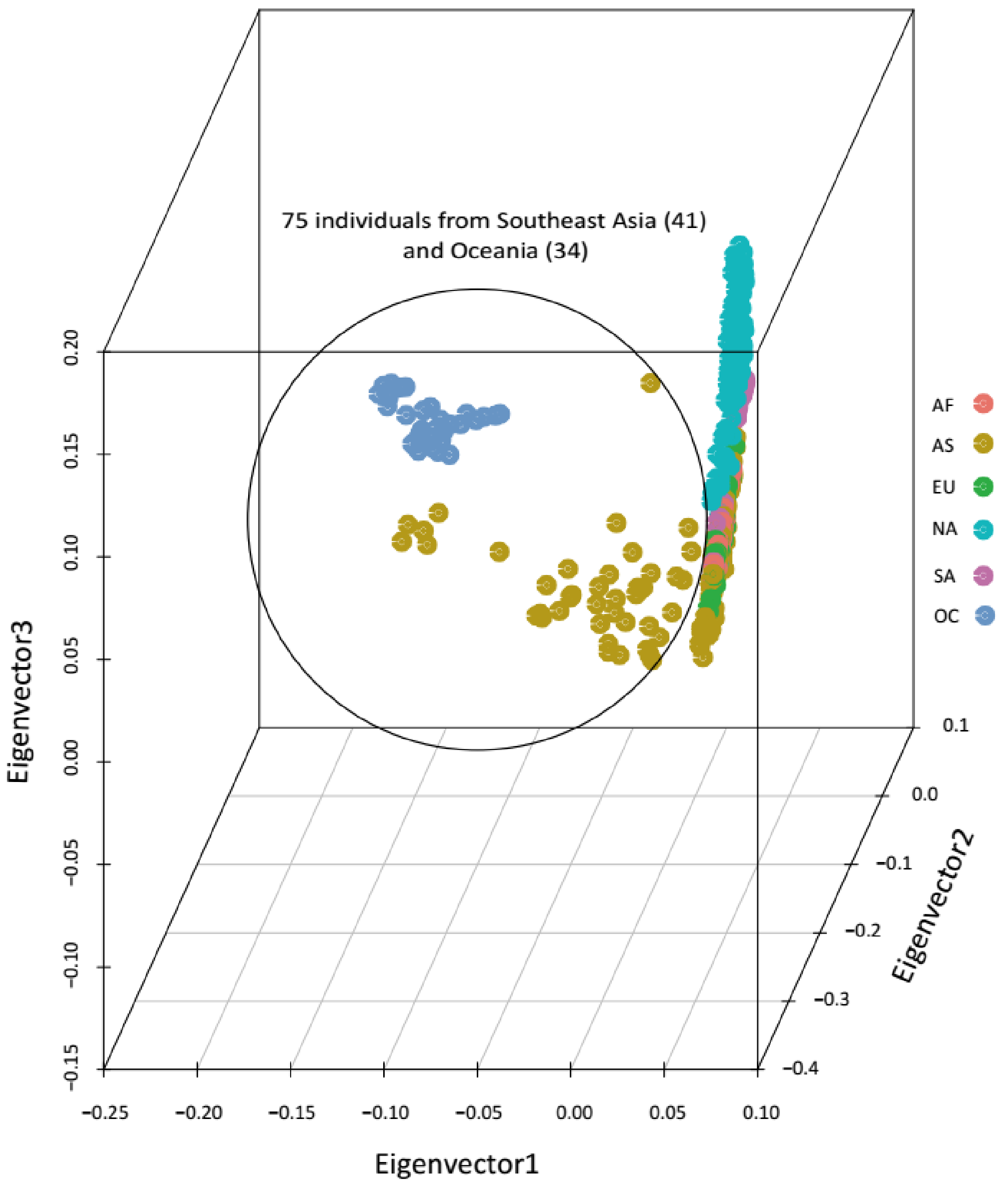

Supplementary Figure 10 First three eigenvectors of the principal component analysis using EigenGWAS package[9]. 75 individuals from Southeast Asia and Oceania were separated from other individuals by eigenvector1. AF: Africa, AS: Asia, EU: Europe, NA: North America, SA: South America, and OC: Oceania. Source data are provided in the Source Data file.

a

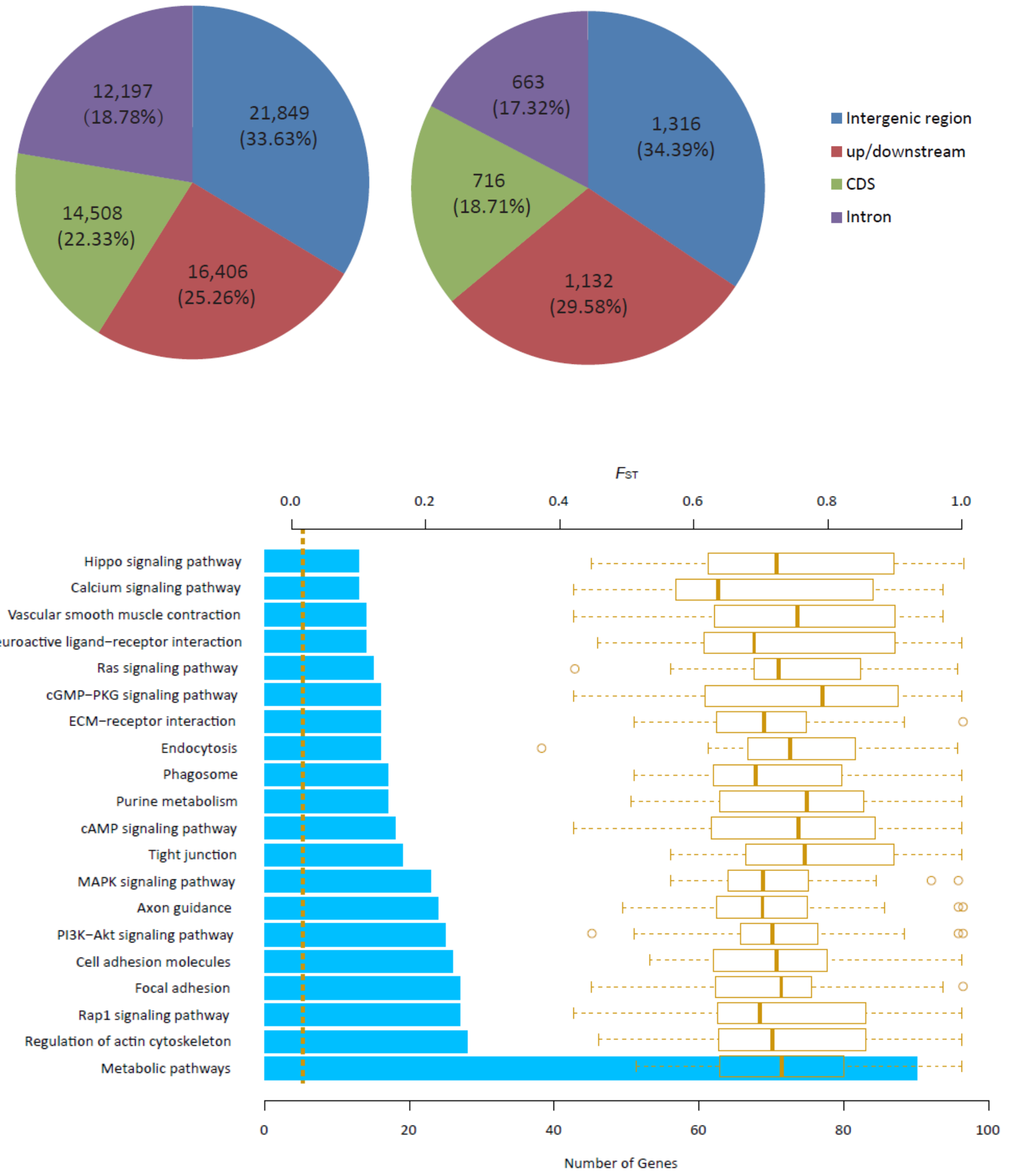

Supplementary Figure 11 Genomic signatures of adaptation. a, Numerical distribution of the filtered SNPs (left) and significantly differentiated SNPs (right) across the genome. Significantly differentiated SNPs were identified by eigenvector1 using EigenGWAS[10] with a PGC threshold of 1e^{-8}. Up/downstream indicates nucleotide sequences within a length of 5kb up and down streams for each of the genes. **b**, Representation of top 20 KEGG pathways of the genes containing

significantly differentiated SNPs. The boxplots illustrate the range of largest F_{ST} values in every single gene identified by EigenGWAS, with a median of F_{ST} value of 0.0169 (shown by a yellow dotted line). Boxes show the first and third quartile range (IQR) while whiskers extend to a maximum of 1.5 * IQR. Values for each of the outliers are shown as empty circles. Source data are provided in the Source Data file.

a

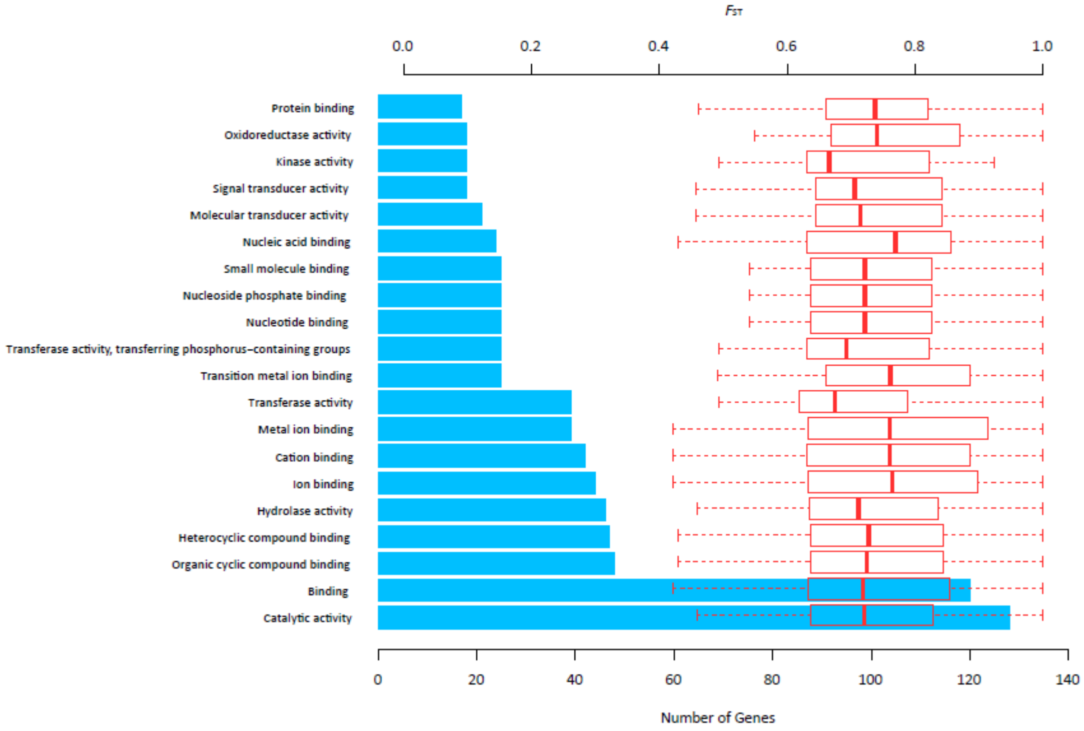

FST
0.0
0.2
0.4
0.6
0.8
1.0
Protein binding
Oxidoreductase activity
Kinase activity
Signal transducer activity
Molecular transducer activity
Nucleic acid binding
Small molecule binding
Nucleoside phosphate binding
Nucleotide binding
Transferase activity, transferring phosphorus–containing groups
Transition metal ion binding
Transferase activity
Metal ion binding
Cation binding
Ion binding
Hydrolase activity
Heterocyclic compound binding
Organic cyclic compound binding
Binding
Catalytic activity
0
20
40
60
80
100
120
140
Number of Genes

b

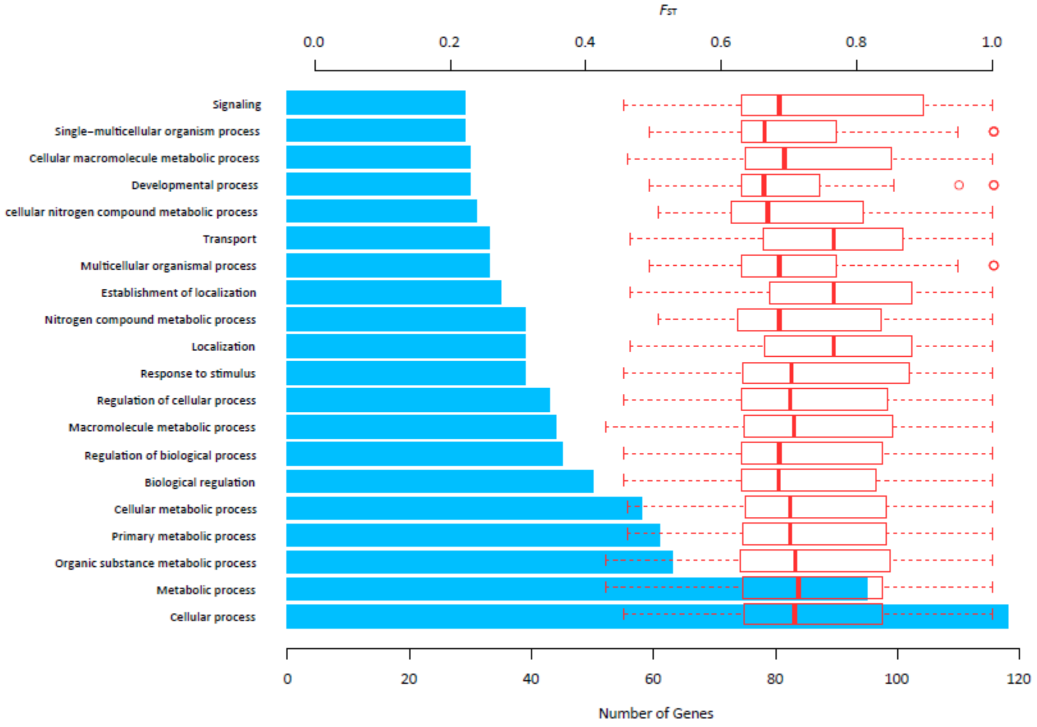

FST
0.0
0.2
0.4
0.6
0.8
1.0
Signaling
Single–multicellular organism process
Cellular macromolecule metabolic process
Developmental process
cellular nitrogen compound metabolic process
Transport
Multicellular organismal process
Establishment of localization
Nitrogen compound metabolic process
Localization
Response to stimulus
Regulation of cellular process
Macromolecule metabolic process
Regulation of biological process
Biological regulation
Cellular metabolic process
Primary metabolic process
Organic substance metabolic process
Metabolic process
Cellular process
0
20
40
60
80
100
120
Number of Genes

c

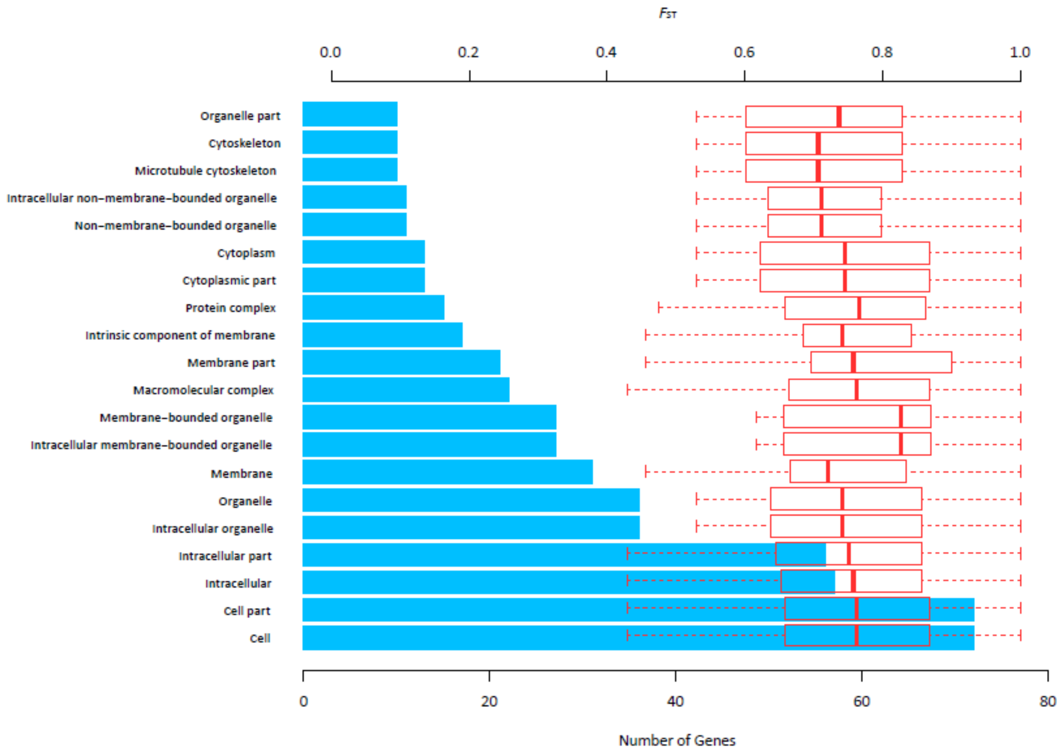

Supplementary Figure 12 Representation of top 20 GO terms of the genes containing significantly differentiated SNPs. The boxplots in the right panel illustrate the range of largest F_{ST} values in every single gene identified by EigenGWAS[10]. **a**, Molecular function; **b**, Biological process; **c**, Cellular component. Boxes show the first and third quartile range (IQR) while whiskers extend to a maximum of 1.5 * IQR. Values for each of the outlier's loci are shown as empty circles. Source data are provided in the Source Data file.

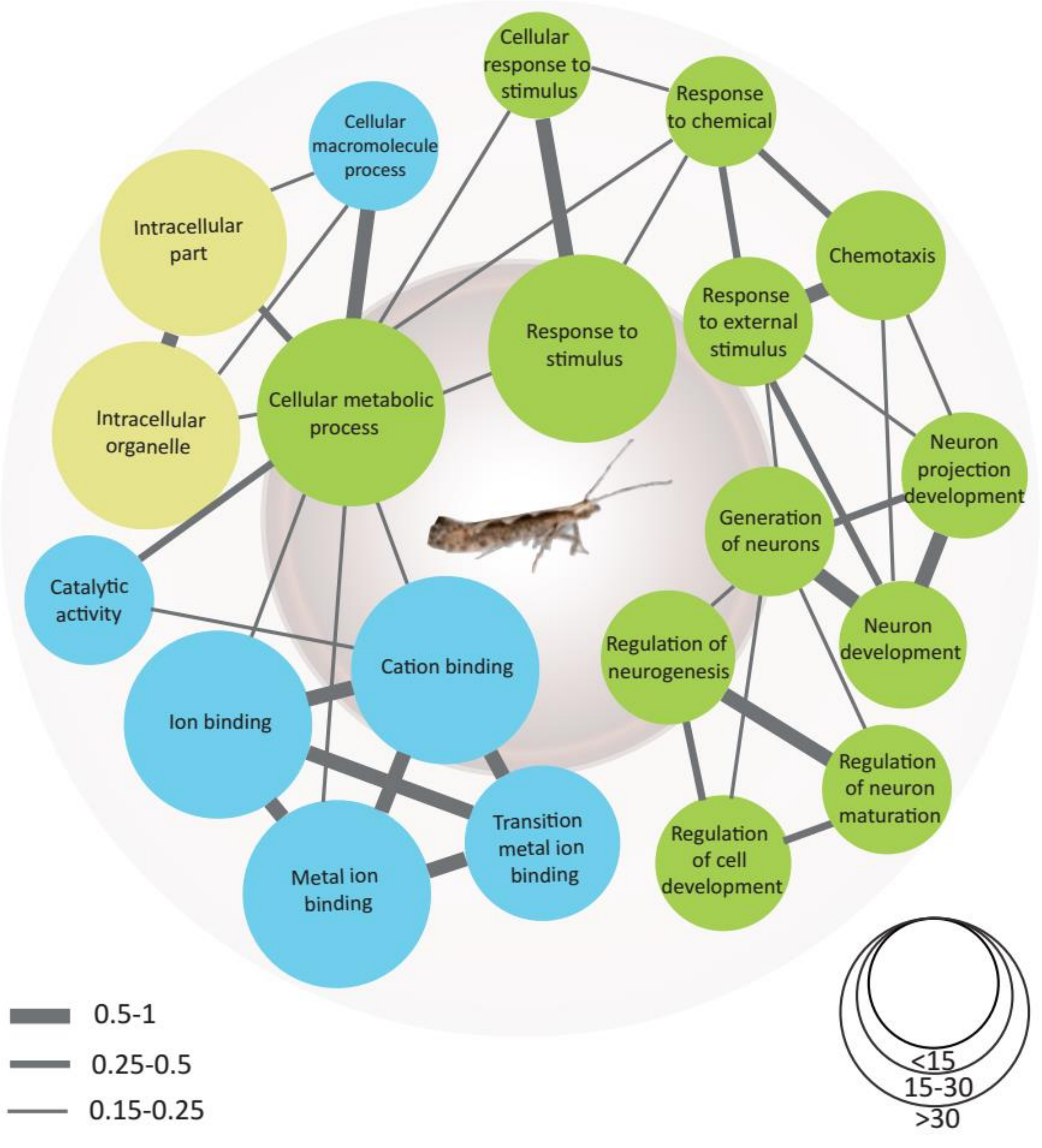

Supplementary Figure 13 GO network of the representative genes in metabolic and signalling pathways. Yellow: cellular terms of GO, Blue: function terms of GO, Green: process terms of GO; thickness of the links indicates Jaccard's index of GO terms based on presence or absence of genes. The moth in this figure was created by Mr. Xin Wang. Source data are provided in the Source Data file.

a

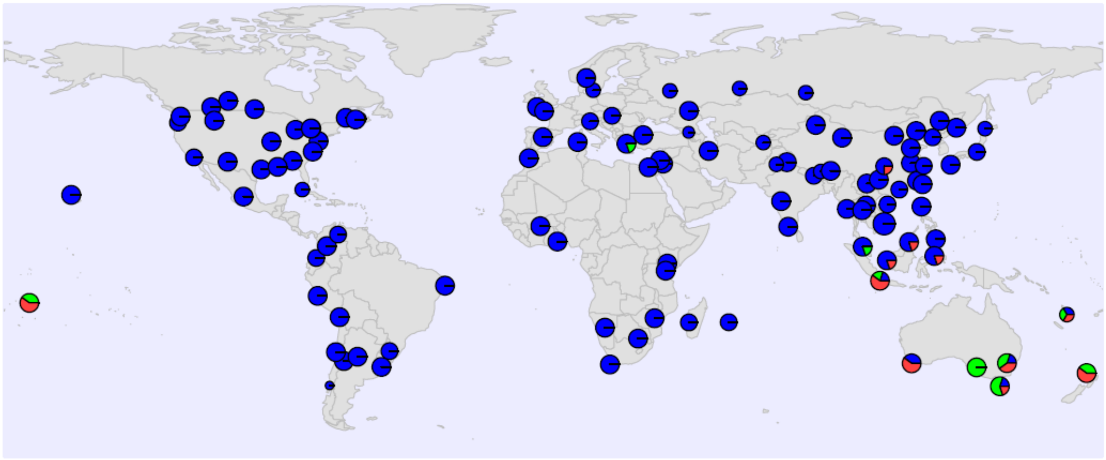

b

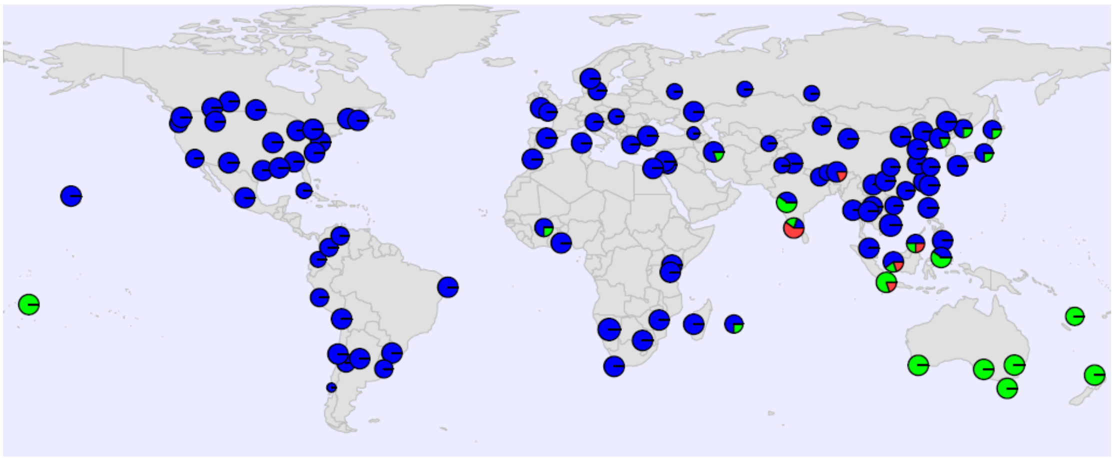

c

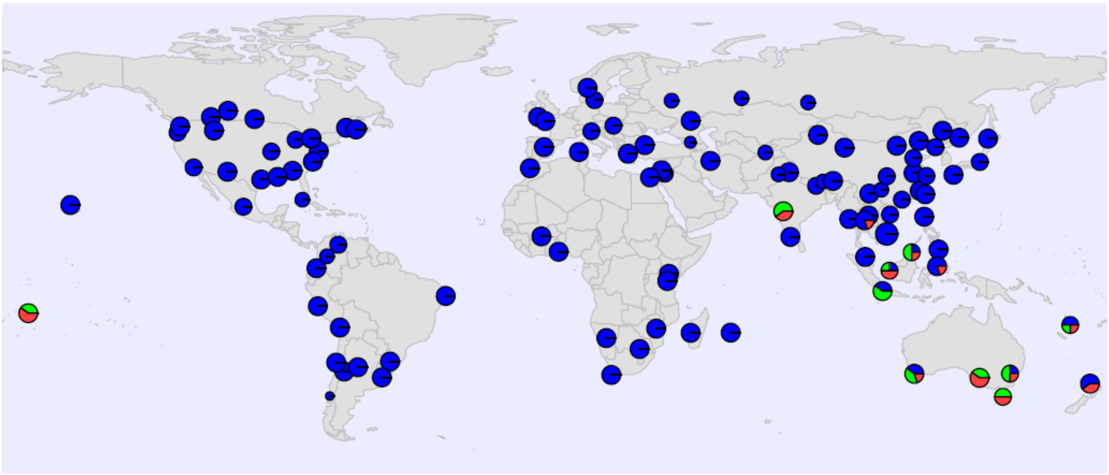

Supplementary Figure 14 Global distribution of the nonsynonymous genotypes of the three genes with point mutations. Blue, green and red colours represent the

original genotype, homozygous mutant and heterozygous mutant, respectively. **a**, PX005867 (F_{ST}= 0.59), **b**, PX002515 (F_{ST}= 1), **c**, PX003348 (F_{ST}= 0.74). Source data are provided in the Source Data file. The maps were generated with the rworldmap package v1.3-6[9].

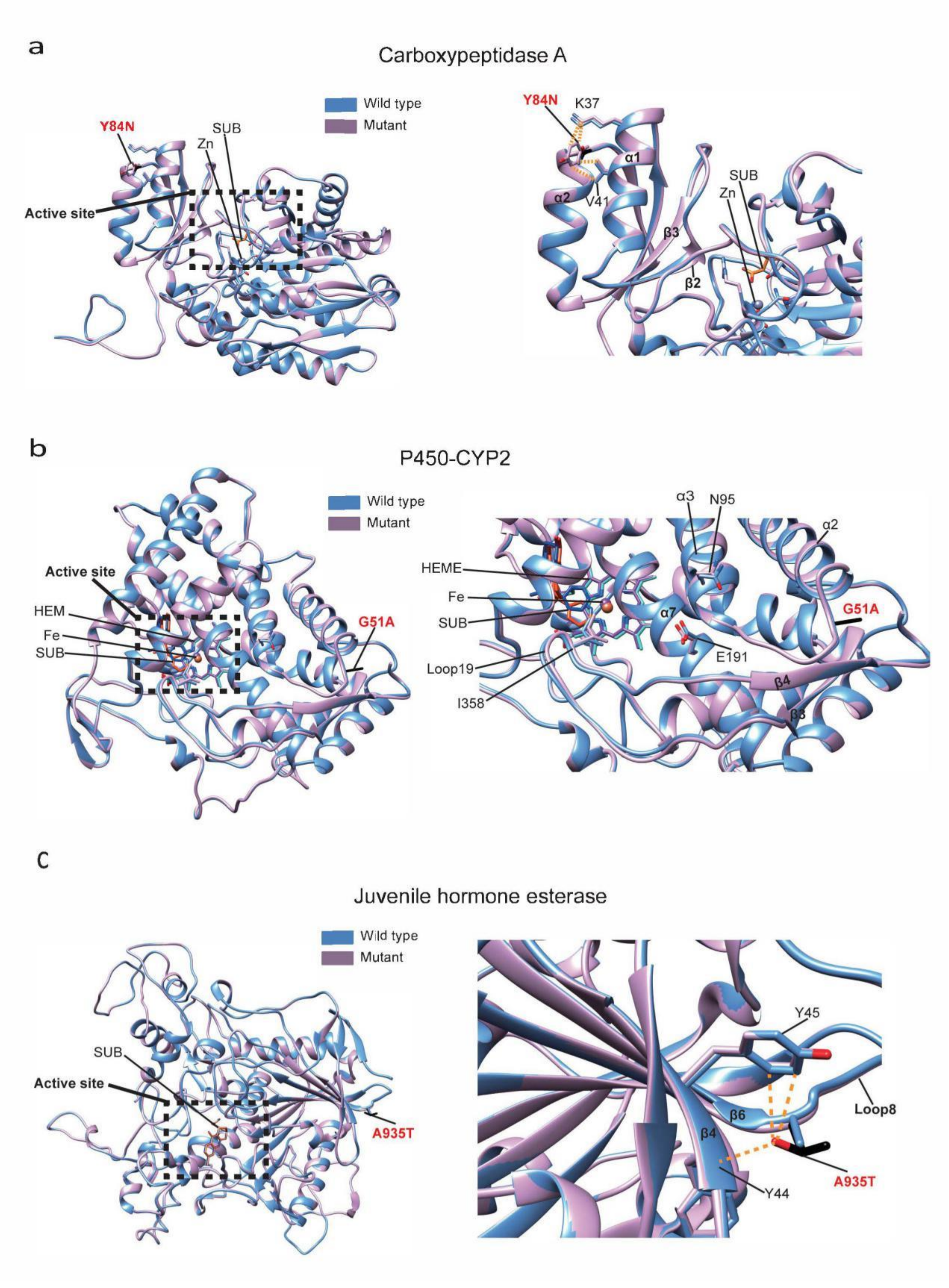

Supplementary Figure 15 Homology models of wild type and mutant enzymes.

Left panels show the structural superpositions of wild type (blue) and mutant (plum) proteins of **a**, Carboxypeptidase A (Px005867), **b**, P450-CYP2 (Px002515) and **c**, Juvenile hormone esterase (JHE, Px003448). Right panels show the zoom-in views of

the mutation sites. The active sites, substrates, key secondary structural elements and residues are labelled. Source data are provided in the Source Data file. **a**, The mutation Y84N is located in a loop following helix α2 of carboxypeptidase A. The side chain of Tyr84 is in contact with Lys37 and Val41. The replacement of the bulky side chain of Tyr with a smaller one of Asn is predicted to reduce the distance between helices α2 and α1 and subsequently move strand β2 and loop β2- β3 towards helix α2, which enlarges the substrate-binding pocket and changes its binding property. **b,** The mutation G51A is located in a loop near the end of helix α2 of P450-CYP2. Gly is known to be a “helix-breaker”, while Ala has the highest helical propensity. So the mutation is predicted to elongate helix α2 and subsequently pull helix α3 towards strand β4. Helix α3 and strand β4 are associated with helix α7 and loop 19, respectively, both of which contribute to the substrate binding. **c,** The mutation A935T is located in a turn after strand β6 of JHE. Compared to the wild type structure, the side chain of T935 forms additional contacts with the side chain of Y45 and the main chain of Y44, which might change the conformation and stability of the local structure.

Supplementary Table 1. Information on the specimens of *P. xylostella* collected in different geographical regions worldwide and *P. australiana*.

Region	Population ID	Location	GPS	Date
Africa	Pop1_AF	Pretoria, South Africa	S25.58, E27.77	August, 2013
	Pop2_AF	Cape Town, South Africa	S34.05, E18.54	August, 2013
	Pop3_AF	Tanzania	S3.37, E36.80	September, 2013
	Pop4_AF	Namibia	S22.01, E16.92	September, 2013
	Pop7_AF	Togo	N6.17, E1.32	September, 2013
	Pop8_AF	Morocco	N33.45, W8.02	September, 2013
	Pop9_AF	Cairo, Egypt	N30.43, E31.18	October, 2013
	Pop11_AF	Bobo-Dioulasso, Burkina Faso	N11.18, W4.28	December, 2013
	Pop12_AF	Manica, Mozambique	S18.93, E33.18	March, 2014
	Pop13_AF	Morondava, Madagascar	S20.29, E44.42	January, 2014
	Pop14_AF	Zewai, Ethiopia	N7.96, E38.72	April, 2014
	Pop16_AF	Mauritius	S20.23, E57.49	January, 2014
	Pop20_AF	Kenya	S01.05, E37.36	September, 2013
Asia	POP1_AS	Dalat, Vietnam	N11.96, E108.42	August, 2013
	POP2_AS	Phetchabun, Thailand	N16.42, E101.19	July, 2013
	POP3_AS	Kota Kinabalu, Malaysia	N05.98, E116.58	July, 2013
	POP4_AS	West Java, Indonesia	S6.66, E107.05	July, 2013
	POP5_AS	North Sulawesi, Indonesia	N1.45, E124.87	July, 2013
	POP7_AS	Kagoshima, Japan	N31.28, E130.20	May, 2013
	POP8_AS	Sapporo, Japan	N43.08, E141.49	September, 2013
	POP9_AS	Yamanashi-ken, Japan	N35.48, E138.80	September, 2013
	POP10_AS	West Kalimantan, Indonesia	S0.00, E109.33	July, 2013
	POP11_AS	Bengued, Philippines	N17.60, E120.65	July, 2013
	POP12_AS	Mount Apo, Philippines	N06.91, E125.35	July, 2013
	POP13_AS	Cameron highland, Malaysia	N04.47, E101.39	November, 2013
	POP14_AS	Vientiane, Laos	N 17.97, E 102.50	November, 2013
	POP15_AS	Rangoon, Myanmar	N16.82, E 96.18	November, 2013
	POP16_AS	Istambul, Turkey	N40.97, E29.46	July, 2013
	POP17_AS	Tbilisi, Georgia	N41.90, E44.34	July, 2013
	POP18_AS	Alborz Province, Iran	N35.81, E50.88	July, 2013
	POP19_AS	Dushanbe, Tajikistan	N38.53, E68.81	September, 2013
	POP20_AS	Katmandu, Nepal	N27.69, E85.37	September, 2013
	POP21_AS	Tamil Nadu, India	N11.02, E76.97	December, 2013
	POP22_AS	Palampur, India	N32.12, E76.53	December, 2013
	POP23_AS	Rahuri, India	N19.38, E74.65	December, 2013
	POP24_AS	Fujian, China	N26.06, E119.21	July, 2012
	POP25_AS	Taiwan, China	N24.91, E121.00	April, 2013
	POP26_AS	Guangdong, China	N23.16, E113.38	October, 2012
	POP27_AS	Hainan, China	N18.31, E109.51	March, 2013

	POP28_AS	Yunnan, China	N25.13, E102.76	November, 2012
	POP29_AS	Guizhou, China	N26.42, E106.68	October, 2012
	POP30_AS	Chongqing, China	N30.79, E108.44	October, 2012
	POP31_AS	An'hui, China	N31.84, E117.19	November, 2012
	POP32_AS	Shanghai, China	N30.90, E121.40	October, 2012
	POP33_AS	Rikaze, Tibet, China	N29.09, E87.65	September, 2013
	POP34_AS	Gongga, Tibet, China	N29.29, E90.89	September, 2013
	POP35_AS	Xinjiang, China	N44.31, E86.00	August, 2013
	POP36_AS	Gansu, China	N40.13, E94.65	August, 2012
	POP37_AS	Hohhot, Inner Mongolia, China	N40.77, E111.67	May, 2012
	POP38_AS	Chifeng, Inner Mongolia, China	N42.31, E118.90	July, 2012
	POP39_AS	Shandong, China	N36.71, E117.13	June, 2013
	POP40_AS	Liaoning, China	N40.25, E124.29	September, 2013
	POP41_AS	Heilongjiang, China	N45.65, E126.61	June, 2012
	POP42_AS	Faisalabad, Pakistan	N31.42, E73.08	April, 2014
	Pop43_AS	Amman, Jordan	N 31.66, E35.97	October, 2013
	Pop44_AS	Israel	N32.76, E34.96	April, 2014
Europe	Pop1_EU	West Cornwall, England	N50.15, W5.37	June, 2013
	Pop2_EU	Madrid, Spain	N40.30, W3.44	August, 2013
	Pop4_EU	Bretagne, France	N48.80, W3.03	July, 2013
	Pop6_EU	Padova, Italy	N45.49, E12.04	July, 2013
	Pop7_EU	Marathon, Greece	N38.15, E23.96	September, 2012
	Pop8_EU	Norway	N59.67, E10.77	July, 2013
	Pop9_EU	Moscow, Russia	N55.47, E38.21	August, 2013
	Pop11_EU	Volgograd, Russia	N48.92, E44.44	August, 2013
	Pop12_EU	Ekaterinburg, Russia	N56.25, E61.04	August, 2013
	Pop13_EU	Novosibirsk, Russia	N54.86, E82.74	August, 2013
	Pop14_EU	Vladivostok, Russia	N43.39, E132.06	August, 2013
	Pop15_EU	Ocsa, Hungary	N47.31, E19.22	August, 2013
	Pop16_EU	Alnarp, Sweden	N55.65, E13.06	August, 2013
North America	Pop1_NA	Prince Edward Island, Canada	N46.39, W63.29	August, 2012
	Pop2_NA	Nova Scotia, Canada	N45.12, W64.44	August, 2012.
	Pop3_NA	Quebec, Canada	N43.14, W79.47	December, 2012
	Pop4_NA	Manitoba, Canada	N49.48, W97.93	July, 2013
	Pop5_NA	Saskatchewan, Canada	N52.15, W106.57	August, 2013
	Pop6_NA	Vauxhaul, Alberta, Canada	N50.09, W112.12	July, 2013
	Pop7_NA	Hawaii, USA	N21.42, W158.02	March, 2013
	Pop8_NA	North Carolina, USA	N35.60, W78.85	July, 2013
	Pop9_NA	Montana, USA	N45.72, W111.15	September, 2013
	Pop10_NA	Maine, USA	N46.64, W68.01	August, 2013
	Pop12_NA	Michigan, USA	N42.70, W84.49	September, 2013
	Pop13_NA	Missouri, USA	N38.85, W92.43	September, 2013
	Pop14_NA	Maryland, USA	N39.01, W76.93	September, 2013

	Pop15_NA	Alabama, USA	N32.57, W85.50	September, 2013
	Pop17_NA	Texas, USA	N29.74, W95.73	September, 2013
	Pop18_NA	Louisiana, USA	N30.60, W90.37；	October, 2013
	Pop19_NA	New Mexico, USA	N32.27, W106.75	October, 2013
	Pop20_NA	Oregon state, USA	N45.07, W123.02	September, 2013
	Pop22_NA	California state, USA	N33.68, W117.78	October, 2013
	Pop25_NA	Vancouver, Canada	N49.25, W123.24	July, 2013
	Pop23_NA	New York, USA	N42.87, W77.08	September, 2013
	Pop24_NA	Seattle, USA	N47.06, W122.19	September, 2013
	Pop26_NA	Ontario, Canada	N43.13, W79.31	March, 2014
	Pop27_NA	New Brunswick, Canada	N46.09, W64.79	March, 2014
	Pop28_NA	Havana, Cuba	N23.16, W82.29	January, 2014
	NA_M	Romita, Mexico	N20.88, W101.54	March, 2014
South America	Pop1_SA	Recife, Brazil	S8.26, W35.51	March, 2013
	Pop2_SA	Santa Maria, Brazil	S29.67, W53.69	March, 2013
	Pop3_SA	Montevideo, Uruguay	S34.84, W56.34	April, 2013
	Pop4_SA	Mendoza, Argentina	S32.92, W68.63	April, 2013
	Pop5_SA	Cordoba, Argentina	S31.52, W64.18	April, 2013
	Pop6_SA	Arica, Chile	S18.57, W70.06	April, 2013
	Pop7_SA	La Serena, Chile	S30.01, W71.25	April, 2013
	Pop8_SA	Osorno, Chile	S40.92, W73.36	April, 2013
	Pop9_SA	Huaral, Peru	S11.61, W77.24	May, 2013
	Pop10_SA	Tulcan, Ecuador	N0.79, W77.70	May, 2013
	Pop11_SA	Bogota, Colombia	N4.69, W74.22	May, 2013
	Pop12_SA	Mucuchies, Venezuela	N8.45, W70.55	October, 2012
Oceania	Pop2_OC	Tasmania, Australia	S41.17, E146.35	September, 2013
	Pop3_OC	Western Australia, Australia	S33.67, E117.44	September, 2013
	Pop4_OC	New South Wales, Australia	S33.61, E148.68	October, 2013
	Pop10_OC	South Australia, Australia	S34.99, E138.71	September, 2013
	Pop5_OC	Upolu, Samoa	S13.89, W171.74	November, 2012
	Pop7_OC	Auckland, New Zealand	S36.87, E174.78	May, 2013
	Pop8_OC	Port Vila, Vanuatu	S17.73, E168.32	July, 2012
	9_Pa10_C (*P. australiana*)	Richmond, New South Wales, Australia	S33.61, E150.76	March, 2014
	PaCook_C (*P. australiana*)	Australian Capital Territory, Australia	S35.35, E149.12	October, 2015

Supplementary **Table 2. Sequencing Statistics**

Sample ID	Sequencing data			Statistics of effective data		
	Raw (Mb)	Clean (Mb)	Mapped (Mb)	Mapped (%)	Coverage (%)	Depth (X)
POP1_AF_4	3308.97	3011.57	2742.88	91.08	75.34	9.68
POP1_AF_5	3643.57	3323.27	2893.81	87.08	75.84	10.15
POP1_AF_6	3316.82	2980.96	2664.28	89.38	75.2	9.42
POP1_AF_7	3519.19	3189.69	2900.41	90.93	75.6	10.2
POP1_AF_8	4040.73	3656.74	3329.84	91.06	76.13	11.63
POP2_AF_2	3845	3464.31	3140.59	90.66	76.41	10.93
POP2_AF_3	3505.72	3095.95	2806.31	90.64	76.03	9.82
POP2_AF_7	3549.05	3233.51	2932.89	90.7	76.45	10.2
POP2_AF_8	4038.91	3506.13	3178.98	90.67	76.65	11.03
POP2_AF_9	3733.91	3419.8	3107.21	90.86	76.55	10.8
POP3_AF_1	5252.27	4645.4	4165.11	89.66	77.86	14.23
POP3_AF_4	2930.32	2562.05	2337.86	91.25	74.03	8.4
POP3_AF_9	5134.93	4484.88	3912.48	87.24	77.56	13.42
POP3_AF_10	3964.2	3572.7	3202.32	89.63	76.7	11.11
POP3_AF_1_2	3365.45	3022.8	2756.43	91.19	75	9.78
POP4_AF_1	6251.9	5545.79	5005.17	90.25	78.41	16.98
POP4_AF_2	2734.55	2402	1994.66	83.04	72.59	7.31
POP4_AF_5	3259.77	2814.35	1794.59	63.77	72.01	6.63
POP4_AF_7	3573.74	3055.73	1909.09	62.48	72.46	7.01
POP4_AF_4_2	2946.72	2542.38	2280.44	89.7	74.01	8.2
POP4_AF_4_3	3229.54	2867.89	2600.6	90.68	75.26	9.19
POP7_AF_1	2804.75	2463.93	2238.67	90.86	74.83	7.96
POP7_AF_6	3107.32	2752.56	2513.11	91.3	75.26	8.88
POP7_AF_7	3064.55	2782.36	2536.38	91.16	75.93	8.88
POP7_AF_8	3593.64	3261.1	2969.82	91.07	76.59	10.31
POP7_AF_9	2914.38	2647.65	2418.74	91.35	75.04	8.57
POP8_AF_2	3396.73	3059.93	2736.24	89.42	75.87	9.59
POP8_AF_3	3053.29	2759.54	2523.59	91.45	75.57	8.88
POP8_AF_7	3353.7	3047.4	2787.48	91.47	76.32	9.71
POP8_AF_9	3274.07	2972.58	2711.46	91.22	76.23	9.46
POP8_AF_10	4110.29	3726.7	3326.65	89.27	77.06	11.48
POP9_AF_2	3742.33	3370.49	3078.76	91.34	76.65	10.68
POP9_AF_3	3703.13	3344.19	2769.03	82.8	76.16	9.67
POP9_AF_4	4084.59	3697.05	3372.6	91.22	77.05	11.64
POP9_AF_7	3801.94	3451.32	3151.98	91.33	77	10.89
POP9_AF_8	3132.47	2775.07	2529.09	91.14	75.72	8.88
POP11_AF_2	2677.49	2396.42	2191.59	91.45	74.7	7.8
POP11_AF_4	3203.5	2917.35	2616.69	89.69	76.14	9.14

POP11_AF_5	4165.08	3776.28	3455.54	91.51	77.01	11.93
POP11_AF_7	2956.94	2369.47	2121.82	89.55	73.75	7.65
POP11_AF_9	4243.82	3751.84	3410.75	90.91	77.07	11.77
POP12_AF_2	2765.13	2524.97	2287.29	90.59	74.89	8.12
POP12_AF_7	2794.5	2546.25	2025.04	79.53	74.1	7.27
POP12_AF_15	3637.95	3288.22	2986.5	90.82	76.4	10.4
POP12_AF_19	3621.21	3294.17	2995.58	90.94	76.47	10.42
POP12_AF_24a	2606.39	2365.4	2144.25	90.65	74.67	7.64
POP13_AF_1	3232.83	2831.63	2565.31	90.59	75.14	9.08
POP13_AF_2	3155.09	2789	2524.95	90.53	75.03	8.95
POP13_AF_4	2923.76	2593.51	2347.48	90.51	74.46	8.38
POP13_AF_6	3064.99	2664.17	2363.5	88.71	74.95	8.39
POP13_AF_7	2279.6	2014.72	1830.36	90.85	72.67	6.7
POP14_AF_3	2933.79	2573.82	2340.98	90.95	74.36	8.37
POP14_AF_6	4034.07	3594.96	3265.65	90.84	76.47	11.36
POP14_AF_8	2643.14	2343.89	2128.99	90.83	73.73	7.68
POP14_AF_9	3653.22	3229.36	2936.99	90.95	75.69	10.32
POP14_AF_10	3061.83	2724.24	2479.38	91.01	74.76	8.82
POP16_AF_1	3401.71	3007.89	2737.43	91.01	75.35	9.66
POP16_AF_4	3408.52	3035.94	2632.22	86.7	74.91	9.35
POP16_AF_5	2702.01	2402.85	2181.67	90.8	74.01	7.84
POP16_AF_6	2576.48	2301.97	2012.76	87.44	73.56	7.28
POP16_AF_7	2608.21	2306.43	2093.69	90.78	73.15	7.61
POP20_AF_1	2982.81	2583.22	2350.43	90.99	74.75	8.36
POP20_AF_3	3159.88	2784.02	2485.42	89.27	74.64	8.86
POP20_AF_4	2711.68	2324.88	2068.08	88.95	73.69	7.46
POP20_AF_5	3308.2	2919.89	2659.06	91.07	75.35	9.39
POP20_AF_9	4364.79	3836.79	3495.22	91.1	76.54	12.15
POP1_AS_2	2587.43	2343.35	2136.62	91.18	74.76	7.6
POP1_AS_3	2799.65	2522.11	2302.52	91.29	74.9	8.18
POP1_AS_7	3090.63	2765.01	2515.62	90.98	75.56	8.85
POP1_AS_9	3072.36	2763.82	2021.8	73.15	74.26	7.24
POP1_AS_4_3	3188.95	2928.84	2678.73	91.46	75.4	9.45
POP1_AS_4_4	2863.59	2606.26	1758.37	67.47	73.36	6.38
POP1_AS_4_5	2213.91	1997.86	1790.69	89.63	73.47	6.48
POP2_AS_1	3190.91	2807.66	2562.36	91.26	75.82	8.99
POP2_AS_4	3351.87	3082.92	2810.85	91.17	76.16	9.82
POP2_AS_5	3850.51	3444.56	3149.44	91.43	77.15	10.86
POP2_AS_11	2948.35	2656.57	1877.81	70.69	71.45	6.99
POP2_AS_36	2675.62	2414.57	1869.44	77.42	71.98	6.91
POP3_AS_1	2589.61	2355.9	2094.45	88.9	74.01	7.53
POP3_AS_2	2525.64	2318.24	2122.11	91.54	74.11	7.62
POP3_AS_3	3711.18	3403.97	3110.27	91.37	76.11	10.87

POP3_AS_5	3210.31	2945.78	2284.86	77.56	74.34	8.17
POP3_AS_2_2	3325.28	3034.43	1921.54	63.32	73.03	7
POP4_AS_1	3637.45	3096.54	2823.4	91.18	76	9.88
POP4_AS_3	5082.38	4654.23	4086.61	87.8	77.56	14.01
POP4_AS_6	4231.02	3720.52	2813.83	75.63	75.87	9.86
POP4_AS_7	2773.02	2338.28	1794.93	76.76	72.89	6.55
POP4_AS_8	3852.22	3308.3	2514.86	76.02	75.15	8.9
POP5_AS_2	4691.79	4215.96	3855.04	91.44	77.23	13.28
POP5_AS_3	2708.67	2411.36	2202.05	91.32	74.53	7.86
POP5_AS_5	3679.05	3343.17	2967.75	88.77	76.02	10.38
POP5_AS_8	3978.84	3587.2	3206.18	89.38	76.28	11.18
POP5_AS_9	3056.17	2737.28	2429.63	88.76	74.94	8.62
POP7_AS_1	3389.78	2999.53	2740.23	91.36	76.03	9.59
POP7_AS_3	3971.42	3555.97	3251.84	91.45	77.19	11.2
POP7_AS_4	3801.12	3352.32	3056.7	91.18	76.85	10.58
POP7_AS_22	2818.97	2570.17	2348.49	91.38	74.83	8.35
POP7_AS_25	3312.39	3018.32	2755.81	91.3	75.76	9.68
POP8_AS_2	3008.68	2606.09	1609.26	61.75	72.26	5.92
POP8_AS_4	4654.5	3864.87	3460.03	89.53	76.38	12.05
POP8_AS_5	5346.02	4763	4325.78	90.82	77.33	14.88
POP8_AS_6	2277.36	2087.24	1887.97	90.45	73.7	6.81
POP8_AS_9	2367.95	2137.06	1926.03	90.13	73.93	6.93
POP9_AS_1	3137.21	2849.28	2571.22	90.24	75.51	9.06
POP9_AS_2	6307.25	5509.12	5036.97	91.43	78.85	16.99
POP9_AS_3	4891.18	4198.38	3850.01	91.7	77.14	13.28
POP9_AS_21	3530.13	3210	2929.57	91.26	76.15	10.23
POP10_AS_2	3397.87	2961.16	2705.18	91.36	76.07	9.46
POP10_AS_4	2755.51	2438.28	2218.2	90.97	73.86	7.99
POP10_AS_10	3137.22	2833.66	2588.87	91.36	75.36	9.14
POP10_AS_2_2	3307.83	2931.83	2676.42	91.29	75.76	9.4
POP10_AS_2_3	2482.39	2184.52	1989	91.05	73.33	7.21
POP11_AS_3	2984.86	2645.77	2133.11	80.62	75.12	7.55
POP11_AS_6	2656.85	2451.81	1575.6	64.26	72.52	5.78
POP11_AS_7	2912.86	2631.83	2366.82	89.93	75.66	8.32
POP11_AS_9	2681.64	2395.58	1966.99	82.11	74.53	7.02
POP11_AS_11	3057.75	2704.7	2457.7	90.87	75.68	8.64
POP12_AS_9	2576.71	2346.98	2143.48	91.33	74.35	7.67
POP12_AS_1a	2560.05	2320.53	2113.89	91.1	73.73	7.63
POP12_AS_3a	2823.68	2546.54	2316.64	90.97	74.11	8.31
POP12_AS_7a	2613.88	2346.48	2138.25	91.13	74.36	7.65
POP12_AS_8a	2883.55	2492.92	2268.71	91.01	74.04	8.15
POP13_AS_1	3621.95	3323.21	3041.15	91.51	76.32	10.6
POP13_AS_2	3611.31	3309.94	3025.59	91.41	76.48	10.52

POP13_AS_6	3816.88	3501.99	3206.53	91.56	76.47	11.15
POP13_AS_7	3851.39	3541.52	3243.97	91.6	76.79	11.24
POP13_AS_10	3410.42	3131.35	2862.57	91.42	75.95	10.02
POP14_AS_2	3949.03	3240.68	2947.42	90.95	76.17	10.29
POP14_AS_3	4343.21	3334.57	3004.69	90.11	75.96	10.52
POP14_AS_5	3941.89	3075.6	2795.8	90.9	75.6	9.84
POP14_AS_6	3598.95	2932.75	2662.52	90.79	75.56	9.37
POP14_AS_13	3528.79	3138.86	2856.48	91	75.63	10.04
POP15_AS_1	2986.01	2468.54	2248.05	91.07	74.38	8.04
POP15_AS_2	2299.19	2101.23	1920.85	91.42	73.46	6.95
POP15_AS_3	2583.12	2333.3	2132.14	91.38	74.42	7.62
POP15_AS_4	5105.67	4279.92	3906.76	91.28	76.99	13.5
POP15_AS_6	3935.96	3342.26	3040.24	90.96	76.25	10.61
POP16_AS_2	2580.04	2346.05	1548.95	66.02	72.22	5.7
POP16_AS_4	3642.97	3085.58	2810.48	91.08	75.58	9.89
POP16_AS_5	6047.72	5155.09	4544.22	88.15	78.44	15.41
POP16_AS_6	2797.95	2532.36	2258.79	89.2	74.73	8.04
POP16_AS_5_2	2488.36	2247.72	1461.19	65.01	71.46	5.44
POP17_AS_1	4005.02	3540.57	3220.38	90.96	76.88	11.14
POP17_AS_1_2	3112.95	2802.83	2524.2	90.06	75.22	8.93
POP18_AS_1	2634.39	2390.68	2181.86	91.27	74.76	7.76
POP18_AS_5	2558.93	2300.67	1983.72	86.22	74.48	7.08
POP18_AS_6	3969.64	3472.29	3022.16	87.04	76.45	10.51
POP18_AS_7	3264.98	2859.03	2594.86	90.76	75.34	9.16
POP18_AS_11	4038.21	3559.33	2923.08	82.12	76.09	10.22
POP19_AS_3	3343.4	2865.59	2440.2	85.16	75.23	8.63
POP19_AS_5	3555.43	2829.38	2571.2	90.87	75.27	9.09
POP19_AS_6	3951.86	3249.31	2959.58	91.08	75.9	10.37
POP20_AS_2	2847.6	2584.4	2358.4	91.26	74.87	8.38
POP20_AS_3	3855.97	3466.69	3159.94	91.15	76.38	11
POP20_AS_4	3622.8	3282.41	2959.91	90.18	76.31	10.32
POP20_AS_5	2642.44	2355.67	2139.01	90.8	74.36	7.65
POP21_AS_2	3639.44	3163.19	2868.28	90.68	76.68	9.95
POP21_AS_3	3570.36	3080.56	2810.74	91.24	75.18	9.94
POP21_AS_4	3703.8	3333.09	3036.03	91.09	76.67	10.53
POP21_AS_5	3683.43	3328.33	3031.31	91.08	76.77	10.5
POP21_AS_6	3283.19	2950.94	2682.75	90.91	75.01	9.51
POP22_AS_1	2930.11	2618.41	2392.56	91.37	74.56	8.53
POP22_AS_2	3740.04	3396.5	3103.56	91.38	75.87	10.88
POP22_AS_6	3542.85	3127.13	2851.79	91.2	75.85	10
POP22_AS_7	3205.32	2855.65	2609.19	91.37	75.3	9.22
POP22_AS_8	4102.35	3665.95	3332.11	90.89	76.52	11.58
POP23_AS_2	3335.31	2830.69	2560.28	90.45	74.64	9.12

POP23_AS_5	3298.05	3007.61	2739.92	91.1	74.85	9.74
POP23_AS_6	4806.01	4297.95	3854.72	89.69	76.47	13.41
POP23_AS_13	3020.69	2751.37	2507.19	91.12	73.81	9.03
POP23_AS_2_2	3050.76	2768.12	2525.25	91.23	73.92	9.09
POP24_AS_1	5193.66	4261.45	3877.87	91	77.37	13.33
POP24_AS_2	3229.01	2892.6	2635.78	91.12	76.26	9.19
POP24_AS_3	3729.57	3369.51	3071.95	91.17	76.68	10.66
POP24_AS_4	3219.78	2870.86	2617.51	91.17	76.11	9.15
POP24_AS_13	3303.58	3019.24	2751.02	91.12	76.08	9.62
POP25_AS_1	2405.78	2148.25	1953.25	90.92	72.92	7.12
POP25_AS_2	3138.84	2874.91	1870.08	65.05	74	6.72
POP25_AS_3	3188.2	2791.25	2542.47	91.09	74.96	9.02
POP25_AS_4	3972.92	3575.43	2692.87	75.32	75.43	9.5
POP25_AS_5	3505.67	3166.09	2817.02	88.97	75.6	9.91
POP26_AS_3	3691.92	3062.76	2786.64	90.98	75.91	9.76
POP26_AS_4	4326.81	3973.62	3629.51	91.34	77.74	12.42
POP26_AS_5	3646.74	3153.04	2854.68	90.54	76.38	9.94
POP26_AS_6	4711.5	4277.37	3908.15	91.37	77.83	13.36
POP27_AS_1	2980.12	2712.54	2477.93	91.35	75.2	8.76
POP27_AS_3	3505.07	3099.67	2831.57	91.35	76.36	9.86
POP27_AS_5	3713.27	3262.39	2980.66	91.36	76.34	10.38
POP27_AS_6	2300.21	2028.42	1841.29	90.77	72.31	6.77
POP28_AS_1	4090.38	3595.31	3262.26	90.74	77.19	11.24
POP28_AS_2	4191.39	3553.82	3100.26	87.24	76.66	10.76
POP28_AS_3	3723.34	3192.72	2790.12	87.39	76.52	9.7
POP28_AS_4	4121.22	3680.96	3326.76	90.38	77.31	11.45
POP28_AS_5	4087.9	3578.44	3258.69	91.06	77.02	11.25
POP29_AS_1	3104.89	2545.05	2180.76	85.69	74.39	7.8
POP29_AS_2	3995.15	3543.84	3206.82	90.49	77.48	11.01
POP29_AS_3	4795.56	4250.75	3543.96	83.37	77.71	12.13
POP29_AS_4	3823.12	3329.3	2806.99	84.31	76.68	9.74
POP29_AS_5	4190.53	3727.74	3021.55	81.06	77.12	10.42
POP30_AS_1	4570.94	4047.96	3636.11	89.83	77.84	12.42
POP30_AS_3	4544.48	3990.39	3645.81	91.36	77.48	12.52
POP30_AS_5	4033.19	3518.03	3169.86	90.1	76.77	10.98
POP30_AS_6	3588.19	3141.38	2851.72	90.78	76.09	9.97
POP31_AS_1	2544.07	2339.38	2131.11	91.1	74.2	7.64
POP31_AS_3	2594.78	2384.35	2170.38	91.03	74.26	7.77
POP31_AS_5	3023.8	2779.23	2490.47	89.61	75.3	8.8
POP31_AS_7	3291.56	2986.3	2724.23	91.22	75.69	9.57
POP31_AS_8	3495.87	3142.58	2816.83	89.63	76.08	9.85
POP32_AS_1	3656.57	3253.26	2916.15	89.64	76.74	10.11
POP32_AS_3	4022.54	3574.73	3261.59	91.24	77.08	11.25

POP32_AS_4	4818.77	4402.78	4003.53	90.93	78.16	13.62
POP32_AS_5	3727.92	3361.11	3069.16	91.31	76.69	10.64
POP33_AS_2	2824.8	2552.13	2315.48	90.73	75.34	8.17
POP33_AS_4	2581.1	2245.21	1588.19	70.74	63.09	6.7
POP33_AS_6	4024.66	2460.29	2222.82	90.35	72.97	8.1
POP34_AS_1	3652.28	3158.38	2876.35	91.07	76.37	10.02
POP34_AS_3	3895.96	3490	3184.23	91.24	77.24	10.96
POP34_AS_5	4543.29	4022	3658.66	90.97	77.72	12.52
POP34_AS_6	3701.94	2977.77	2712.52	91.09	75.51	9.55
POP34_AS_7	3422.6	2898.48	2638.53	91.03	75.56	9.29
POP35_AS_1	2386.15	2108.74	1919.23	91.01	73.6	6.94
POP35_AS_2	3136.31	2781.33	2511.45	90.3	75.49	8.85
POP35_AS_3	2318.78	2125.59	1942.43	91.38	73.9	6.99
POP35_AS_5	4256.91	3404.67	3106.51	91.24	76.12	10.85
POP35_AS_6	2985.38	2636.55	2402.55	91.12	75.39	8.48
POP36_AS_1	3374.53	3028.74	2747.15	90.7	76.1	9.6
POP36_AS_2	4116.11	3399.79	3044.34	89.54	76.52	10.58
POP36_AS_3	3736.8	3335.29	3038.02	91.09	76.43	10.57
POP36_AS_4	3171.62	2851.24	2594.37	90.99	75.65	9.12
POP36_AS_5	2610.07	2306.13	2093.98	90.8	74.73	7.45
POP37_AS_1	3786.71	3297.55	3007.09	91.19	76.46	10.46
POP37_AS_2	1908.35	1760.85	1614.21	91.67	71.54	6
POP37_AS_3	3086.6	2750.09	2503.96	91.05	75.58	8.81
POP37_AS_4	3892.4	3447.73	3139.27	91.05	76.53	10.91
POP37_AS_6	1739.33	1592.12	1462.1	91.83	70.08	5.55
POP38_AS_1	3065.15	2746.76	2503.8	91.15	75.48	8.82
POP38_AS_2	3434.99	2850.61	2595.57	91.05	75.29	9.17
POP38_AS_4	4149.95	3676.47	3357.6	91.33	77.1	11.58
POP38_AS_5	3249.31	2799.13	2545.47	90.94	75.32	8.99
POP38_AS_6	4203.99	3750.8	3266.24	87.08	77.32	11.23
POP39_AS_1	2965.68	2678.66	2360.29	88.11	75.17	8.35
POP39_AS_4	2105.9	1902.69	1738.12	91.35	73.14	6.32
POP39_AS_7	2047.53	1874.48	1414.59	75.47	71	5.3
POP39_AS_8	2580.88	2351.68	1991.61	84.69	73.82	7.18
POP39_AS_4_2	3769.29	3398.33	2664.47	78.41	75.72	9.36
POP40_AS_2	2591.99	2353.78	2077.19	88.25	73.75	7.49
POP40_AS_3	1891.78	1730.26	1583.49	91.52	71.08	5.93
POP40_AS_4	3433.35	3011.5	2745.71	91.17	75.93	9.62
POP40_AS_5	3297.78	2899.89	2640.75	91.06	75.76	9.27
POP40_AS_3_2	2798.52	2503.99	2125.1	84.87	73.77	7.66
POP41_AS_1	3506.9	2874.1	2622.24	91.24	74.53	9.36
POP41_AS_2	3611.24	3168.39	2848.83	89.91	75.1	10.09
POP41_AS_3	3533.24	3139.68	2900.64	92.39	76.22	10.12

POP41_AS_4	3880.55	3512.66	3250.23	92.53	76.06	11.36
POP41_AS_5	3542.93	3168.29	2890.88	91.24	74.95	10.26
POP42_AS_3	3671.08	3266.46	2970.89	90.95	76	10.4
POP42_AS_5	3204.27	2876.59	2609.83	90.73	75.67	9.17
POP42_AS_7	3763.42	3414.64	3115.56	91.24	76.25	10.87
POP43_AS_3	3256.81	2918.54	2664.68	91.3	75.61	9.37
POP43_AS_4	3273.4	2902.61	2630.86	90.64	75.66	9.25
POP43_AS_5	3081.99	2736.72	2499.09	91.32	75.32	8.82
POP43_AS_7	3190.8	2872.84	2626.11	91.41	75.59	9.24
POP43_AS_8	3388	3043.31	2779.74	91.34	76	9.73
POP44_AS_2	3047.57	2699.45	2455.13	90.95	74.95	8.71
POP44_AS_3	3016.44	2682.92	2442.45	91.04	75.02	8.66
POP44_AS_5	3528.4	3118.85	2831.38	90.78	75.8	9.93
POP44_AS_6	3219.63	2832.41	2587.36	91.35	75.41	9.13
POP44_AS_10	2557.59	2277.05	2071.3	90.96	73.54	7.49
POP1_EU_1	3688.35	3397.9	3102.81	91.32	76.86	10.74
POP1_EU_4	3844.43	3425.55	3129.55	91.36	76.67	10.86
POP1_EU_7	3300.18	3032.86	2775.39	91.51	76.06	9.7
POP1_EU_4_2	3472	3173.06	2878.62	90.72	76.09	10.06
POP1_EU_7_2	3469.7	3162.33	2888.22	91.33	76.18	10.08
POP2_EU_3	3838.05	3490.79	3180.16	91.1	75.78	11.16
POP2_EU_21	3220.97	2947.58	2687.22	91.17	75.54	9.46
POP2_EU_22	2926.14	2655.11	2419.77	91.14	74.69	8.62
POP2_EU_24	3535.32	3222.39	2935.41	91.09	76	10.27
POP2_EU_26	3167.03	2887.83	2623.18	90.84	75.86	9.2
POP4_EU_1	3526.95	3200.85	2759.2	86.2	76.65	9.57
POP4_EU_3	3264.88	2987.61	2727.7	91.3	75.85	9.56
POP4_EU_4	3083.77	2809.55	2500.83	89.01	75.38	8.82
POP4_EU_5	2445.84	2197.2	2005.51	91.28	74.18	7.19
POP4_EU_6	3885.77	3563.12	3250.26	91.22	77.02	11.22
POP6_EU_2	3056.83	2659.91	2334.33	87.76	74.68	8.31
POP6_EU_3	3454.41	3142.85	2871.45	91.36	76.41	10
POP6_EU_4	4103.21	3737.63	3405.57	91.12	77.33	11.71
POP6_EU_7	3538.84	3229.5	2949.67	91.34	76.57	10.25
POP7_EU_3	3150.07	2602.38	2348.83	90.26	74.69	8.36
POP7_EU_4	3220.71	2780.96	2528.66	90.93	75.43	8.92
POP7_EU_6	3740.02	3397.32	3102.28	91.32	76.63	10.77
POP7_EU_8	4549.29	3987.26	3633.05	91.12	77.2	12.52
POP7_EU_9	3741.45	3466.64	3155.5	91.02	77	10.9
POP8_EU_1	3281.91	2977.63	2716.64	91.24	76.17	9.49
POP8_EU_2	3200.25	2920.01	2665.73	91.29	75.79	9.36
POP8_EU_3	3072.44	2605.26	2359.47	90.57	74.22	8.46
POP8_EU_5	2817.45	2533.55	2309.9	91.17	75.19	8.17

POP8_EU_8	3479.54	3129.07	2847.9	91.01	76.52	9.9
POP9_EU_2	3722.04	3381.1	3077.7	91.03	76.71	10.67
POP9_EU_6	3487.66	3170.96	2890.41	91.15	76.67	10.03
POP9_EU_8	3486.43	3187.21	2908.42	91.25	77.55	9.97
POP11_EU_1	3017.57	2704.75	2082.74	77	74.38	7.45
POP11_EU_2	3305.97	2995.13	2733.37	91.26	75.97	9.57
POP11_EU_7	3153.38	2842.25	2593.58	91.25	76.01	9.08
POP11_EU_9	3872.98	3538.98	3236.38	91.45	77.04	11.17
POP11_EU_7_2	3186.61	2887.05	2635.58	91.29	75.68	9.26
POP12_EU_2	2413.26	2142.52	1926.76	89.93	73.74	6.95
POP12_EU_8	3518.92	3214.63	2937.1	91.37	76.17	10.26
POP12_EU_10	3095.35	2830.92	2586.7	91.37	75.55	9.11
POP13_EU_2	2797.64	2556.06	2330.39	91.17	75.17	8.25
POP13_EU_5	3448.25	3123.49	2852.98	91.34	75.98	9.99
POP13_EU_6	3264.23	2953.73	2698.76	91.37	75.74	9.48
POP14_EU_2	3825.16	3495.38	3186.14	91.15	76.99	11.01
POP14_EU_3	5125.57	4481.21	4038.54	90.12	77.71	13.82
POP14_EU_4	4157.05	3803.82	3474.35	91.34	77.46	11.93
POP14_EU_9	4038.78	3678.75	3291.26	89.47	77.36	11.31
POP14_EU_2_2	2842.05	2562.24	2135.97	83.36	74.87	7.59
POP15_EU_2	3187.31	2704.37	2457.83	90.88	75	8.72
POP15_EU_5	4504.64	4000.03	3654.73	91.37	77.43	12.55
POP15_EU_6	3694.21	3340.92	3024.02	90.51	76.48	10.52
POP15_EU_7	3518.6	3173.95	2891.86	91.11	76.23	10.09
POP16_EU_1	2780.4	2397.24	2181.08	90.98	74.7	7.77
POP16_EU_2	2303.13	2124.85	1932.67	90.96	73.22	7.02
POP16_EU_9	3044.94	2406.06	2183.06	90.73	73.8	7.87
POP16_EU_31	3966.24	3559.37	3242.62	91.1	76.47	11.28
POP1_NA_4	3490.41	3054.94	2602.4	85.19	75.77	9.14
POP1_NA_22	3597.05	3239.11	2935.57	90.63	75.96	10.28
POP2_NA_26	3370.47	3051.66	2771.62	90.82	75.53	9.76
POP3_NA_1	3537.06	3211.62	2923.66	91.03	74.32	10.46
POP3_NA_3	3202.59	2912.84	2647.73	90.9	73.81	9.54
POP3_NA_4	3624.16	3249.72	2958.21	91.03	74.84	10.51
POP3_NA_6	4233.12	3803.13	3461.93	91.03	75.75	12.16
POP3_NA_7	4103.95	3667.42	3335.37	90.95	75.52	11.75
POP4_NA_1	4070.24	3563.96	3200.77	89.81	76.87	11.08
POP4_NA_5	3481.34	3172.11	2876.5	90.68	76.15	10.05
POP4_NA_6	3060.98	2772.59	2513.23	90.65	75.38	8.87
POP4_NA_21	3007.41	2736.7	2459.44	89.87	74.59	8.77
POP4_NA_22	2808.21	2557.26	2315.08	90.53	74.34	8.28
POP5_NA_4	2739.39	2527.4	2296.22	90.85	74.84	8.16
POP5_NA_5	2627.38	2423.83	2207.36	91.07	74.78	7.85

POP5_NA_6	5302.72	4571.8	4160.93	91.01	77.88	14.21
POP5_NA_7	2884.72	2647.21	2404.95	90.85	75.29	8.5
POP5_NA_8	2925.3	2694.16	2449.91	90.93	75.47	8.63
POP6_NA_1	3638.41	3340.66	2404.94	71.99	74.94	8.54
POP6_NA_5	3099.11	2833.72	2571.82	90.76	75.57	9.05
POP6_NA_6	3334.22	3062.14	2734.16	89.29	75.74	9.6
POP6_NA_11	4145.08	3817.19	3346.08	87.66	76.92	11.57
POP6_NA_12	3125.64	2703.48	2454.75	90.8	74.98	8.71
POP7_NA_1	4177.21	3734.83	3392.33	90.83	76.89	11.74
POP7_NA_3	4384.34	3961.22	3498.23	88.31	77.43	12.02
POP7_NA_7	2552.63	2282.16	2024.34	88.7	74.31	7.25
POP7_NA_21	2788.83	2529.09	2288.9	90.5	74.68	8.15
POP7_NA_1_2	3052.32	2776.55	2532.56	91.21	75.04	8.98
POP8_NA_2	2892.12	2613.1	2382.05	91.16	75.27	8.42
POP8_NA_3	3576.72	3240.63	2959.22	91.32	76.79	10.25
POP8_NA_4	3943.13	3526.57	3055.5	86.64	76.53	10.62
POP8_NA_7	3620.45	3241	2176.51	67.16	74.68	7.75
POP8_NA_11	4334.14	3888.5	3535.35	90.92	77.26	12.17
POP9_NA_3	3535.51	3197	2880.04	90.09	76.37	10.03
POP9_NA_6	3310.8	2936.34	2660.15	90.59	75.66	9.35
POP9_NA_9	3085.77	2684.09	2434.74	90.71	74.75	8.66
POP9_NA_10	4276.54	3860.62	3444.73	89.23	77.17	11.87
POP9_NA_11	3301.3	2956.71	2684.77	90.8	75.79	9.42
POP10_NA_1	3995.24	3557.8	3166.15	88.99	76.34	11.03
POP10_NA_8	4108.64	3704.83	3296.92	88.99	77.09	11.37
POP10_NA_9	4104.03	3669.35	3265.55	89	77	11.28
POP10_NA_10	3579.56	3249.72	2913.69	89.66	76.71	10.1
POP10_NA_12	4711.49	4167.79	3793.05	91.01	76.97	13.11
POP12_NA_1	4005.56	3598.89	3247.6	90.24	76.39	11.31
POP12_NA_2	2304.65	2031.68	1811.21	89.15	72.5	6.64
POP12_NA_3	2865.37	2527.12	2168.23	85.8	74.05	7.79
POP12_NA_4	3639.56	3235.22	2935.6	90.74	75.88	10.29
POP12_NA_5	3196.08	2838.52	2543.45	89.6	74.76	9.05
POP13_NA_1	3811.67	3423.28	3086.86	90.17	76.51	10.73
POP13_NA_2	3384.14	3061.11	2777.11	90.72	75.99	9.72
POP13_NA_5	3182.76	2841.19	2568.67	90.41	75.36	9.07
POP13_NA_6	3943.16	3589.32	3256.44	90.73	76.6	11.31
POP13_NA_7	3061.14	2719.42	2468.4	90.77	75.43	8.7
POP14_NA_1	3723.97	3283.46	2980.42	90.77	76.46	10.37
POP14_NA_2	4033.38	3570.56	2920.87	81.8	76.25	10.19
POP14_NA_12	4180.48	3789.53	3456.34	91.21	77.11	11.92
POP14_NA_13	3070.03	2723.31	2394.9	87.94	74.68	8.53
POP14_NA_15	3227.45	2819.07	2554.94	90.63	75.92	8.95

POP15_NA_2	3353.5	2983.3	2704.81	90.66	75.83	9.49
POP15_NA_3	4652.19	4150.3	3760.13	90.6	77.32	12.93
POP15_NA_4	3751.86	3364.03	3054.73	90.81	76.76	10.58
POP15_NA_5	2528.56	2264.61	1986.73	87.73	73.92	7.15
POP15_NA_6	4431.3	3930.32	3459.64	88.02	77.11	11.93
POP17_NA_1	3928.91	3456.7	3131.4	90.59	76.44	10.9
POP17_NA_3	4295.99	3821.57	3451.81	90.32	77.18	11.9
POP17_NA_4	4245.8	3788.69	3441.07	90.82	77.34	11.83
POP17_NA_5	4319.7	3901.09	3528.53	90.45	77.17	12.16
POP17_NA_6	5145.9	4609.55	4011.39	87.02	77.74	13.72
POP18_NA_1	2901.19	2644.3	2372.84	89.73	74.86	8.43
POP18_NA_4	2662.99	2409.89	2098.09	87.06	74.08	7.53
POP18_NA_21	2982.68	2693.42	2442.7	90.69	75.03	8.66
POP18_NA_22	3232.77	2910.12	2627.06	90.27	75.42	9.26
POP18_NA_23	4080.59	3673.63	3337.73	90.86	76.57	11.59
POP19_NA_1	3973.57	3579.4	3251.01	90.83	76.98	11.23
POP19_NA_5	3309.85	2978.94	2704.81	90.8	75.84	9.49
POP19_NA_9	4791.45	4356.82	3962.99	90.96	77.54	13.59
POP19_NA_10	4079.51	3664.35	3334.74	91.01	77.05	11.51
POP19_NA_11	3589.92	3225.17	2547.18	78.98	75.52	8.97
POP20_NA_1	2607.55	2364.64	2149.25	90.89	74.19	7.7
POP20_NA_3	2714.8	2481.78	2254.57	90.84	74.36	8.06
POP20_NA_5	2967.15	2716.13	2470.13	90.94	75.02	8.76
POP20_NA_25	3362.34	3059.5	2779.19	90.84	75.49	9.79
POP22_NA_3	4073.38	3618.85	3267.2	90.28	76.85	11.31
POP22_NA_4	3727.12	3298.38	3009.75	91.25	76.16	10.51
POP22_NA_10	3711.28	3356.34	3058.04	91.11	76.61	10.62
POP22_NA_12	3566.34	3241.59	2943.86	90.82	76.19	10.28
POP23_NA_1	2910.6	2566.16	1901.47	74.1	73.87	6.85
POP23_NA_3	2727.96	2411.11	2181.59	90.48	74.73	7.76
POP23_NA_4	3654.73	3207.99	2686.66	83.75	75.94	9.41
POP23_NA_10	3287.24	2985.88	2709.87	90.76	75.61	9.53
POP23_NA_6_2	2798.87	2485.23	2260.33	90.95	73.74	8.15
POP24_NA_1	3496.34	2972.42	2687.06	90.4	75.38	9.48
POP24_NA_2	4735.12	4216.32	3482.06	82.59	76.76	12.07
POP24_NA_3	4546.8	3966.03	3578.05	90.22	76.99	12.36
POP24_NA_5	4121.58	3719.26	3229.49	86.83	76.91	11.17
POP24_NA_10	4447.53	3967.66	3601.27	90.77	77.32	12.39
POP25_NA_1	2505.26	2251.99	2044.62	90.79	73.88	7.36
POP25_NA_5	2826.86	2428.33	2200.27	90.61	74.25	7.88
POP25_NA_6	3381.68	3014.57	2724.32	90.37	75.62	9.58
POP25_NA_8	2128.52	1948.52	1765.74	90.62	73.06	6.43
POP25_NA_10	3823.96	3329.37	2909.24	87.38	75.96	10.19

POP26_NA_1	3189.87	2846.98	2583.27	90.74	74.86	9.18
POP26_NA_2	2616.59	2304.46	2094.55	90.89	73.1	7.62
POP26_NA_3	2319.77	2026.13	1833.92	90.51	72.88	6.69
POP26_NA_4	2676.74	2371.89	2155.37	90.87	73.81	7.77
POP26_NA_5	3372.6	3055.47	2778.6	90.94	75.52	9.79
POP27_NA_2	2664.53	2430.06	2212.56	91.05	74.4	7.91
POP27_NA_3	3890.53	3519.5	3196.73	90.83	76.37	11.13
POP27_NA_4	3008.24	2753.42	2502.81	90.9	74.26	8.96
POP27_NA_5	3013.99	2774.26	2516.51	90.71	74.94	8.93
POP27_NA_6	3028.87	2786.34	2527.69	90.72	75.3	8.93
POP28_NA_2	2723.25	2434.12	2218.45	91.14	74.3	7.94
POP28_NA_4	2743.85	2399.84	2186.19	91.1	73.93	7.87
POP28_NA_8	3361.32	2988.35	2724.96	91.19	75.79	9.56
NA_M_5	3520.77	2976.9	2715.97	91.23	75.73	9.54
NA_M_6	3578.51	3018.14	2753.46	91.23	75.75	9.67
NA_M_8	3368.25	2828.61	2579.4	91.19	75.4	9.1
NA_M_11	5747.18	5130.76	4702.46	91.65	77.54	16.13
NA_M_12	4396.44	3659.59	3335.6	91.15	76.7	11.57
POP1_SA_1	3441.99	3156.06	2878.53	91.21	75.41	10.15
POP1_SA_4	3542.48	3244.08	2865.39	88.33	75.2	10.13
POP1_SA_6	6228.37	5759.68	5093.18	88.43	77.8	17.41
POP1_SA_7	3719.34	3394.21	3032.42	89.34	75.51	10.68
POP1_SA_9	3938.9	3591.67	3283.83	91.43	75.89	11.51
POP2_SA_2	3189.87	2902.56	2629.76	90.6	74.82	9.35
POP2_SA_7	3307.4	3002.27	2725.17	90.77	74.36	9.75
POP2_SA_12	3905.55	3561.65	3243.18	91.06	76.14	11.33
POP2_SA_24	3594.17	3316.2	3004.4	90.6	75.8	10.54
POP2_SA_25	3249.56	2955.01	2663.32	90.13	74.91	9.46
POP3_SA_3	4385.39	3955.74	3595.06	90.88	76.67	12.47
POP3_SA_4	4002.25	3663.42	3270.07	89.26	76.31	11.4
POP3_SA_5	4071.52	3655.21	3315.38	90.7	76.43	11.54
POP3_SA_7	4325.49	3904.89	3400.28	87.08	76.53	11.82
POP3_SA_8	3986.44	3569.51	3241.05	90.8	76.39	11.29
POP4_SA_11	3052.3	2759.96	2509.23	90.92	74.72	8.93
POP4_SA_29	3573.22	3253.09	2955.19	90.84	75.63	10.39
POP4_SA_37	3816.53	3468.27	3133.27	90.34	75.8	10.99
POP4_SA_39	3348.27	3035.21	2757.44	90.85	75.24	9.75
POP4_SA_42	2679.98	2454.61	2231.7	90.92	74.17	8
POP5_SA_3	2911.04	2659.04	2419.23	90.98	74.57	8.63
POP5_SA_5	4110.16	3776.46	3433.22	90.91	76.84	11.88
POP5_SA_6	3253.2	2968.49	2670.5	89.96	74.99	9.47
POP5_SA_8	4055.24	3650.37	3322.07	91.01	76.46	11.56
POP5_SA_6_2	4046.71	3665.3	3323.56	90.68	76.77	11.52

POP6_SA_1	3846.94	3449.71	3030.76	87.86	76.14	10.59
POP6_SA_4	2756.96	2507.18	2250.94	89.78	73.76	8.12
POP6_SA_6	3254.57	2923.14	2597.63	88.86	75.19	9.19
POP6_SA_9	4388.5	4058.74	3632.29	89.49	76.82	12.58
POP6_SA_11	3114.99	2753.38	2438.34	88.56	74.73	8.68
POP7_SA_9	4019.54	3616.99	3241.78	89.63	76.27	11.3
POP7_SA_11	4019.38	3646.37	3208.73	88	76.63	11.14
POP7_SA_32	3078.52	2759.99	2505.35	90.77	74.4	8.96
POP7_SA_34	3225.97	2921.48	2640.04	90.37	74.41	9.44
POP7_SA_36	2883.85	2625.33	2380.67	90.68	74.01	8.56
POP8_SA_1	3960.58	3531.78	3193.56	90.42	76.48	11.11
POP9_SA_1	3245.75	2938.01	2639.22	89.83	75.01	9.36
POP9_SA_2	3461.11	3149.99	2841.05	90.19	75.41	10.02
POP9_SA_3	2410.91	2210.59	1967.84	89.02	73.2	7.15
POP9_SA_7	4082.86	3762.02	3331.05	88.54	76.73	11.55
POP9_SA_3_2	3529.45	3224.89	2832.22	87.82	75.78	9.94
POP10_SA_2	3604.46	3318.29	3020.98	91.04	76.37	10.52
POP10_SA_4	3764.77	3385.3	3076.68	90.88	76.55	10.69
POP10_SA_5	3612.66	3257.37	2966.44	91.07	76.08	10.37
POP10_SA_10	3939.15	3598.95	3256.6	90.49	76.57	11.31
POP10_SA_11	4056.25	3670.95	3323.16	90.53	77.2	11.45
POP11_SA_1	2431.05	2198.4	1851.55	84.22	73.45	6.7
POP11_SA_3	2932.21	2547.03	2210.08	86.77	74.42	7.9
POP11_SA_4	2671.38	2402.82	2188.99	91.1	74.24	7.84
POP11_SA_7	3418.25	3121.08	2758.35	88.38	75.86	9.67
POP11_SA_8	2775.46	2354.47	2132.71	90.58	73.86	7.68
POP12_SA_1	3352.7	3012.33	2742.51	91.04	75.91	9.61
POP12_SA_2	3329.98	3051.64	2778.63	91.05	75.45	9.79
POP12_SA_3	3525.01	3128.05	2864.32	91.57	75.77	10.05
POP12_SA_7	3168.55	2880.44	2594.83	90.08	76.04	9.08
POP2_OC_1	2226.95	2004.43	1832.1	91.4	72.47	6.72
POP2_OC_4	3380.52	3021.62	2754.84	91.17	75.17	9.75
POP2_OC_5	3901.27	3354.91	3048	90.85	75.53	10.73
POP2_OC_6	3106.1	2666.64	2426.51	91	74.13	8.71
POP2_OC_8	4323.98	3584.35	3262.73	91.03	75.57	11.48
POP3_OC_1	3197.98	2924.79	2668.53	91.24	74.81	9.49
POP3_OC_4	4453.08	3984.52	3642.73	91.42	76.84	12.61
POP3_OC_7	4651.01	4173.65	3823.98	91.62	76.42	13.31
POP3_OC_8	3678.99	3280.2	2996.77	91.36	75.93	10.5
POP3_OC_10	3594.47	3181.34	2903.05	91.25	75.7	10.2
POP4_OC_9	4969.79	4118.84	3750.67	91.06	76.61	13.02
POP4_OC_22	3230.7	2938.64	2672.37	90.94	74.93	9.49
POP4_OC_23	3038.12	2713.8	1742.19	64.2	71.95	6.44

POP4_OC_24	3020.87	2737.04	2499.19	91.31	74.47	8.93
POP4_OC_25	3196.79	2842.22	1781.06	62.66	72.01	6.58
POP5_OC_1	3873.22	3399.55	3106.45	91.38	75.82	10.9
POP5_OC_2	4160.38	3702.44	3312.06	89.46	74.71	11.79
POP5_OC_5	3750.38	3345.35	2999.32	89.66	75.84	10.52
POP5_OC_9	3786.24	3366.16	3081.4	91.54	74.35	11.02
POP5_OC_12	3932.72	3454.54	3157.03	91.39	75.74	11.09
POP7_OC_5	4933	4422.93	3819.61	86.36	76.86	13.22
POP7_OC_6	4510.78	3873.65	3425.94	88.44	76.18	11.96
POP7_OC_7	4081.08	3647.39	3140.86	86.11	76.01	10.99
POP7_OC_21	2943.38	2685.43	2450.96	91.27	74.29	8.77
POP7_OC_22	3084.78	2819.02	2566.27	91.03	74.43	9.17
POP8_OC_2	2756.51	2515.13	2296.77	91.32	73.67	8.29
POP8_OC_3	2572.61	2325.44	2124.11	91.34	73.02	7.74
POP8_OC_4	3611.22	3269.46	2989.48	91.44	75.08	10.59
POP8_OC_11	2894.45	2623.85	2396.21	91.32	73.1	8.72
POP10_OC_5	3122.9	2831.92	2583.33	91.22	74.17	9.26
POP10_OC_6	2934.89	2647.27	2414.58	91.21	74.31	8.64
POP10_OC_23	3248.29	2946.93	2659.45	90.24	74.77	9.46
POP10_OC_24	2912.17	2617.2	2387.46	91.22	73.69	8.62
POP10_OC_26	2682.66	2480.42	2243.82	90.46	73.55	8.11
9_Pa10_C (*P. australiana*)	5768.55	10682.09	8632.21	80.81	73.50	31.23
PaCook_C (*P. australiana*)	6340.12	12260.21	10156.93	82.84	74.07	36.46

Coverage (%) = Covered length/Genome size (with N)×100

Mapped (%) = Mapped bases/Clean data×100

Depth = Mapped bases/Covered length

Supplementary Table 3. Numerical distribution of SNPs across different genomic regions.

	**Number**	**Percentage (%)**
Intergenic	19,423,093	48.4271
Exon	2,964,219	7.3906
Intron	12,274,425	30.6035
Start_Codon	2,055	0.0051
Stop_Codon	3,416	0.0085
Splice_Site	5,902	0.0147
Upstream	2,715,059	6.7694
Downstream	2,731,129	6.8094
Total	40,107,925	

Supplementary Table 4. Overall indel statistics of the *P. xylostella* genome.

Type	Exon	Intron	Upstream	Downstream	Intergenic	Total
Insertions	68,885	3,714,716	886,022	878,308	5,724,330	11,272,261
Deletions	79,204	3,848,621	872,028	880,085	5,784,242	11,464,180

Supplementary Table 5. SNP statistics across different geographical groups of *P. xylostella.*

Colony	Total SNP	Shared SNP	Unique SNP	Shared Percentage (%)	Unique Percentage (%)
AF	16,173,444	2,887,447	1,474,048	17.85	9.11
AS	31,671,052	2,887,447	8,504,937	9.12	26.85
EU	14,469,327	2,887,447	1,011,550	19.96	6.99
NA	17,715,042	2,887,447	2,014,467	16.30	11.37
OC	10,334,781	2,887,447	918,242	27.94	8.89
SA	9,073,953	2,887,447	648,822	31.82	7.15

SA: South America; NA: North America; A-E: Afro-Eurasia; SEA: South East Asia; OC: Oceania.

Supplementary Table 6. List of topologies with greater than 2% prevalence in the genome-wide local trees.

Topology	Proportion of windows with 5 kb SNPs
(Pa,(SA,(NA,(A-E,(SEA,OC)))))	0.117443325
(Pa,(SA,((NA,A-E),(SEA,OC))))	0.070843829
(Pa,(NA,(SA,(A-E,(SEA,OC)))))	0.056675063
(Pa,(SA,(A-E,(NA,(SEA,OC)))))	0.047858942
(Pa,(NA,((SA,A-E),(SEA,OC))))	0.034634761
(Pa,(SA,(OC,(NA,(SEA,A-E)))))	0.031171285
(Pa,((SA,(NA,A-E)),(SEA,OC)))	0.0302267
(Pa,((SA,NA),(A-E,(SEA,OC))))	0.02581864
(Pa,(SA,(NA,(OC,(SEA,A-E)))))	0.022355164
(Pa,(((SA,A-E),NA),(SEA,OC)))	0.022040302
(Pa,((NA,A-E),(SA,(SEA,OC))))	0.021410579
(Pa,(((SA,NA),(SEA,OC)),A-E))	0.020151134

Notes: Pa: *P. australiana*; SA: South America; NA: North America; A-E: Afro-Eurasia; SEA: South East Asia; OC: Oceania. A total of 3,256 local trees were built across the genome with 105 possible topologies, of which only 26 topologies were identified in greater than 1% of the genome. SA is the most common basal node from a singular bifurcation in 29% of tested windows. Alternate topologies are likely due to incomplete lineage sorting (ILS) or migration between populations.

References

1. Zalucki, M. P. & Furlong, M. J. Predicting outbreaks of a migratory pest: an analysis of DBM distribution and abundance revisited. in *Proceedings of The Sixth International Workshop on Management of Diamondback Moth and Other Crucifer Pests* (AVRDC, Taiwan, 2011).
2. Landry, J. F. & Hebert, P. D. N. *Plutella australiana* (Lepidoptera, Plutellidae), an overlooked diamondback moth revealed by DNA barcodes. *Zookeys* **327**, 43-63 (2013).
3. Chang, W. X. Z. et al. Mitochondrial DNA sequence variation among geographic strains of diamondback moth (Lepidoptera: Plutellidae). *Ann. Entomol. Soc. Am.* **90**, 590-595 (1997).
4. Robinson, G.S. & Sattler, K. *Plutella* in the Hawaiian Islands: relatives and host-races of the diamondback moth (Lepidoptera: Plutellidae). *Bishop Museum Occas. Pap.* **67**, 1-27 (2001).
5. Li, H. & Durbin, R. Inference of human population history from individual whole-genome sequences. *Nature* **475**, 493-496 (2011).
6. Zhang, C. Dong, S. Xu, J. He, W. & Yang, T. PopLDdecay: a fast and effective tool for linkage disequilibrium decay analysis based on variant call format files. *Bioinformatics*, **35,** 1786–1788 (2019).
7. Troczka, B. et al. Resistance to diamide insecticides in diamondback moth, *Plutella xylostella* (Lepidoptera: Plutellidae) is associated with a mutation in the membrane-spanning domain of the ryanodine receptor. *Insect Biochem. Molec. Biol.* **42**, 873-880 (2012).

8. Endersby, N. M. Viduka, K., Baxter, S. W. Saw, J. Heckel, D. G. & McKechnie, S. W. Widespread pyrethroid resistance in Australian diamondback moth, *Plutella xylostella* (L.), is related to multiple mutations in the para sodium channel gene. *Bull. Entomol. Res.* **101**, 393-405(2011).

9. South, A. rworldmap: a new R package for mapping global data. *R J.* **3**, 35-43 (2011).

10. Chen, G. Lee, S. H. Zhu, Z. Benyamin, B. & Robinson, M. R. EigenGWAS: finding loci under selection through genome-wide association studies of eigenvectors in structured populations. Heredity 117, 51-61 (2016).
