# Supplementary material for: Variation among 532 genomes unveils the origin and evolutionary history of a global insect herbivore
Source: Nat Commun. 2020 May 8;11:2321. doi: 10.1038/s41467-020-16178-9 (PMC7211002; doi:10.1038/s41467-020-16178-9)
Supplement: Supplementary file 3 — Reporting Summary [file 41467_2020_16178_MOESM3_ESM.pdf]

nature research

Corresponding author(s): Minsheng You

Last updated by author(s): Mar 10, 2020

Reporting Summary

Nature Research wishes to improve the reproducibility of the work that we publish. This form provides structure for consistency and transparency in reporting. For further information on Nature Research policies, see Authors & Referees and the Editorial Policy Checklist .

Statistics

For all statistical analyses, confirm that the following items are present in the figure legend, table legend, main text, or Methods section.

n/a	Confirmed	
☐	☒	The exact sample size (*n*) for each experimental group/condition, given as a discrete number and unit of measurement
☐	☒	A statement on whether measurements were taken from distinct samples or whether the same sample was measured repeatedly
☐	☒	The statistical test(s) used AND whether they are one- or two-sided<br>*Only common tests should be described solely by name; describe more complex techniques in the Methods section.*
☐	☒	A description of all covariates tested
☐	☒	A description of any assumptions or corrections, such as tests of normality and adjustment for multiple comparisons
☐	☒	A full description of the statistical parameters including central tendency (e.g. means) or other basic estimates (e.g. regression coefficient) AND variation (e.g. standard deviation) or associated estimates of uncertainty (e.g. confidence intervals)
☐	☒	For null hypothesis testing, the test statistic (e.g. *F*, *t*, *r*) with confidence intervals, effect sizes, degrees of freedom and *P* value noted<br>*Give P values as exact values whenever suitable.*
☐	☒	For Bayesian analysis, information on the choice of priors and Markov chain Monte Carlo settings
☐	☒	For hierarchical and complex designs, identification of the appropriate level for tests and full reporting of outcomes
☐	☒	Estimates of effect sizes (e.g. Cohen's *d*, Pearson's *r*), indicating how they were calculated

Our web collection on statistics for biologists contains articles on many of the points above.

Software and code

Policy information about availability of computer code

Data collection	No softwares were used for data collection.
Data analysis	Publicly avaiable softwares, including Stampy (v1.0.27), Picard-tools (v-1.117), Genome Analysis Toolkit (v-3.2.2), UnifiedGenotyper, SAMtools, SOAPsnp, PHYLIP (v3.695), MEGA5, vcftools, sNMF, pophelper, PLINK, PSMC, SMC++, EigenGWAS, Blast2GO (version 2.5.0), WEGO, Prime module of Schrödinger software and Chimera are detailed in the section of Methods.

For manuscripts utilizing custom algorithms or software that are central to the research but not yet described in published literature, software must be made available to editors/reviewers. We strongly encourage code deposition in a community repository (e.g. GitHub). See the Nature Research guidelines for submitting code & software for further information.

Data

Policy information about availability of data

All manuscripts must include a data availability statement. This statement should provide the following information, where applicable:

- Accession codes, unique identifiers, or web links for publicly available datasets
- A list of figures that have associated raw data
- A description of any restrictions on data availability

The data that support the findings of this study have been deposited in the CNSA (https://db.cngb.org/cnsa/) of CNGBdb with accession code CNP0000018.

Field-specific reporting

Please select the one below that is the best fit for your research. If you are not sure, read the appropriate sections before making your selection.

☐ Life sciences ☐ Behavioural & social sciences ☒ Ecological, evolutionary & environmental sciences

Ecological, evolutionary & environmental sciences study design

All studies must disclose on these points even when the disclosure is negative.

Study description	Based on the globally-distributed nature of diamondback moth, plutella xylostella, we performed a phylogographical and population genetics study on our global samples.
Research sample	Diamondback moths, Plutella xylostella, (regardless of age and sex) were collected from cruciferous vegetable fields in each sampling locations. Field-collected samples were morphologically inspected and genetically checked with COI sequences to confirm their identity. The samples were preserved in 95% alcohol at -80℃ prior to DNA extraction.
Sampling strategy	We used an average of five (fully sequenced) individuals per site to give a robust 'picture' of the genomic variability among individuals for that site as well as to compare and contrast with individual from other sites. The number of sites was set to give comprehensive coverage of all geographical regions in which this species is present, including all zones that earlier work had suggested to be the origin.
Data collection	Within each sampling location, larvae, pupae, and adults were collected from cruciferous vegetable fields by our team members and local entomologists.
Timing and spatial scale	The global sample of P. xylostella was collected during 2012-2014 from 114 locations that cover broad regions throughout the world, with 13 samples from Africa and Madagascar, 43 samples from Asia, 13 samples from Europe, 26 samples from North America including Hawaii, 12 samples from South America, and 7 samples from Oceania. Our collection covered an extensive scope of the eco-climatic index and areas that support differing numbers of annual generations, including those regions with year-round persistence of P. xylostella to others that are only seasonably suitable for growth and development of the species
Data exclusions	Individuals with poor DNA quality were excluded. Also excluded were individuals with resequencing data that yielded low mapping rate (<60%) and low genome coverage (<60%). None of the individuals that yielded adequate quality data were excluded for any of the regions or sites.
Reproducibility	An average of approximately five individuals from each of the sampling locations were used for DNA extraction and sequencing, making a total of 532 individuals (with adequate quality data) in 114 locations across 55 countries worldwide. Using nuclear and mitochondrial genomes as well as COI sequences of 532 individual samples, we analyzed the phylogenetic relationships and uncovered the origin and expansion routes of P. xylostella. We believe that our findings are convincing and reproducible.
Randomization	Our sampling locations (114) were randomly selected in different regions according to the geographical and climatic conditions that are suitable for growth and development of P. xylostella. Within each of the locations, larvae, pupae and adults were randomly collected from cruciferous vegetable fields.
Blinding	To avoid unintentional biases, the samples were each allocated a code number that was cryptic in not allowing anyone involved in handling or analysis to identify the origin of the insect, DNA or associated genomic data. Only at the late stage of tree construction were the samples re-identified.

Did the study involve field work? ☐ Yes ☒ No

Reporting for specific materials, systems and methods

We require information from authors about some types of materials, experimental systems and methods used in many studies. Here, indicate whether each material, system or method listed is relevant to your study. If you are not sure if a list item applies to your research, read the appropriate section before selecting a response.

Materials & experimental systems

n/a	Involved in the study
☒	☐ Antibodies
☒	☐ Eukaryotic cell lines
☒	☐ Palaeontology
☐	☒ Animals and other organisms
☒	☐ Human research participants
☒	☐ Clinical data

Methods

n/a	Involved in the study
☒	☐ ChIP-seq
☒	☐ Flow cytometry
☒	☐ MRI-based neuroimaging

Animals and other organisms

Policy information about studies involving animals; ARRIVE guidelines recommended for reporting animal research

Laboratory animals	The study did not involve laboratory animals

Wild animals	The study did not involve wild animals.
Field-collected samples	Our field-collected samples were preserved in 95% alcohol at -80℃ prior to DNA extraction.
Ethics oversight	No ethical approval was required since our samples are insect pests.

Note that full information on the approval of the study protocol must also be provided in the manuscript.
