# Supplementary material for: Variation among 532 genomes unveils the origin and evolutionary history of a global insect herbivore
Source: Nat Commun. 2020 May 8;11:2321. doi: 10.1038/s41467-020-16178-9 (PMC7211002; doi:10.1038/s41467-020-16178-9)
Supplement: Supplementary file 2 — Peer Review [file 41467_2020_16178_MOESM2_ESM.pdf]

Reviewers' comments:

Reviewer #1 (Remarks to the Author):

This manuscript describes a population genomics study of 532 resequenced genomes of P. xylostella. The section on the geographic origin and expansion of this species is sound and provides interesting new information. However beyond this the study provides limited new information. In the absence of any phenotypic data the authors have attempted to perform a GWAS using a group of the samples from regions where insecticide use may be highest as an artificial phenotype. I find this section much less convincing.

A huge amount of work had been done on P. xylostella to discover and validate genes and mutations that confer resistance to a wide range of insecticides. Thus I am puzzled as to the why the authors did not simply look for known mutations in insecticide target-sites or genes associated with metabolic resistance. It would have been very interesting to establish the frequency of these important resistance alleles globally.

The authors state: ‘However, in the absence of molecular structure of the target proteins, the effect of mutations on interactions of the related enzymes or receptors with insecticides cannot be fully understood42.’ If I have interpreted this sentence correctly then I disagree with this conclusion – the effect of mutations in insecticide target-sites can be assessed in several ways and many of these do not require structural information – for example functional characterisation of the effects of mutations on insecticide binding.

A particularly weak section of the manuscript relates to the prediction of the effects of mutations based on modelling. In contrast to the authors claim this approach does not prove the causal role of mutations in resistance and can only be used as a tool once this has been established using functional approaches.

The following paragraph is clumsy and requires revision:
The outliers contained 1,179 candidate genes, being more overrepresented in metabolic and molecular signaling-related pathways than other pathways (Fig. 4b and Supplementary Figs. 11 and 12), and mostly associated with insecticide resistance through regulation of gene expression and/or mutation of target sites34, 40, 41. This further illuminates the genetic and molecular bases of how P. xylostella can rapidly evolve the capacity of insecticide resistance11.

Reviewer #2 (Remarks to the Author):

This is an impressive study, using an extremely large data-set and a performing some interesting analyses of these data. There are some very interesting findings, in particular that this widespread insect pest species originated in South America, something that was not previously suspected. This has important implications for our understanding of the ecology of this species and also raises the question of how many other organisms may have followed this human-mediated migration route within the last 500 years. It is mentioned that there are other endemic Plutella species in South America and I wondered if any of these were included in the COI tree and if so, if there is any evidence that they may be closer to P. xylostella than P. australiana, which is given as the closest known relative. I felt this should at least be mentioned even if the evidence is missing or inconclusive either way. The COI tree in the supplementary information is too small (low resolution) to read the labels.

A large number of loci putatively under selection in the most distant populations (Oceania and SE Asia) are also identified. I am not convinced by the statements that these "are involved in

evolutionary adaptation under the strong selective pressure of insecticides." (line 236 and elsewhere). These populations presumably encountered many novel challenges in the environments they colonised, so it seems unlikely that all selected changes are driven by insecticides. No functional evidence is presented and while the structural modelling data is perhaps consistent with a role in resistance evolution, this isn’t very clear from the data that is presented without having to go into some depth in the supplementary information. In addition, it is for just 3 genes out of over one thousand identified in the outlier scan. It is not very clear how or why these three were selected and this comes across as cherry picking. If this was presented simply as " we have identified some putative novel insecticide resistance mutations amongst the selected loci" rather than all selection being due to resistance, I think it would be OK.

I also think the GO and KEGG pathway “enrichment” analysis is flawed. From my reading of the text and methods I do not think that any enrichment analysis was performed – ie. I don’t think any tests were performed to determine whether the observed pathways and GO terms were present in the selected gene data set in a higher proportion than would be expected by chance. If this analysis was done, then it should be clarified in the text and the appropriate statistics reporting the level of enrichment and significance should be given. I am not a big fan of enrichment analyses anyway as it seems too easy to make the results fit around any given story.

I would have preferred that further tests for selection had been performed. For example, based on runs of homozygosity and/or the site frequency spectrum. These types of tests run on the different populations might have allowed some interesting things to be said about the effect and strength of selection acting at different points and in different places during the colonisation history. The patterns of genetic diversity don’t seem to follow what I would have expected – namely highest diversity in the ancestral population and reducing diversity further away from this as the dispersing populations go through founder events and genetic bottlenecks. Rather, the opposite seems to be the case. This seems to suggest high mutation rates and strong selection, but some explicit tests of this would be very interesting. This could lead to some interesting and novel insights into the important topic of invasion biology more broadly and particularly in insects.

I have some queries about some analyses and the way these are presented. The text and figure 2 legend hints that the best supported K value in the sNMF analysis is 5 but the statistics to back this up are not given, nor is the analysis method used find the optimum K value described in the methods. It is not very clear to me why, in figure 3 so many distinct “populations” are coloured in Eurasia and Africa. What analysis are these populations based on? To my mind, the best way to assign these populations would have been from a PCA, or similar analysis, followed by clustering. Related to this, I think the PCA used for the EigenGWAS should be included in the main figures, since it underpins the selection results. Further, it is not stated in the methods or figure 4 legend which populations were used for the Fst calculations. Also, no information is given on what Fst is at other loci in the genome, so we have no comparison point for the values shown in figure 4.

The methods and Figure 3 legend both say that “multiple” individuals were used to infer the demographic history but do not say how many or how these were selected. If not all the individuals were used it would be interesting to know if similar results would be obtained with different subsets of individuals from each population. I am a bit confused as to why the Oceania and South East Asian populations show less of a dip in Ne ~500 ya than the Afro-Eurasian population. This does not seem to be consistent with the proposed scenario of the former two splitting from the latter after this.

I think the final 2 sentences could be strengthened to say something more about the biological implications rather than focussing on the methods, which to me do not seem to be particularly novel (besides the sheer quantity of data). As far as I can tell none of the methods used in this paper are completely new and all have been used is previous publications (as cited in the text).

Overall, I think this is an impressive and important study, worthy of publication in a broad readership

journal like Nature Communications. There are some additional analyses that I would like to see performed, although I don't believe there are any major problems with the analysis as it stands (other than the enrichment analysis). I think the statements about selection for insecticide resistance should be toned down and some additional information is needed in the methods and figure legends.

Minor comments:

Title: I wonder if the word "insect" should be included, since non-specialist readers are more likely to think of large vertebrate herbivores.

Line 72: I think "are" should be "were"

Line 224: "and allows us to understand how the structure features affect the activities of enzymes" is not grammatically correct.

I found figure 1B to be fairly uninformative, besides showing that the genome is highly fragmented (which I don't see as a major problem). There are no axes shown for the SNPs, nuc. div. or indels, so the absolute values can't be inferred and it is not clear if there is a pattern and if so what we should infer from this. If there is a pattern this is not mentioned in the main text. I would remove this panel to the supplementary information.
Figure 1C: It would be helpful for the reader if the colours in this were consistent with those in figure 1A. Also the legend is not needed as this give the same information as the x-axis.

Figure 2 legend: I think should include the name of the program used for the structure analysis

Figure 2A: Do the branch lengths represent genetic distance here? If so a scale bar should be included. It is also remarkable how short the branch to the outgroup is relative to the branch lengths with P. xylostella.

Figure 3A: The moth image in South America is not very clear (I thought it was a mountain range at first) and a bit distracting.

Figure 4A: Again, I don't find this very informative and it doesn't really link with any point in the text. Was any analysis on this done to see if any of these categories were enriched?

Figure 4B: The legend does not state what the bars show. Is this enrichment or just numbers of genes? A "background" Fst level is needed for comparison.

Supplementary figure 12 is not referred to in the text and is not fully explained in terms of what the connections between the nodes mean or how these were calculated.

With best wishes,
Nicola Nadeau

Reviewer #3 (Remarks to the Author):

Minsheng You and colleagues collected an impressive dataset of diamondback moth genomic data, not only in number (n=532), but also in covering its worldwide distribution. They used these data to reconstruct the phylogenetic relationships of these populations and the recent evolutionary history of diamondback moth. Using knowledge of the population structure, they also perform a divergence test, and identify increased divergence in 1,179 genes that is potentially related to increased insecticide use. Three of these genes included a non-synonymous substitution that the authors argue may relate to insecticide resistance.

The paper is well written, and the sampling certainly merits publication in a high impact journal like Nature Communications. However, I find the analysis preliminary and I am not completely convinced about the inferences on the recent evolutionary history of the moths and the insights obtained of selection and evolution of insecticide resistance.

First, the result of the historical demography are confusing and these type of analysis have notoriously bad resolution at very recent time scales. Regarding the PSMC analysis, different geographic areas seem to have vastly different historical population size estimates. If these populations all originate from SA less than 500 years ago, shouldn't they be more similar? What may cause this? Similarly, when considering just the relative changes in population sizes, the divergence of population sizes among different geographic areas occurs around 4000-5000 years ago, which is far older than the claimed expansion 500 years ago. Why not run this analysis on e.g. ten individuals (or all) of each geographic region, to see how consistent the estimates are?
Regarding the SMC++ analysis, the finding of a population size dip at ~500 years ago is impressive. However, the original paper that describes SMC++ does not consider any time frames more recent than 1000 years ago. Does the method truly provide the power to infer such recent events? Like for the PSMC analysis, I would expect that before 500 years ago, all these populations had the same sizes. What could cause the different estimates among geographic regions?

Second, it is not clearly explained how the authors come up with the sequence of dispersal events that have given rise to the current distribution of diamondback moth (Figure 3A). Moreover, I find that the patterns of SNP diversity are not consistent with a recent origin from South America. Wouldn't one expect to find the highest diversity in the ancestral population, given that the expansion is so recent? The interpretation of this expansion history is further complicated by the authors subdivision of populations only in Asia. It is not clear to me how and why this is done only for Asia.

Third, the SNP coverage and statistics are not very clearly presented in regard to the geographic sampling. I think adding more transparency in these aspects could give more confidence in the results and take away any potential worry that patterns in e.g. the PCA analysis could result from local differences in genotype quality.

Forth, the selection scan is not explained in the text and the reasoning behind picking a PCA axis along which to perform the GWAS analysis completely eludes me. Is it supposed to represent a gradient of selection? If so, could it not be confounded by geography when comparing AS and OC?

Overall, I think the dataset has more potential to it and should allow more extensive analysis that could provide more convincing insights into insecticide resistance in this species. Selective sweep analysis for instance could provide additional evidence for selected targets. Despite the vast sampling, the authors restricted themselves to using an eigenvector approach for GWAS. Are there any recorded environmental factors that repeat in separated geographic regions that could be correlated? This could potentially highlight repeated selection of adaptive alleles in response to insecticides.

Additional comments:

L 62-64: "Insights into the evolutionary history of P. xylostella provide better understanding of how it has become a highly successful herbivore worldwide." If space permits, I would be interested to read at this point what these insights are and how they relate to their worldwide spread.

L 144-145: "A-E clade containing multiple sub-clades"

Suppl Figure 8: LD decay over 16-35 bp is extremely fast! Are you certain the scale is not in kbp?

L195-212: I do not understand the eigenvector approach. What is this supposed to control for or what

is this axis supposed to represent? Geography or differentiation? If this axis represents adaptation to insecticides, is it not confounded by geography as well (South East Asia and Oceania being close to each other)?

L206: 1,179 genes is a very large number. These could include a large number of false positives. Can your GWAS approach give any idea of what percentage may be real?

L209: Are these genes directly associated with insecticide resistance?

L221-225: How do the predicted structures inform you about enzymatic activities? Did any other genes show point mutations?

L500: 399 sequence, how do these add up to the 532 P. xylostella used (e.g. L 550)?

L520-522: Were the artificial chromosomes constructed according to an existing linkage map?

L528-532: How does the baserecalibration work if the reference SNP set was extracted from the current dataset?

L539-541: What average read coverage per genotype do these QC filter values result in?

L 559: Phylogenetic tree: How many SNPs were eventually included in this tree? Have you considered using more advanced tree building methods for whole genome data using FastTree?

L599-600: What were the criteria used to define these clusters?

L613-614: What arguments are there that the Drosophila mutation rate should match that of P. xylostella?

Figures:

The color schemes in Figure 1A, 1C and 2A should match, which is not the case.

Figure 1A. Perhaps points can be sized according to the number of samples from each population.

Figure 1B. This plot should have a legend and y-axis labels. How are SNPs and indels scaled here? Also, the unassigned proportion is large. How are these scaffolds ordered and how should one interpret patterns of changes in nucleotide diversity here? Perhaps the spatial information of the unordered scaffolds is confusing and unnecessary and it might be better to remove this information and only show average for the unordered scaffolds.

Figure 1C. The legend here is redundant. I would just add an x-axis title.

Figure 2A. Why not present the rooted tree, given that you included an outgroup?

Figure 3A. What is the scale for the pie chart sizes related to the sampling size? The moth on top if the figure is hard to see. How are the clusters A-E identified? Why are no clusters like that identified in the ancestral populations? In case of a recent spread, I would be surprised that a recently colonized area is so diverse.

Responses to the Reviewers' Comments

Reviewers' comments:

Reviewer #1 (Remarks to the Author):

This manuscript describes a population genomics study of 532 resequenced genomes of P. xylostella. The section on the geographic origin and expansion of this species is sound and provides interesting new information. However beyond this the study provides limited new information. In the absence of any phenotypic data the authors have attempted to perform a GWAS using a group of the samples from regions where insecticide use may be highest as an artificial phenotype. I find this section much less convincing.

A huge amount of work had been done on P. xylostella to discover and validate genes and mutations that confer resistance to a wide range of insecticides. Thus I am puzzled as to the why the authors did not simply look for known mutations in insecticide target-sites or genes associated with metabolic resistance. It would have been very interesting to establish the frequency of these important resistance alleles globally.

We thank you for the suggestion. We have established the frequency of reported resistance alleles globally and presented a new figure (Supplementary Fig. 9) and new statements in the main text to illuminate the global genotype distribution of three insecticide-resistance-related point mutation loci, which have strong geographical dependence. Such a finding further supports our argument that *P. xylostella* is well suited for a study of evolutionary adaptation under the strong environmental selection pressure, by showing differentiated genotype (allele) frequency resulting from differentiated selective pressure in various geographic areas.

The authors state: 'However, in the absence of molecular structure of the target proteins, the effect of mutations on interactions of the related enzymes or receptors with insecticides cannot be fully understood42.' If I have interpreted this sentence correctly then I disagree with this conclusion – the effect of mutations in insecticide target-sites can be assessed in several ways and many of these do not require structural information – for example functional characterisation of the effects of mutations on insecticide binding.

The sentence has been rewritten as: "Identification of molecular structure of the target proteins may help understand the effect of mutations on interactions of the related enzymes or receptors with insecticides".

A particularly weak section of the manuscript relates to the prediction of the effects of mutations based on modelling. In contrast to the authors claim this approach does not prove the causal role of mutations in resistance and can only be used as a tool once this has been established using

functional approaches.

Thank you for your kind suggestion. The statement has been changed to the modelling result "provides a cue for further experimentally-based research to establish the functional relationship between mutations and insecticide resistance" in the text.

The following paragraph is clumsy and requires revision:
The outliers contained 1,179 candidate genes, being more overrepresented in metabolic and molecular signaling-related pathways than other pathways (Fig. 4b and Supplementary Figs. 11 and 12), and mostly associated with insecticide resistance through regulation of gene expression and/or mutation of target sites34, 40, 41. This further illuminates the genetic and molecular bases of how P. xylostella can rapidly evolve the capacity of insecticide resistance11.

The sentence has been rewritten as: "These outliers contained 1,179 candidate genes, being more overrepresented in metabolic and molecular signaling-related pathways than any other pathways (Fig. 4b and Supplementary Figs. 11 and 12). The majority of the 1179 candidate genesare associated with environmental adaptation, detoxification of plant defense compounds, insecticide resistance through regulation of gene expression or mutation of target sites[34, 40, 41]. These findings further clarify the genetic and molecular bases of how *P. xylostella* can rapidly evolve the capacity of xenobiotic detoxification[11]."

Reviewer #2 (Remarks to the Author):

This is an impressive study, using an extremely large data-set and a performing some interesting analyses of these data. There are some very interesting findings, in particular that this widespread insect pest species originated in South America, something that was not previously suspected. This has important implications for our understanding of the ecology of this species and also raises the question of how many other organisms may have followed this human-mediated migration route within the last 500 years. It is mentioned that there are other endemic Plutella species in South America and I wondered if any of these were included in the COI tree and if so, if there is any evidence that they may be closer to P. xylostella than P. australiana, which is given as the closest known relative. I felt this should at least be mentioned even if the evidence is missing or inconclusive either way. The COI tree in the supplementary information is too small (low resolution) to read the labels.

First, we did present a COI tree with high resolution (an independent pdf file) that allows all labels to be readable. Such a COI tree provide robust and convincing evidence that *P. xylostella* individuals from South America are evolutionarily closer to outgroup species. The related statement in the main text has been revised to "Using the nuclear SNP data, a neighbor-joining (NJ) tree was constructed for global *P. xylostella* populations with the congeneric *P. australiana* as an outgroup according to our *COI*-based phylogenetic analysis (see Methods)".

We did not collect any described *Plutella* species (except *Plutella xylostella*) from South America, but one of our co-authors, Dr. Simon Baxter, had sequenced complete mitochondrial genomes for

two *Plutella* species found in North America, *Plutella armoraciae* and *Plutella porrectella*, as well as a more distantly related onion moth, *Acrolepiopsis assectella*. Using the mitochondrial genome sequences, Simon constructed a phylogenetic tree for these species (as shown in the following figure), which provides additional evidence that *P. australiana* is the best sequenced outgroup.

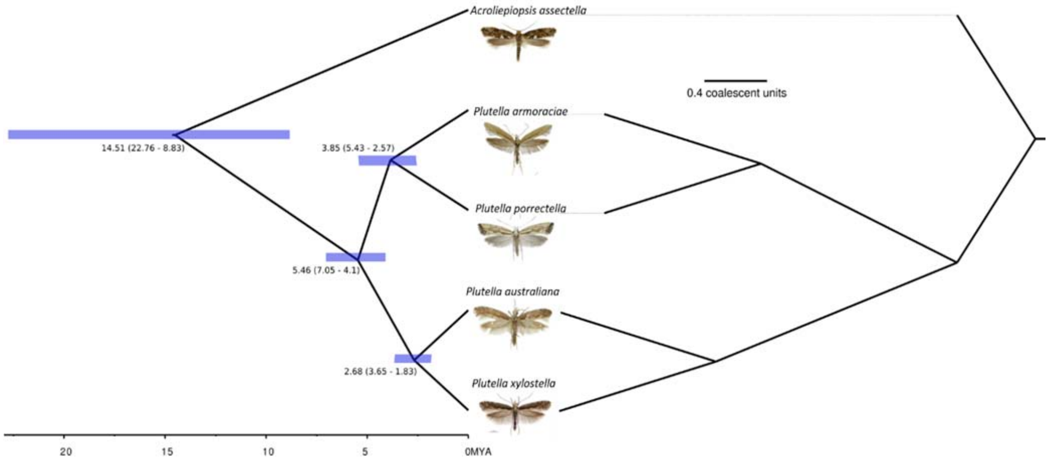

Figure legend: Mitochondrial (left) chronogram and nuclear species tree (right) for four *Plutella* species and the outgroup *Acrolepiopsis assectella*. LEFT: COX1 and 16S were used to estimate the mitochondrial divergence date of four *Plutella* species. RIGHT: 104 nuclear single copy orthologs were used to construct a maximum likelihood phylogeny using Astral III. All nodes had 1 posterior probability support.

A large number of loci putatively under selection in the most distant populations (Oceania and SE Asia) are also identified. I am not convinced by the statements that these "are involved in evolutionary adaptation under the strong selective pressure of insecticides." (line 236 and elsewhere). These populations presumably encountered many novel challenges in the environments they colonised, so it seems unlikely that all selected changes are driven by insecticides. No functional evidence is presented and while the structural modelling data is perhaps consistent with a role in resistance evolution, this isn't very clear from the data that is presented without having to go into some depth in the supplementary information. In addition, it is for just 3 genes out of over one thousand identified in the outlier scan. It is not very clear how or why these three were selected and this comes across as cherry picking. If this was presented simply as " we have identified some putative novel insecticide resistance mutations amongst the selected loci" rather than all selection being due to resistance, I think it would be OK.

The statements have been revised in the text accordingly to:
"Genes over-represented in metabolic and molecular signaling-related pathways are putative candidates involved in evolutionary adaptation under the strong selective pressure, resulting from challenges of xogenous factors, such as insecticides".

I also think the GO and KEGG pathway "enrichment" analysis is flawed. From my reading of the

text and methods I do not think that any enrichment analysis was performed – ie. I don't think any tests were performed to determine whether the observed pathways and GO terms were present in the selected gene data set in a higher proportion than would be expected by chance. If this analysis was done, then it should be clarified in the text and the appropriate statistics reporting the level of enrichment and significance should be given. I am not a big fan of enrichment analyses anyway as it seems too easy to make the results fit around any given story.

We did not do any statistical analysis, but only identify enrichment of GO and KEGG pathways with high gene numbers (top 20 categories) under selection (with derived alleles). Such a result provides additional evidence supporting that diamondback moth may become a successful herbivore with worldwide distribution by the enrichment of metabolic and signaling pathways.

I would have preferred that further tests for selection had been performed. For example, based on runs of homozygosity and/or the site frequency spectrum. These types of tests run on the different populations might have allowed some interesting things to be said about the effect and strength of selection acting at different points and in different places during the colonisation history. The patterns of genetic diversity don't seem to follow what I would have expected – namely highest diversity in the ancestral population and reducing diversity further away from this as the dispersing populations go through founder events and genetic bottlenecks. Rather, the opposite seems to be the case. This seems to suggest high mutation rates and strong selection, but some explicit tests of this would be very interesting. This could lead to some interesting and novel insights into the important topic of invasion biology more broadly and particularly in insects.

As suggested, we have done additional analyses of homozygosity and site frequency spectrum based on two "separated" populations identified by EigenWAS, which did find a set of overlapping genes between two methods. However, due to high heterozygosity/recombination across the genome, the method based on individual loci suits better than the homozygosity-related method.

P. xylostella is an unusual insect species with remarkable genetic plasticity (Henniges-Janssen et al., 2011) and high level of genomic variation (You et al., 2013) that enable it to rapidly adapt to local environments, potentially leading to the change in its genetic diversity. This may explain why a higher genetic diversity was identified from the derived populations in the regions with intensive human management (eg., overuse of broad-spectrum insecticides; Furlong et al., 2013) than the ancestral populations in South America. Comparable findings were reported for *Apis mellifera* that recent human management has increased the genetic diversity of domestic populations in comparison of ancestral wild populations (Wallberg, 2014; Nature Genetics, 46: 1081-1088).

I have some queries about some analyses and the way these are presented. The text and figure 2 legend hints that the best supported K value in the sNMF analysis is 5 but the statistics to back this up are not given, nor is the analysis method used find the optimum K value described in the

methods. It is not very clear to me why, in figure 3 so many distinct "populations" are coloured in Eurasia and Africa. What analysis are these populations based on? To my mind, the best way to assign these populations would have been from a PCA, or similar analysis, followed by clustering. Related to this, I think the PCA used for the EigenGWAS should be included in the main figures, since it underpins the selection results. Further, it is not stated in the methods or figure 4 legend which populations were used for the Fst calculations. Also, no information is given on what Fst is at other loci in the genome, so we have no comparison point for the values shown in figure 4.

The best K value (k=5) was supported by the following figure, with values of the cross-entropy criterion for sNMF runs.

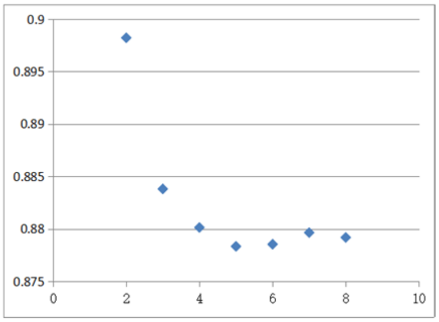

The coloured populations were assigned in Eurasia and Africa (Figure 3a) based on lineages identified in the global phylogeny. We have added a new figure to show the result of PCA analysis (Fig. 2c).

We used EigenGWAS to identify 75 individuals from Southeast Asia and Oceania (Supplementary Figure 9). Fst values of the outliers are at the top 1% of the all loci (median value of the Fst is 0.0169).

The methods and Figure 3 legend both say that "multiple" individuals were used to infer the demographic history but do not say how many or how these were selected. If not all the individuals were used it would be interesting to know if similar results would be obtained with different subsets of individuals from each population. I am a bit confused as to why the Oceania and South East Asian populations show less of a dip in Ne ~500 ya than the Afro-Eurasian population. This does not seem to be consistent with the proposed scenario of the former two splitting from the latter after this.

We randomly selected 15 individuals from each group (but also considered the sub-lineage information, e.g. avoid sampling two or more individuals from the same sub-lineages), and generated a composite likelihood based on different distinguished individuals, which may help improve the accuracy of estimation. For each group, they did show some variation in terms of population size, but recent bottlenecks at around 500 years of all groups were consistently identified. Individuals from SEA are those sampled from Indonesia and Malaysia as these two

countries are supposed to be the origin of diamondback moth in Oceania.

I think the final 2 sentences could be strengthened to say something more about the biological implications rather than focussing on the methods, which to me do not seem to be particularly novel (besides the sheer quantity of data). As far as I can tell none of the methods used in this paper are completely new and all have been used is previous publications (as cited in the text).

The statements have been revised in the text accordingly to:
"Our results illustrate the utility of emerging genomic approaches to understand historical patterns of biological expansion, and further address the underlying mechanisms associated with the worldwide dispersal of a notorious pest species".

Overall, I think this is an impressive and important study, worthy of publication in a broad readership journal like Nature Communications. There are some additional analyses that I would like to see performed, although I don't believe there are any major problems with the analysis as it stands (other than the enrichment analysis). I think the statements about selection for insecticide resistance should be toned down and some additional information is needed in the methods and figure legends.

Minor comments:

Title: I wonder if the word "insect" should be included, since non-specialist readers are more likely to think of large vertebrate herbivores.

Revised accordingly.

Line 72: I think "are" should be "were"

Revised accordingly.

Line 224: "and allows us to understand how the structure features affect the activities of enzymes" is not grammatically correct.

Revised accordingly.

I found figure 1B to be fairly uninformative, besides showing that the genome is highly fragmented (which I don't see as a major problem). There are no axes shown for the SNPs, nuc. div. or indels, so the absolute values can't be inferred and it is not clear if there is a pattern and if so what we should infer from this. If there is a pattern this is not mentioned in the main text. I would remove this panel to the supplementary information.

A scale bar is now provided as suggested.

Figure 1C: It would be helpful for the reader if the colours in this were consistent with those in figure 1A. Also the legend is not needed as this give the same information as the x-axis.

Revised as suggested.

Figure 2 legend: I think should include the name of the program used for the structure analysis.

Revised as suggested.

Figure 2A: Do the branch lengths represent genetic distance here? If so a scale bar should be included. It is also remarkable how short the branch to the outgroup is relative to the branch lengths with P. xylostella.

For a phylogeography-related study, it is not uncommon to show a phylogeny without including a scale bar (e.g., the comparable study on monarch butterfly ;Nature ,2014 ,514, 317–321), as long as the phylogeny is convincing enough in terms of showing both the interspecific and intraspecific evolutionary relationship.

Also, you can see from the following tree that, by incorporating the information of branch length, the outgroup species (*P. Australiana*) is evolutionarily distant from *P. xylostella*. In Figure2a, we only present a phylogeny that clearly shows the basal clade and evolutionary relationships among our sampling populations of *P. xylostella* (Fig. 2a, and Supplementary Fig. 3).

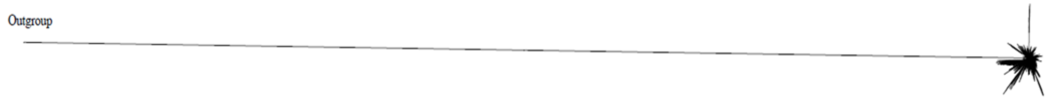

Figure 3A: The moth image in South America is not very clear (I thought it was a mountain range at first) and a bit distracting.

Revised as suggested.

Figure 4A: Again, I don't find this very informative and it doesn't really link with any point in the text. Was any analysis on this done to see if any of these categories were enriched?

Thanks for your kind comments. The related statement has been revised accordingly in the text to "We identified 3,827 SNPs (top 1%) (Fig. 4a, right) with high level of genetic differentiation (FST) from 64,960 filtered SNPs (Fig. 4a, left), which indicates that numerical distribution of the significantly differentiated SNPs is related to the density of SNPs in each of the four genomic regions (Fig. 4a)".

Figure 4B: The legend does not state what the bars show. Is this enrichment or just numbers of

genes? A “background” Fst level is needed for comparison.

It is the numbers of genes. A “background” Fst value has been provided in the legend.

Supplementary figure 12 is not referred to in the text and is not fully explained in terms of what the connections between the nodes mean or how these were calculated.

Supplementary Figure 12 was provided, together with Figure 4b and Supplementary Figure 13, to show how genes under strong selection were significantly overrepresented in metabolic and molecular signaling-related pathways. These figures are now referred on page 11 of the text.

With best wishes,
Nicola Nadeau

Reviewer #3 (Remarks to the Author):

Minsheng You and colleagues collected an impressive dataset of diamondback moth genomic data, not only in number (n=532), but also in covering its worldwide distribution. They used these data to reconstruct the phylogenetic relationships of these populations and the recent evolutionary history of diamondback moth. Using knowledge of the population structure, they also perform a divergence test, and identify increased divergence in 1,179 genes that is potentially related to increased insecticide use. Three of these genes included a non-synonymous substitution that the authors argue may relate to insecticide resistance.

The paper is well written, and the sampling certainly merits publication in a high impact journal like Nature Communications. However, I find the analysis preliminary and I am not completely convinced about the inferences on the recent evolutionary history of the moths and the insights obtained of selection and evolution of insecticide resistance.

First, the result of the historical demography are confusing and these type of analysis have notoriously bad resolution at very recent time scales. Regarding the PSMC analysis, different geographic areas seem to have vastly different historical population size estimates. If these populations all originate from SA less than 500 years ago, shouldn’t they be more similar? What may cause this? Similarly, when considering just the relative changes in population sizes, the divergence of population sizes among different geographic areas occurs around 4000-5000 years ago, which is far older than the claimed expansion 500 years ago. Why not run this analysis on e.g. ten individuals (or all) of each geographic region, to see how consistent the estimates are?
Regarding the SMC++ analysis, the finding of a population size dip at ~500 years ago is impressive. However, the original paper that describes SMC++ does not consider any time frames more recent than 1000 years ago. Does the method truly provide the power to infer such recent events? Like for the PSMC analysis, I would expect that before 500 years ago, all these populations had the same sizes. What could cause the different estimates among geographic regions?

PSMC does not have enough resolution to support estimation of recent demographic history, but we did find a similar trend in the change of population size more than 4000 years ago. The divergence of population sizes for different groups between 500-4000 may be partly due to variation of individuals, or limitation of algorithm applied. We then used the SMC++ to estimate the recent demographic history. *P. xylostella* is an insect pest with very short generation time. For example, finch and human will have 500 and 40 generations in 1000 years respectively, while for *P. xylostella* the number would be ~12,048. Thus, the SMC++ is accurate enough to estimate effective population size of an insect pest with very short generation time. A recently published paper suggests that SMC++ is the most reliable current method to infer history between ~300 generations before present (gbp) and 10,000 gbp (Patton et al. 2019; Molecular Biology and Evolution).

Second, it is not clearly explained how the authors come up with the sequence of dispersal events that have given rise to the current distribution of diamondback moth (Figure 3A). Moreover, I find that the patterns of SNP diversity are not consistent with a recent origin from South America. Wouldn't one expect to find the highest diversity in the ancestral population, given that the expansion is so recent? The interpretation of this expansion history is further complicated by the authors subdivision of populations only in Asia. It is not clear to me how and why this is done only for Asia.

For insect species, it is not uncommon that populations from the evolutionary origin do not show the highest genetic diversity. Short life span, high fecundity, extremely panmictic mating system and intensive migration are major factors that contribute to the high genetic diversity with high recombination (Supplementary Figure 8). Comparable findings were reported for *Apis mellifera* (Wallberg, 2014; Nature Genetics, 46: 1081-1088).

Subdivision of populations is the result generated by the sNMF, and it is likely resulted from intensive migration.

Third, the SNP coverage and statistics are not very clearly presented in regard to the geographic sampling. I think adding more transparency in these aspects could give more confidence in the results and take away any potential worry that patterns in e.g. the PCA analysis could result from local differences in genotype quality.

We filtered the SNPs with a very strict threshold, which was detailed in the Method section. Basic information on SNPs and sequencing were provided in the Supplementary table 2.
A new figure of PCA is provided (Fig. 2c), and the SNPs coverage presented in the Method section.

Forth, the selection scan is not explained in the text and the reasoning behind picking a PCA axis along which to perform the GWAS analysis completely eludes me. Is it supposed to represent a gradient of selection? If so, could it not be confounded by geography when comparing AS and OC?

EigenGWAS is a method used to identify loci contributing to adaptation and can also be used to account for population stratification. All sampling individuals were allocated into two groups separated by a threshold of eigenvector1 value (the value = 0). One of the two groups contains 75 individuals from Southeast Asia and Oceania, while the remaining individuals were included in the other group.

Overall, I think the dataset has more potential to it and should allow more extensive analysis that could provide more convincing insights into insecticide resistance in this species. Selective sweep analysis for instance could provide additional evidence for selected targets. Despite the vast sampling, the authors restricted themselves to using an eigenvector approach for GWAS. Are there any recorded environmental factors that repeat in separated geographic regions that could be correlated? This could potentially highlight repeated selection of adaptive alleles in response to insecticides.

Given the rapid decay of linkage disequilibrium for *P. xylostella*, we doubt that selective sweep would be a good approach to identify genomic signatures of local adaptation/selection. However, the global distribution of the nonsynonymous genotypes of three genes with point mutations (candidates selected for further Homology modelling analysis, Supplementary Fig. 14), presented in Supplementary Fig. 11, does validate the robustness and power of the GWAS analysis by showing that majority of the mutants were identified in SEA and OC.

Additional comments:

L 62-64: “Insights into the evolutionary history of P. xylostella provide better understanding of how it has become a highly successful herbivore worldwide.” If space permits, I would be interested to read at this point what these insights are and how they relate to their worldwide spread.

Revised as suggested.

L 144-145: “A-E clade containing multiple sub-clades”

Suppl Figure 8: LD decay over 16-35 bp is extremely fast! Are you certain the scale is not in kbp?

It is not a special case, especially for insect species. Comparable findings can be found in *Apis mellifera* (Wallberg, 2014; Nature Genetics, 46: 1081-1088).

L195-212: I do not understand the eigenvector approach. What is this supposed to control for or what is this axis supposed to represent? Geography or differentiation? If this axis represents adaptation to insecticides, is it not confounded by geography as well (South East Asia and Oceania being close to each other)?

The axis here represents differentiation. But if a group of populations from one region showed distinguishable differentiation from other populations (not a result of drift), the underlying reasoning is likely adaptation. The interpretation in the main text has been revised accordingly.

L206: 1,179 genes is a very large number. These could include a large number of false positives. Can your GWAS approach give any idea of what percentage may be real?

GWAS does not give any idea of what percentage may be real. As indicated above, we are confident of the result generated by EigenGWAS. The global distribution of the nonsynonymous genotypes of three genes with point mutations, presented in Supplementary Fig. 11, does validate the robustness and power of the GWAS analysis by showing that majority of the mutants were identified in SEA and OC.

L209: Are these genes directly associated with insecticide resistance?

Some of the genes are related to reported resistance. The identified genes and pathways suggest that high genetic polymorphism enables *P. xylostella* to adapt to various environmental conditions. The sentence has been rewritten accordingly.

L221-225: How do the predicted structures inform you about enzymatic activities? Did any other genes show point mutations?

The effect of point mutations on enzymatic activities was predicted based on the contact and interaction between mutations and the surrounding residues. Only those genes/enzymes with potential conformational change were presented.

L500: 399 sequence, how do these add up to the 532 P. xylostella used (e.g. L 550)?

468 COI sequences (including 399 *P. xylostella* sequences) from previous publications were incorporated into our dataset.

L520-522: Were the artificial chromosomes constructed according to an existing linkage map?

The 20 artificially synthetic chromosomes were constructed to facilitate the SNP calling. Once our SNP dataset was available, following analyses were performed using the SNP dataset, regardless of artificial chromosomes.

L528-532: How does the baserecalibration work if the reference SNP set was extracted from the current dataset?

After realignment, base-quality scores were recalibrated using BaseRecalibrator based on the reference SNP set, which was generated using UnifiedGenotyper and SAMtools[49] from the 532 individuals.

L539-541: What average read coverage per genotype do these QC filter values result in?

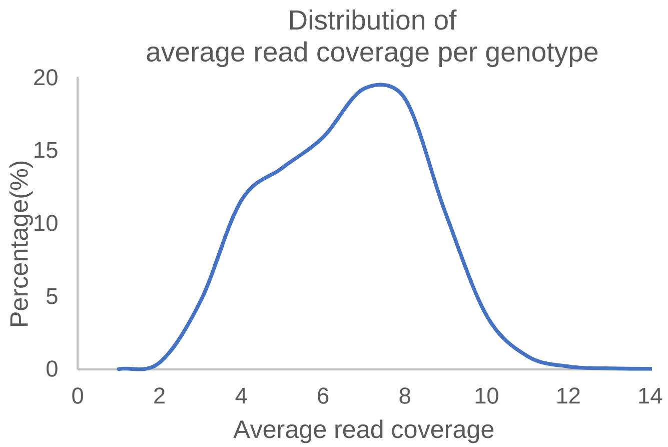

After filtering, we generated a dataset with average read coverage per genotype ranging from 3 to 10, accounting for 98% of the SNPs as shown in the above figure.

L 559: Phylogenetic tree: How many SNPs were eventually included in this tree? Have you considered using more advanced tree building methods for whole genome data using FastTree?

We used a total of 7,115,853 SNPs in the nuclear tree construction. NJ tree based on p-distance is conserved while robust, and has been applied in many resequencing project (e.g. rice, soybean). Other methods like ML is time consuming and may not improve the accuracy of our tree (i.e. for a species with high recombination rate). We used different filtering criteria to generate data-sets used in phylogeny construction. The used data-set were SNPs thinned from a interval of 25 bp across the genome with the aim to reduce the effects of recombination and also retain more information for phylogeny construction. The criterion applied in this study for SNP filtering was suggested by previous studies, such as Pilot et al. Genome-wide signatures of population bottlenecks and diversifying selection in European wolves. Heredity, 112: 428-442 (2014).

L599-600: What were the criteria used to define these clusters?

We defined these clusters based on the phylogenetic tree and geographic information.

L613-614: What arguments are there that the Drosophila mutation rate should match that of P. xylostella?

Drosophila mutation rate was the most commonly used as a standard and has been applied for other population genomics studies on Lepidoptera insects, e.g. monarch butterfly (Zhan et al., 2014).

Figures:

The color schemes in Figure 1A, 1C and 2A should match, which is not the case.

Revised as suggested

Figure 1A. Perhaps points can be sized according to the number of samples from each population.

We did not size the points according to the number of samples from each population in Fig. 1a, but that was done in Fig 3a.

Figure 1B. This plot should have a legend and y-axis labels. How are SNPs and indels scaled here? Also, the unassigned proportion is large. How are these scaffolds ordered and how should one interpret patterns of changes in nucleotide diversity here? Perhaps the spatial information of the unordered scaffolds is confusing and unnecessary and it might be better to remove this information and only show average for the unordered scaffolds.

A scale bar has been provided as suggested.

Figure 1C. The legend here is redundant. I would just add an x-axis title.

Revised as suggested.

Figure 2A. Why not present the rooted tree, given that you included an outgroup?

We did present a rooted tree in Figure 2A.

Figure 3A. What is the scale for the pie chart sizes related to the sampling size? The moth on top if the figure is hard to see. How are the clusters A-E identified? Why are no clusters like that identified in the ancestral populations? In case of a recent spread, I would be surprised that a recently colonized area is so diverse.

The pie chart is sized according to the number of samples from each population.
As responded to previous comments, it is not uncommon that derived populations are more diverse than the ancestral populations for insect species, especially for such a pest with strong migratory capacity

References:

Furlong, M. J. Wright, D. J. & Dosdall, L. M. (2013). Diamondback moth ecology and management: problems, progress, and prospects. *Annu. Rev. Entomol*. 58, 517-541.

Henniges-Janssen, K., Reineke, A., Heckel, D.G. & Groot, A.T. Complex inheritance of larval adaptation in *Plutella xylostella* to a novel host plant. Heredity 107, 421–432 (2011).

Patton, A. et al. (2019). Contemporary demographic reconstruction methods are robust to genome assembly quality: a case study in tasmanian devils. *Molecular Biology and Evolution.* doi:10.1093/molbev/msz191. .

Pilot, M. et al. (2014). Genome-wide signatures of population bottlenecks and diversifying

selection in European wolves. *Heredity* **112**, 428-442.

Wallberg, A. et al. (2014). A worldwide survey of genome sequence variation provides insight into the evolutionary history of the honeybee *Apis mellifera*. *Nature Genetic* **46**, 1081-1088.

You, M. S. et al. (2013). A heterozygous moth genome provides insights into herbivory and detoxification. *Nature Genetics*. **45**, 220-225.

Zhan, S. et al. (2014). The genetics of monarch butterfly migration and warning colouration. Nature **514,** 317-321.

Reviewers' comments:

Reviewer #1 (Remarks to the Author):

The authors have satisfactorily addressed all my coments/concerns.

Reviewer #2 (Remarks to the Author):

The manuscript is improved from the last submitted version but many of problems I previously highlighted have not been fully addressed:

There are still some references to "Overrepresentation" (line 215) and "Enrichment" (line 441) even though an enrichment analysis was not performed. Line 215 should be changed to something like "The most highly represented pathways were...", similarly "Enrichment" in the figure legend should be replaced with something like "Representation" or "Top".

I don't think the results presented support these statements (lines 216-221):
"The majority of the 1,179 candidate genes are associated with environmental adaptation, detoxification of plant defense compounds and insecticide resistance through regulation of gene expression or mutation of target sites. These findings further clarify the genetic and molecular bases of how P. xylostella can rapidly evolve the capacity of xenobiotic detoxification."
As far as I can see only 3 of the genes are analysed in enough detail to say precisely what they do. This seems to be a massive over-interpretation of the results.

I don't agree that the results found here are similar to those found in honey bees (Wallberg, 2014; Nature Genetics, 46: 1081-1088). The increases in diversity found there are specifically associated with hybridisation following domestication and as far as I can tell there is not a signature of increases in diversity in the honey bee associated with range expansion. I think the pattern of increasing diversity found here is highly unusual and unexpected and needs some comment in the manuscript. One possible explanation might be hybridisation with other species as P. xylostella as spread, but this would need further exploration. The explanation of higher genetic diversity being due in some way to human use of pesticides doesn't make any sense since this would lead to strong selection and reduced diversity.

Figure 2a: It should at least state in the legend that the branch lengths are not scaled. This figure is currently misleading.

In the figure 4 legend, it isn't clear that 0.0169 is the genome-wide median Fst, rather than the median of these genes. I also think it would be clearer if this was shown as a line in the plot, or a separate box-plot with the genome-wide values given.

The new section about figure 4a (lines 209-212): "which indicates that numerical distribution of the significantly differentiated SNPs is related to the density of SNPs in each of the four genomic regions (Fig. 4a)." doesn't make sense. I also think it is not a particularly interesting point to highlight (essentially that the outlier SNPs appear to be randomly distributed with respect to genes). I would recommend moving figure 4a to the supplementary information.

Best wishes,
Nicola Nadeau

Reviewer #3 (Remarks to the Author):

The authors have revised their manuscript and responded to all comments provide to the reviewers. However, I feel that at least towards my comments, the adjustment have been minimal and some of the responses still leave me confused.

I am inclined to trust the authors’ assessment of being able to identify the recent population size changes, given the short generation times of the diamond back moth'. However, I think the manuscript would benefit from adding this reasoning to the methods, because I think in general it is exceptional to be able to find a fit with such recent changes.

I still think the reader could be better guided through how the authors come up with the sequence of dispersal events that have given rise to the current distribution of diamondback moth (Figure 3A), how it is extracted from the analysis and what results support these inferences.

I still do not understand how the EigenGWAS analysis works. I do not understand why picking a PCA axis that identifies geographic structure in the samples would identified selected loci in the GWAS analysis, I would imagine these loci could easily represent false positives that correlate with geography/drift, rather than selection. I’m sure I am missing something here and some additional explanations would probably resolve this.

It is not clear to me using which criteria the three genes with point mutations were selected to be discussed (as also noted by reviewer 1).

Responses to the Comments from Reviewers

Reviewer #1 (Remarks to the Author):

The authors have satisfactorily addressed all my comments/concerns.

Reviewer #2 (Remarks to the Author):

The manuscript is improved from the last submitted version but many of problems I previously highlighted have not been fully addressed:

There are still some references to "Overrepresentation" (line 215) and "Enrichment" (line 441) even though an enrichment analysis was not performed. Line 215 should be changed to something like "The most highly represented pathways were...", similarly "Enrichment" in the figure legend should be replaced with something like "Representation" or "Top".

We have changed "overrepresented in metabolic and molecular signaling-related pathways" to "the most highly represented in metabolic and molecular signaling-related pathways according to the GO and KEGG analysis (the top 20 GO terms and KEGG pathways are shown in Figs. 11b and 12)" in the text. We have also changed "Enrichment" to "Representation" in the figure legends of Supplementary Figs. 11b and 12, and changed "being overrepresented" to "the representative" of in the figure legend of Supplementary Fig. 13.

I don't think the results presented support these statements (lines 216-221):
"The majority of the 1,179 candidate genes are associated with environmental adaptation, detoxification of plant defense compounds and insecticide resistance through regulation of gene expression or mutation of target sites. These findings further clarify the genetic and molecular bases of how P. xylostella can rapidly evolve the capacity of xenobiotic detoxification."
As far as I can see only 3 of the genes are analysed in enough detail to say precisely what they do. This seems to be a massive over-interpretation of the results.

To address this comment, we have revised the relevant section in the main text to say precisely what we have done and ensure that our interpretations are well-founded. This reads as follows. "Among the 1,179 candidate genes under divergent selection, we found 93 that were known from the published *P. xylostella* genome[11] to function in detoxification of plant defense compounds and insecticide resistance. Next we identified six of these genes that had non-synonymous SNPs in coding regions. Three of these, carboxypeptidase A (Px005867), P450-CYP2 (Px002515), and juvenile hormone esterase (JHE, Px003448), have reliable structural templates available in the Protein Data Bank (PDB, http://www.rcsb.org), which allowed us to create homology models for these three enzymes using Schrödinger software[49]."

I don't agree that the results found here are similar to those found in honey bees (Wallberg, 2014;

Nature Genetics, 46: 1081-1088). The increases in diversity found there are specifically associated with hybridisation following domestication and as far as I can tell there is not a signature of increases in diversity in the honey bee associated with range expansion. I think the pattern of increasing diversity found here is highly unusual and unexpected and needs some comment in the manuscript. One possible explanation might be hybridisation with other species as P. xylostella as spread, but this would need further exploration. The explanation of higher genetic diversity being due in some way to human use of pesticides doesn't make any sense since this would lead to strong selection and reduced diversity.

Thanks for the comments on this key issue. We carefully considered the possibility of hybridization. Whilst Perry et al. (2018) showed that *Plutella* species occasionally hybridize under no-choice single pair mating experiments in the laboratory, whole genome sequencing of wild-caught specimens failed to identify evidence for hybridization or introgression between *Plutella* species in natural habitats (Ward & Baxter 2018). Accordingly, we do not think hybridisation is likely the explanation for our finding of higher diversity in the derived (invasive) populations than that in the source (native) populations. Rather, this is likely a result of the derived populations getting novel alleles by adapting to new ecologically-diverse environments and improving genetic diversity by population mixing and recombination in the expansion ranges[27-29]. As suggested, we have added a paragraph to the manuscript to explain the pattern of increasing genetic diversity in the range expansion process of *P. xylostella* populations. It reads: "*P. xylostella* has remarkable genetic plasticity[26] and high a level of genomic variation[11] that enable it to rapidly adapt to local environments, potentially leading to the change in the levels of genetic diversity among geographical populations. After a new founder event of *P. xylostella*, the sizes of derived populations tended to rapidly grow while the SA population size was decreasing based on the SMC++ analysis (Fig. 3a). This process may have led to accumulation of mutations that were not present in the ancestral population, especially reflecting the likelihood that the species would have been subject to new and diverse selection pressures from novel plant hosts, novel agonists (e.g., pathogens and parasitoids), habitats, and climates[27,29], as well as becoming established in dispersed geographical regions. Genetic admixture, which may generate novel genotypes[28,29], among the *P. xylostella* populations in both the Old World and North America was frequent according to our phylogenic reconstruction (Supplementary Fig. 3) and population structure analyses (Fig. 2b and c). Together, these effects help explain the pattern of increasing genetic diversity in the range expansion process of *P. xylostella* populations (Fig. 1c)."

To further investigate alternative explanations, we divided the genome[11] into 3,256 10kb tiled windows and constructed local trees for each, and then calculated topology weighting for each tree to determine which 6-tip topology based on geographical populations and species delimitation is most supported. This revealed the most prevalent local topologies across the genome support the whole genome phylogeny placement of *P. xylostella* populations of South America as the crown node of the *P. xylostella* clade (Supplementary Table 6). We also calculated d_{XY} values[22] on these 3,256 windows between each of the five identified *P. xylostella* populations and *P. australiana*. This showed that genetic differentiation is lowest between the outgroup *P. australiana and* South American population, followed by North American, African-Eurasian, South East Asian, and Oceanian populations (Fig. 2d). These two additional lines of evidence have been

added to the manuscript. One statement was added to further support South America as the crown node of the *P. xylostella clade* and the most likely geographical area of origin, as it reads: "We also generated summary trees of different groups (see population genetic structure analysis below) based on 3,256 genome-wide local trees and revealed that the most prevalent topologies across the genome support *P. xylostella* populations of South America as the basal node closest to *P. australiana* (Supplementary Table 6)." Another statement was added to further support the global dispersal events of *P. xylostella*, which reads: "The d_{XY}-based analysis[22] showed the lowest genetic differentiation between SA *P. xylostella* and *P. australiana*, followed by NA *P. xylostella* and *P. australiana*, A-E *P. xylostella* and *P. australiana,* SE Asian *P. xylostella* and *P. australiana,* and OC *P. xylostella* and *P. australiana* (Fig. 2d), which outlines the global colonization process of *P. xylostella* populations."

Figure 2a: It should at least state in the legend that the branch lengths are not scaled. This figure is currently misleading.

As suggested, we have indicated in the legend of Fig. 2a that the branch lengths are not scaled.

In the figure 4 legend, it isn't clear that 0.0169 is the genome-wide median Fst, rather than the median of these genes. I also think it would be clearer if this was shown as a line in the plot, or a separate box-plot with the genome-wide values given.

The figure and figure legend have been revised as suggested, with a yellow dotted line showing the genome-wide median F_{ST} (0.0169) in the plot of Supplementary Fig. 11 (or Fig. 4 in our previous submission of the manuscript).

The new section about figure 4a (lines 209-212): "which indicates that numerical distribution of the significantly differentiated SNPs is related to the density of SNPs in each of the four genomic regions (Fig. 4a)." doesn't make sense. I also think it is not a particularly interesting point to highlight (essentially that the outlier SNPs appear to be randomly distributed with respect to genes). I would recommend moving figure 4a to the supplementary information.

The sentence has been changed in the manuscript to "which indicates that numerical distribution of the significantly differentiated SNPs was proportionally similar to that of the filtered SNPs in each of the four genomic regions (Supplementary Fig. 11a)." Fig. 4a has been moved to the supplementary information (Supplementary Fig. 11a) as suggested.

Best wishes,
Nicola Nadeau

Reviewer #3 (Remarks to the Author):

The authors have revised their manuscript and responded to all comments provide to the reviewers. However, I feel that at least towards my comments, the adjustment have been minimal and some of the responses still leave me confused.

I am inclined to trust the authors' assessment of being able to identify the recent population size changes, given the short generation times of the diamond back moth'. However, I think the manuscript would benefit from adding this reasoning to the methods, because I think in general it is exceptional to be able to find a fit with such recent changes.

We have added the reasoning and a citation of reference to the Methods with an additional statement: "The short generation time of *P. xylostella* makes possible the reliable and precise estimation of effective population sizes in the recent past using the method of SMC++ [25]."

I still think the reader could be better guided through how the authors come up with the sequence of dispersal events that have given rise to the current distribution of diamondback moth (Figure 3A), how it is extracted from the analysis and what results support these inferences.

To determine the likely dispersal events of DBM, we drew from the neighbor-joining phylogeny based on the nuclear genomes (SNPs) of 532 *P. xylostella* individuals collected worldwide (Fig. 2a) showing that the South American specimens were most basal and those from Oceania most derived. This was supported by the population structure analysis using sNMF[63] (Fig. 2b) and the PCA plot of the first two components using PLINK[65] (Fig. 2c) based on filtered 2,839 SNPs, which showed South American and Oceania populations are most widely differentiated, with the populations from North America, Africa-Eurasia and SE Asia being intermediate. In addition, the d_{XY} values[22] between the five identified groups and the outgroup population showed the lowest genetic differentiation between SA *P. xylostella* and *P. australiana*, followed by NA *P. xylostella* vs *P. australiana*, A-E *P. xylostella* vs *P. australiana,* SE Asian *P. xylostella* vs *P. australiana,* and OC *P. xylostella* vs *P. australiana* (Fig. 2d). These lines of evidence allowed us to propose a pattern of dispersal events for *P. xylostella* (Fig. 3b).

We have also replaced the illustration of lineage profiling (shown with colored pies, Fig. 3A in our previous submission of the manuscript) by geographically clustered populations (shown with sampling sites, Fig. 3b). We have also used the red arrows to denote the proposed dispersal events of *P. xylostella* from South America towards other continents, indicating in the figure legend that it is based on phylogenetic and structure-related analyses (Fig. 2a, b and c), genetic differentiation among groups (*d*xy-based analysis) outlining the global colonization process of *P. xylostella* (Fig. 2d), and demographic analysis showing the historical population sizes and divergence times of different groups (Fig. 3a).

Based on your suggestion, we have added a paragraph in the text to better guide the reader on how we inferred the global colonization process of *P. xylostella*. It reads: "To determine the most probable dispersal events of *P. xylostella*, we drew from the neighbor-joining phylogeny based on the nuclear genomes (Fig. 2a), population structure analysis using sNMF[63] (Fig. 2b) and the PCA plot of the first two components using PLINK[65] (Fig. 2c), showing that SA and OC populations are most widely differentiated, with the populations from NA, A-E and SEA being intermediate. In addition, our *d*xy-based analysis of genetic differentiation[22] indicated that congeneric *P. australiana* had lowest genetic differentiation from *P. xylostella* in SA, followed by NA, A-E, SEA,

and finally OC (Fig. 2d). The demographic analysis showed that the SA lineage was basal with the NA, A-E and SEA lineages diverging at progressively later stages and OC lineage the most recent (Fig. 3a), supporting the findings of the preceding analyses. These lines of biological evidence were integrated with historical information on European colonization and trade routes[18-20], allowing us to propose a scenario of dispersal events for *P. xylostella* (Fig. 3b)."

I still do not understand how the EigenGWAS analysis works. I do not understand why picking a PCA axis that identifies geographic structure in the samples would identified selected loci in the GWAS analysis, I would imagine these loci could easily represent false positives that correlate with geography/drift, rather than selection. I'm sure I am missing something here and some additional explanations would probably resolve this.

The algorithm of EigenGWAS was developed to identify single SNPs that contribute to the genetic differentiation (eigenvector) of two populations based on regression analysis. By using individual-level eigenvectors as phenotypes (Y in regression analysis) and single SNPs (X in regression analysis) in a linear regression, the resulting regression coefficients are equivalent to singular value decomposition (SVD) SNP effects and used to identify loci under selection along gradients of ancestry[48]. EigenGWAS also used a correction parameter to filter out signals of population stratification (i.e. caused by geography/drift), which allow the loci under selection to be identified. This approach has been successfully used to identify the loci under divergent selection between the UK and Dutch populations of the great tit (*Parus major*) for better understanding of how genetic signatures of selection translate into variation in fitness and phenotypes[38].

To help the reader understand the approach, we have added these statements in the section of Methods as you suggested.

It is not clear to me using which criteria the three genes with point mutations were selected to be discussed (as also noted by reviewer 1).

We have added a paragraph in the main text to describe how the three genes with point non-synonymous mutations were selected for discussion. It reads: "Among the 1,179 candidate genes under divergent selection, we found 93 that were known from the published *P. xylostella* genome[11] to function in detoxification of plant defense compounds and insecticide resistance. Next we identified six of these genes that had non-synonymous SNPs in coding regions. Three of these, carboxypeptidase A (Px005867), P450-CYP2 (Px002515), and juvenile hormone esterase (JHE, Px003448), have reliable structural templates available in the Protein Data Bank (PDB, http://www.rcsb.org), which allowed us to create homology models for these three enzymes using Schrödinger software[49]".

References:

Perry, K. *et al*. Cryptic *Plutella* species show deep divergence despite the capacity to hybridize. *BMC Evol. Biol.* **18**, 77 (2018).

Ward, C. M. & Baxter, S. W. Assessing genomic admixture between cryptic *Plutella* moth species following secondary contact. *Genome Biol. Evol.* **10**, 2973-2985 (2018).

REVIEWERS' COMMENTS:

Reviewer #2 (Remarks to the Author):

The authors have satisfactoraly responded to all my comments.

I have just 2 minor suggestions for improvement.

1. I find the new section that has been added on lines 181 to 193 to be somewhat repetitive of information elsewhere. Lines 186 to 188 in particular seem very similar to lines 148 to 152. I realise this has been added in reponse to the criticism that the evidence supporting figure 3b is hard to follow. Perhaps therefore this information could be summarised and placed in the figure 3 legend or editied to be shorter and less repetitive?

2. I think "over-represented" should be removed from line 271

Best wishes,
Nicola Nadeau

Reviewer #3 (Remarks to the Author):

I thank the authors for thoughtfully addressing all my comments. I have no further remarks or questions.

Responses to the Comments from Reviewers

REVIEWERS' COMMENTS:

Reviewer #2 (Remarks to the Author):

The authors have satisfactorily responded to all my comments.

I have just 2 minor suggestions for improvement.

1. I find the new section that has been added on lines 181 to 193 to be somewhat repetitive of information elsewhere. Lines 186 to 188 in particular seem very similar to lines 148 to 152. I realise this has been added in reponse to the criticism that the evidence supporting figure 3b is hard to follow. Perhaps therefore this information could be summarised and placed in the figure 3 legend or editied to be shorter and less repetitive?

Reply: As suggested, the new section has been edited to be shorter and less repetitive. It now reads: "Our phylogenetic and genetic analyses, PCA plot of the first two components, and d_{xy}-based analysis of differentiation (Fig. 2) supported the demographic evidence that the SA lineage was basal with the NA, A-E and SEA lineages diverging at progressively later stages and the OC lineage the most recent (Fig. 3a). These results were integrated with historical information[18-20], allowing us to propose a scenario of dispersal events for *P. xylostella* (Fig. 3b)."

2. I think "over-represented" should be removed from line 271

Reply: "over-represented" has been removed.

Best wishes,
Nicola Nadeau

Reviewer #3 (Remarks to the Author):

I thank the authors for thoughtfully addressing all my comments. I have no further remarks or questions.
